# Thiol-selective native grafting from polymerization for the generation of protein–polymer conjugates†

**DOI:** 10.1039/d4sc04818k

**Published:** 2024-09-18

**Authors:** Melina I. Feldhof, Sandro Sperzel, Lorand Bonda, Susanne Boye, Adam B. Braunschweig, Ulla I. M. Gerling-Driessen, Laura Hartmann

**Affiliations:** a Department of Organic and Macromolecular Chemistry, Heinrich-Heine-University Düsseldorf Universitätsstraße 1 40225 Düsseldorf Germany; b Center Macromolecular Structure Analysis, Leibniz-Institut für Polymerforschung Dresden Hohe Str. 6 01069 Dresden Germany; c Advanced Science Research Center, Graduate Center, City University of New York 85 St. Nicholas Terrace New York NY 10031 USA; d PhD Programs in Chemistry and Biochemistry, Graduate Center, City University of New York 65 5th Avenue New York NY 10016 USA; e Department of Chemistry, Hunter College 695 Park Avenue New York NY 10065 USA; f Institute for Macromolecular Chemistry, University of Freiburg Stefan-Meier-Str. 31 D-79104 Freiburg i.Br. Germany laura.hartmann@makro.uni-freiburg.de ulla.gerling-driessen@makro.uni-freiburg.de

## Abstract

Protein–polymer conjugates combine properties of biopolymers and synthetic polymers, such as specific bioactivity and increased stability, with great benefits for various applications from catalysis to biomedicine. Furthermore, polymer conjugation can mimic important posttranslational modifications of proteins such as glycosylation. There are typically two approaches to create protein–polymer conjugates: the protein is functionalized in advance with an initiator for a *grafting-from* method or a previously produced polymer is conjugated to the protein *via* a *grafting-to* method. In this study, we present a new approach that uses native proteins and allows for direct *grafting-from* using a thiol-induced, light-activated controlled radical polymerization (TIRP) that is initiated at thiols from specific cysteine residues of the protein. This straightforward method is employed to introduce polymers onto proteins and enzymes without any prior protein modifications, it works in aqueous buffer and maintains the protein's native structure and activity. The resulting protein–polymer conjugates exhibit high molar masses and low dispersities. We demonstrate the versatility of this approach by introducing different types of polymers such as hydrophilic poly(2-hydroxyethyl acrylate) (pHEAA), temperature-responsive poly(*N*-isopropylacrylamide) (pNIPAM) as well as glycopolymers mimicking the natural protein glycosylation and enabling selective interactions. We present successful combinations of the protein and polymer functions *e.g.*, temperature-induced aggregation leading to an increase in enzyme activity and the introduction of artificial glycosylation inducing specific protein–protein cluster formation and giving straightforward access to glycosurfaces. Based on this straightforward, potentially scalable yet highly controlled synthesis of protein–polymer conjugates, various areas of applications are envisioned ranging from biomedicine to material sciences.

## Abstract 2

TIRP enables direct *grafting-from* polymerization of proteins and enzymes under physiological conditions, maintaining their structure and function. By using cysteine thiols as initiators, polymers are site-selectively grafted from unmodified proteins.

## Graphical abstract

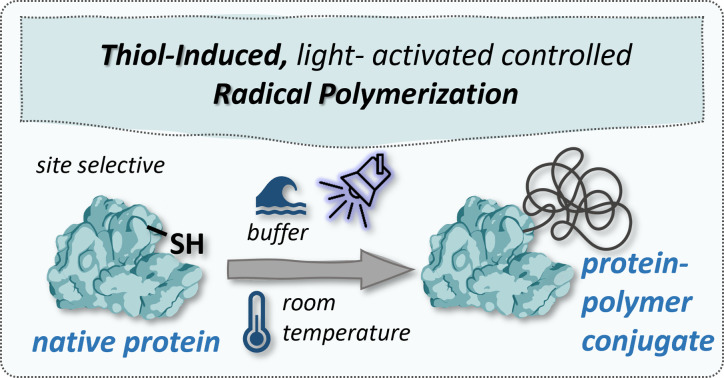

## Introduction

Protein–polymer conjugates are materials with a broad spectrum of applications in biotechnology, catalysis or even laundry detergents due to their improved properties, such as salt compatibility, storage stability and resistance to high temperatures or low pH conditions.^1–8^ The first PEGylated BSA conjugates found commercial applications as immunogenicity reducing drugs already in 1977.^9^ Today, at least 15 protein-PEG conjugates have been approved by the Food and Drug Administration (FDA) for therapies against diseases including hepatitis C, leukemia, blood cancer or autoimmune diseases.^9,10^ Protein–polymer conjugates are obtained by covalent linkage of a polymer and protein of choice, while retaining the protein's natural structure and function.^11–14^ Protein–polymer conjugates have opened new opportunities, especially in biomedical applications, as they offer a unique opportunity of salvaging the biological activity of native proteins and combining this with the advantageous properties of synthetic polymers, such as improved solubility, prolonged circulation time in the blood stream, reduced immunogenicity and improved biocompatibility.^15–19^ Furthermore, conjugation of polymers can mimic posttranslational modifications of proteins such as their glycosylation. This has been explored extensively through the introduction of hydrophilic polymers such as poly(ethyleneglycol) (PEG) *e.g.*, to increase protein stability.^20–24^ Using biofunctional polymers such as glycopolymers in protein–polymer conjugates to also affect bioactivity through the polymer component of the conjugate has been studied much less so far.^25–31^

The synthesis of protein–polymer conjugates distinguishes between *grafting-to* and *grafting-from* approaches.^32,33^ The *grafting-to* method involves the synthesis of a polymer followed by a subsequent conjugation of the polymer to the protein, allowing a simple and straightforward polymer synthesis and characterization. The major challenges with this method are low conjugation efficiency due to the high steric demand of the two components and complex purification procedures to separate conjugation products carrying different numbers of polymer chains on one protein.^32,33^ In contrast, the *grafting-from* approach uses polymerization from the protein *via* an initiator that is previously inserted into the protein structure. This typically results in higher yields of the protein–polymer conjugate due to lower steric demand of the monomers and growing chain during polymerization.^32,33^ However, the major limitation of this approach is the potential denaturation and inactivation of proteins due to the chemical conditions *e.g.*, organic solvents and higher temperatures, during the introduction of the initiator moiety or the polymerization itself.^34,35^ Recent work by Maynard *et al.*,^36^ Haddleton *et al.*,^37^ Velonia and Anastasaki *et al.*^13^ and Matyjaszewski *et al.*^38^ circumvents this and showed the successful conjugation of an initiator and later polymerization also in aqueous buffer solutions. Another important development has been the use of photo-induced polymerization methods to derive protein-conjugates by light at specific wavelengths can minimize potential damage to the protein structure, thus preserving bioactivity.^39–41^ Nevertheless, these processes are still two-step procedures and the immediate use of the protein as *e.g.*, isolated from a biotechnological process or biological sources is not possible. For both grafting methods, protein modification usually proceeds *via* the functional groups of the amino acids side chains, such as lysines or cysteines, that can react with succinimides, thiols, amines, or carboxylic acids.^1,34,42^ Since most proteins present several of these residues, this typically results in a mixture of products where conjugation sites and numbers can vary and such dispersity can limit applications *e.g.*, in biomedicine.^16^ In contrast, site-specific functionalization involves the insertion of bioorthogonal groups at specific amino acids *e.g.*, the N- or C-terminus, free cysteines, disulfides, or at selected sites that allow insertion of unnatural amino acids to enable selective and efficient initiator or polymer conjugation, but these approaches often require advanced synthetic methodologies.^15,16^

In addition to the positioning and number of polymer chains in the conjugates, polymer chain length, dispersity and composition (*e.g.*, copolymers and functional side and end groups) needs to be controlled. As controlled polymerization methods for the preparation of protein–polymer conjugates, mainly atom transfer radical polymerization (ATRP),^43–48^ reversible addition–fragmentation chain-transfer polymerization (RAFT),^49–52^ single electron-transfer living radical polymerization (SET-LRP),^37^ photoinduced electron/energy transfer reversible addition–fragmentation chain transfer polymerization (PET-RAFT)^41^ and ring opening polymerization (ROP)^53^ have been used. Ideally, the conjugated polymer should not significantly modify the structure of a native protein or in the case of an enzyme interfere or block the active site, yet at the same time the polymer should bring in additional properties and functions, such as solubilization or shielding.^14,54^ Today, the design and synthesis of protein–polymer conjugates are still subject to extensive research that aims at achieving site-specific conjugation, stoichiometric control, and high-yield conjugation products with new and advanced function,^55^ and also continue to be pursued to gain new insights into protein–protein interactions.^56,57^

**Fig. 1:**
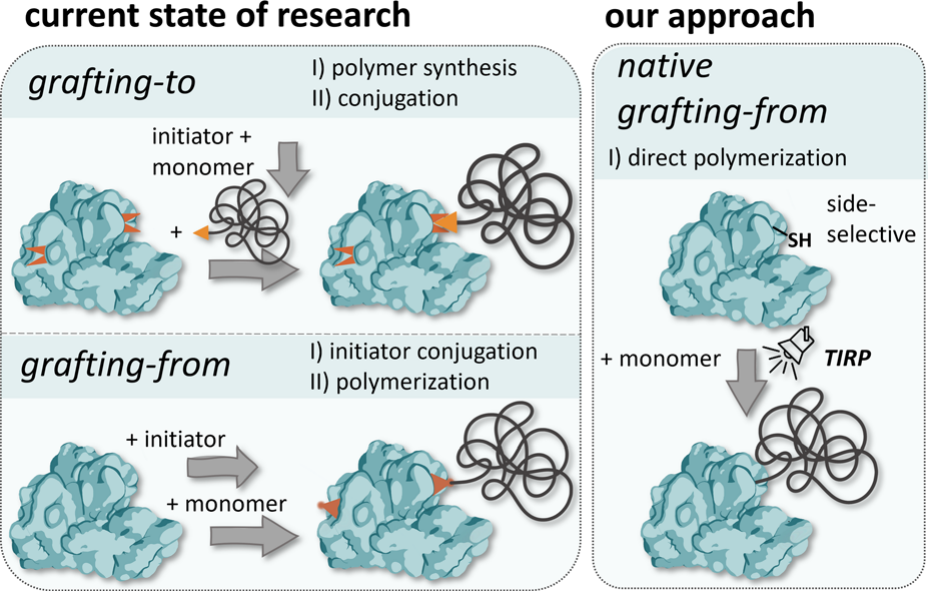
Overview of well-known systems for the preparation of protein–polymer conjugates and comparison with our approach.

Here, we introduce for the first time a *grafting-from* approach using native proteins without the need for any prior modifications while still enabling site-selective and highly controlled polymerization by employing a thiol-induced, light-activated controlled radical polymerization (TIRP) (Fig. 1). Our approach uses gentle polymerization conditions, suitable for a variety of proteins, potentially also less stable proteins, retaining their native structure and thus function, and allows for site selective modification addressing free thiols, which gives access to highly controlled protein–polymer conjugates.

## Results and discussion

**Fig. 2:**
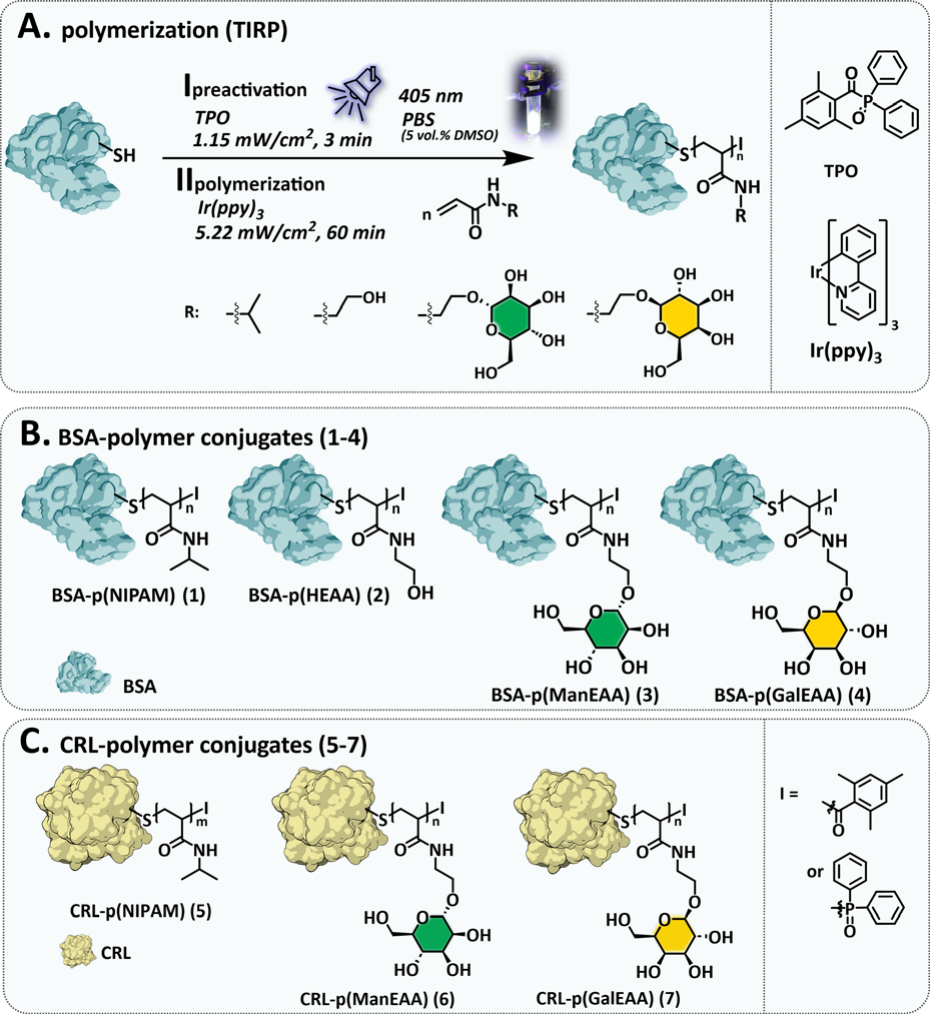
(A) Illustration of the general set up for the synthesis of TIRP based protein–polymer conjugates and approach values. (B) BSA based protein–polymer conjugates 1–4. (C) CRL based enzyme–polymer conjugates 5–7.

The recently introduced TIRP is a potentially controlled radical polymerization that can be initiated from various low molecular weight thiol initiators, such as tritylthiol.^58^ We now explore this method for the first time on high molecular weight thiol initiators, specifically the free thiols of cysteines in a protein (Fig. 2A). We chose *bovine serum albumin* (BSA) as protein initiator, as it contains exactly one free cysteine at position ′34.^59^ Four different acrylamide derivatives were chosen as monomers based on previously optimized TIRP conditions and to demonstrate the versatility of our approach in generating protein–polymer conjugates of different types. Specifically, p(HEAA) as a well-established hydrophilic, biocompatible polymer with non-immunogenic, anti-fouling, and non-toxic properties,^60–62^ p(NIPAM) as a non-toxic and temperature-responsive polymer^63–66^ and two glycopolymers derived from mannose (Man) and galactose (Gal) acrylamide monomers to install artificial glycosylation^67^ were polymerized from BSA (Fig. 2B). In addition to using BSA as an initiator, we chose the enzyme *Candida rugosa lipase* (CRL), with one free cysteine at position ′217 ^68^ to test whether both, the protein structure as well as its function (enzyme activity) are maintained after polymer conjugation (Fig. 2C). BSA is already a well-established protein known for its particularly good adhesion properties and CRL was selected for its reliable evidence of enzymatic activity. These two proteins were also chosen because of their free accessible thiol. However, this approach is not limited to proteins that already carry free cysteines. Recombinant protein expression methods allow easy modification of protein sequences, offer a straightforward opportunity to introduce cysteine residues as natural amino acids into a protein structure^69–71^ and thus make them amenable to the here presented native *grafting-from* approach.

TIRP-based native *grafting-from* polymerization on the proteins was carried out at room temperature in nitrogen-deoxygenated phosphate buffer (10 mM, pH 7.4). The photoinduced radical polymerization takes place under deoxygenated conditions to minimize oxygen-induced side reactions. The radical initiator diphenyl(2,4,6-trimethylbenzoyl)phosphine oxide (TPO) and tris(2-phenylpyridine)iridium, iridium catalyst were pre-dissolved in DMSO and diluted with the buffer to obtain a final concentration of 5% DMSO, which is known to not affect the native structure of proteins.^72^ In the pre-activation step I (Fig. 2A) the TPO/protein mixture is irradiated at low intensity (1.15 mW cm^−2^) for three minutes to form the activated protein initiator, immediately followed by step II, adding monomer and Ir catalyst and irradiating for one hour (5.22 mW cm^−2^). The formed protein–polymer conjugates were isolated by dialysis against PBS buffer.

**Fig. 3:**
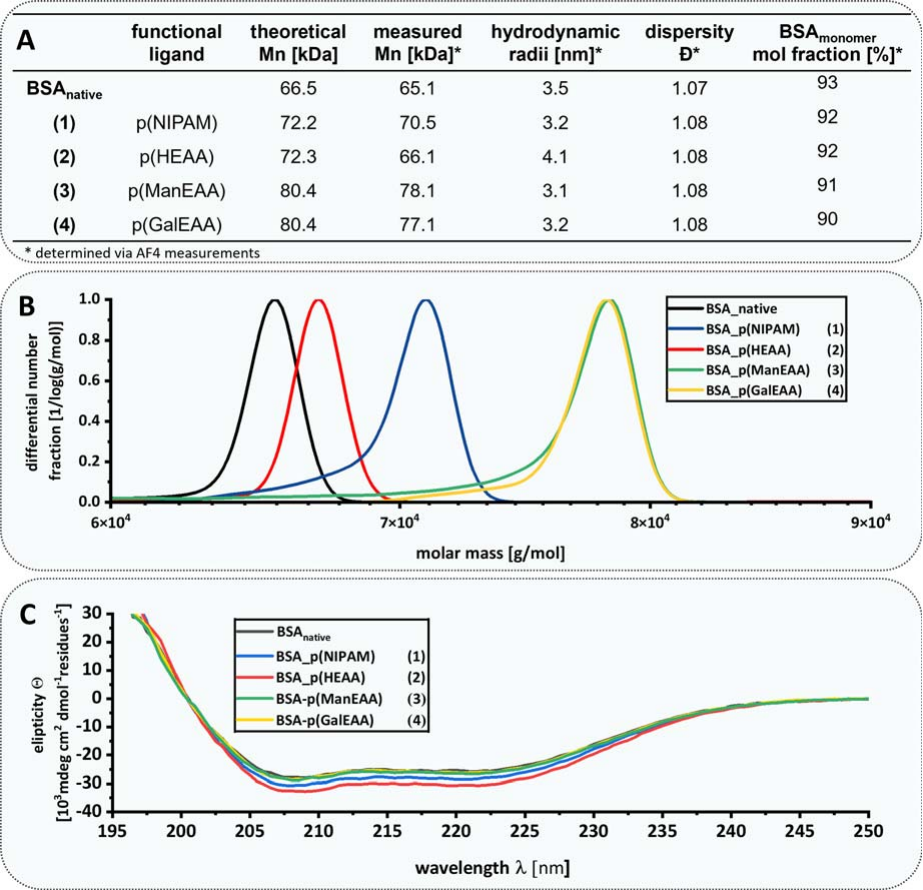
(A) Summary of analytical data for monomeric BSA population of protein–polymer conjugates 1–4*via* AF4-LS in PBS buffer (pH 7.4 at 25 °C). (B) Molar mass distributions obtained by AF4-LS: comparison of the individual molar masses of monomeric BSA population protein–polymer conjugates 1–4. (C) Presentation of CD spectra of BSA–polymer conjugates 1–4 for investigation of structural properties. The measurements were performed in PBS buffer (pH 7.4, at 20 °C) with an exact concentration of 251 µg mL^−1^ adjusted by the Pierce assay.

**Fig. 4:**
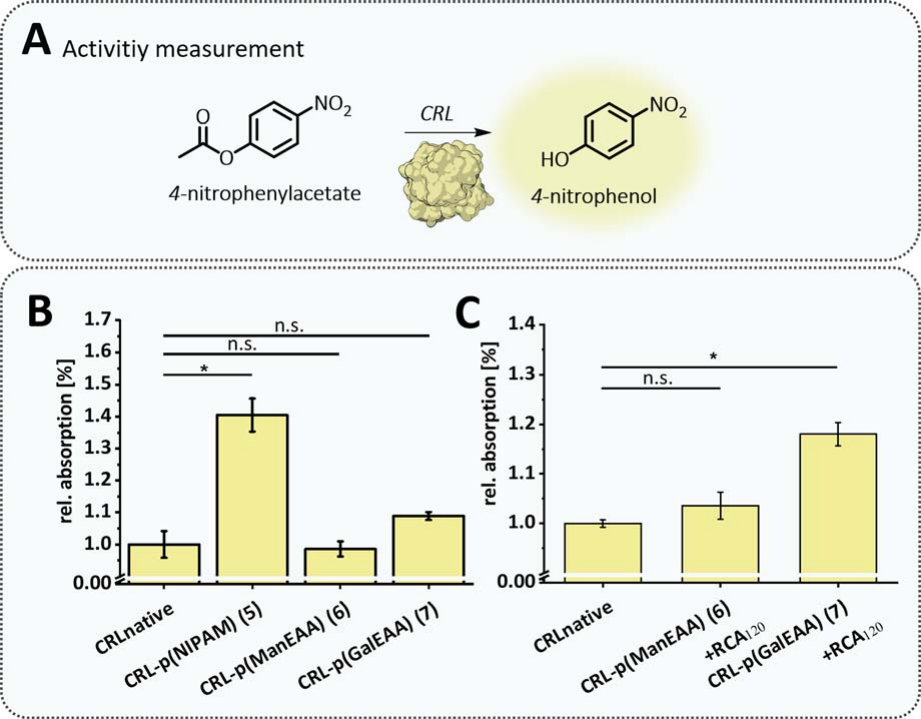
(A) Illustration of the activity/ester cleavage of the enzyme CRL. All thiol detections were performed in reaction buffer: 0.1 M sodium phosphate, pH 8.0, containing 1 mM EDTA. Ellman's stock solution of 4 mg mL^−1^ Ellman's reagent and a 0.75 mM protein stock solution were prepared in reaction buffer. The assay is performed in triplicate with an incubation time of 15 min and absorbance is measured at 412 nm. (B) Comparison of the activity of the native enzyme CRL and the conjugates 5–7. (C) Comparison of native CRL with CRL–polymer conjugates 6 and 7 incubated with lectin RCA_120_. Data were evaluated using one-way ANOVA analysis followed by Bonferroni correction (* <0.05).

Details on the characterization of the conjugates by ^1^H-NMR, IR, asymmetrical flow field flow fractionation with light scattering detection (AF4-LS), SDS-PAGE, Ellman's Assay, circular dichroism (CD) and differential scanning calorimetry (DSC) are displayed in the ESI.†^1^H-NMR and IR spectroscopy were used to demonstrate the successful formation of the different acrylamide polymers. The separation of protein–polymer conjugates is usually challenging due to high number of different functionalities. The use of AF4-LS^56,73^ enabled us to efficiently separate the three populations of BSA (monomeric BSA, BSA dimer or trimer/multimer), BSA–polymer conjugates and allowed the determination of absolute molar masses, dispersities and hydrodynamic radii (see ESI†). This gentle separation technique reduces sample degradation risk and permits the analysis of sensitive biomolecules like proteins and nucleic acids, which could be damaged by the shear forces in GPC.^74^ The shift towards increased molar masses verified the successful attachment of the polymers to BSA 1–4 (Fig. 3A). Furthermore, AF4-LS measurements confirmed that the protein–polymer conjugates 1–4 obtained molar masses in the range that were expected for the conjugation of one polymer chain per BSA according to a controlled polymerization. Molar mass distributions (Fig. 3B) showed a subtle shoulder for conjugates 1, 3, and 4 in the lower molar mass region, which indicates the presence of some remaining unconjugated BSA. This is explained by the fact that not all BSA molecules present free thiols. In agreement with literature values,^75^ approximately 54% of the theoretically available thiols of BSA were detected as accessible initiators for polymerization by using Ellman's assay.^76^ After polymer conjugation, we observed a significant decrease of free thiols (Fig. S47†). CD measurements^77^ confirmed that the native structure of BSA is maintained (Fig. 3C). Thermal protein denaturation of the protein–polymer conjugates determined the degree of unfolding by measuring the ellipticity at 222 nm at increasing temperatures. The results demonstrated no changes in protein stability for the conjugates 2–4 and a rather small increase in stability for the BSA–p(NIPAM) (1) conjugate (Fig. S42†). In addition, DSC measurements were performed (see ESI†) confirming an increase in thermal stability for the conjugate 1, which is in accordance with previous observations for other, similar protein–polymer conjugates.^78,79^ In order to analyze the polymer alone without the protein it was grafted from, protein–polymer conjugates were subjected to thermal amino acid degradation and the polymer was subsequently isolated by dialysis and analyzed by ^1^H-NMR and IR (see data for BSA–p(NIPAM) conjugate 1 in the ESI, Fig. S69 and S70†). For conjugates 2–4 pHEAA and glycopolymers were also degraded under the applied conditions and therefore did not allow for additional analysis of the polymer alone (data not shown). To further demonstrate that polymer conjugation maintains not only the structure but also function of the protein, we chose an enzyme – lipase CRL – for polymer conjugation *via* TIRP. CRL is widely used in pharmaceutical and biotechnological applications due to its selective hydrolysis of ester bonds.^80–82^ Three CRL–polymer conjugates carrying either p(NIPAM) (5), or glycopolymers p(ManEAA) (6) or p(GalEAA) (7) were prepared. CRL activity was determined by photometric quantification of the hydrolysis of *p*-nitrophenol acetate to *p*-nitrophenol and acetic acid (Fig. 4A).^83^ Correct conformation as well as an accessible active site are vital factors for proper enzyme function.^81,82^

To directly compare activity of the native enzyme and the respective polymer conjugates, measured adsorption values of the activity assay were normalized to the standard activity of the native, unconjugated enzyme, which represents 100% native activity. We observed that the enzyme activity of CRL remained unaffected in the conjugates 6 and 7 carrying the glycopolymers (Fig. 4B). However, we noticed a significant increase of about 40% in the activity for the CRL–p(NIPAM) conjugate 6. A similar effect was previously observed for CRL–p(NIPAM) conjugates in non-aqueous solvents obtained by a *grafting-to* method.^84^ The authors explain this by the hydrophobic character of the polymer, which interacts with the CRL lid and thus supports a conformation that is suitable for catalysis. We attribute our own observation to another phenomena, possibly in addition to the previous suggestion, and to the fact that the activity assay was carried out at 30 °C, which is close to the LCST of the CRL–p(NIPAM) conjugate 5 (32 °C, Fig. S56†). The increase in the activity at elevated temperatures could be the result of cluster formation of this conjugate.

This finding inspired us to start looking into different areas of applications for the protein–polymer conjugates now available from the native *grafting-from* method. Applications have been chosen that demonstrate the successful preservation as well as combination of properties of both, protein and polymer. First, to confirm aggregation induced increase in enzymatic activity of CRL, we used the galactose-specific lectin *Ricinus communis agglutinin I* (RCA_120_) to induce clustering of the CRL–p(GalEAA) conjugate (7) while the CRL–p(ManEAA) conjugate (6) served as negative control with RCA_120_ not binding to Man (Fig. 4C). Cluster formation between CRL–p(GalEAA) (7) and RCA_120_ was confirmed by turbidity measurements, while CRL–p(ManEAA) (6) showed no interaction with RCA_120_ (Fig. S53†). Indeed, we observed that the enzymatic activity of the CRL–p(GalEAA) conjugate increased significantly, by approximately 20%, when clustering is induced by the lectin, whereas the activity of the CRL–p(ManEAA) conjugate remained unaffected. Thus, conjugation of the glycopolymer enabled not only an increase in stability but the specific formation of a protein–protein complex and thus tuning the enzyme activity.

Indeed natural glycosylation of proteins plays a key role for their structure, function and the recognition of other interaction partners.^34,85^ Glycopolymers are simple mimetics of natural glycoconjugates and have found wide application in biomedical research *e.g.*, for the detection and inhibition of pathogens, in tumor biology and tissue engineering.^29,34,67,86,87^ To this end, conjugation of glycopolymers to proteins can serve as “artificial” glycosylation.^34^ Indeed, protein–glycopolymer conjugates have demonstrated great potential for various biomedical and biotechnological applications *e.g.*, in vaccine development and drug delivery.^25^ TIRP-based native *grafting-from* approach now gives access to protein–glycopolymer conjugates in a one-step procedure and enables the combination of the protein's properties and function with the specific binding of the glycopolymer.

**Fig. 5:**
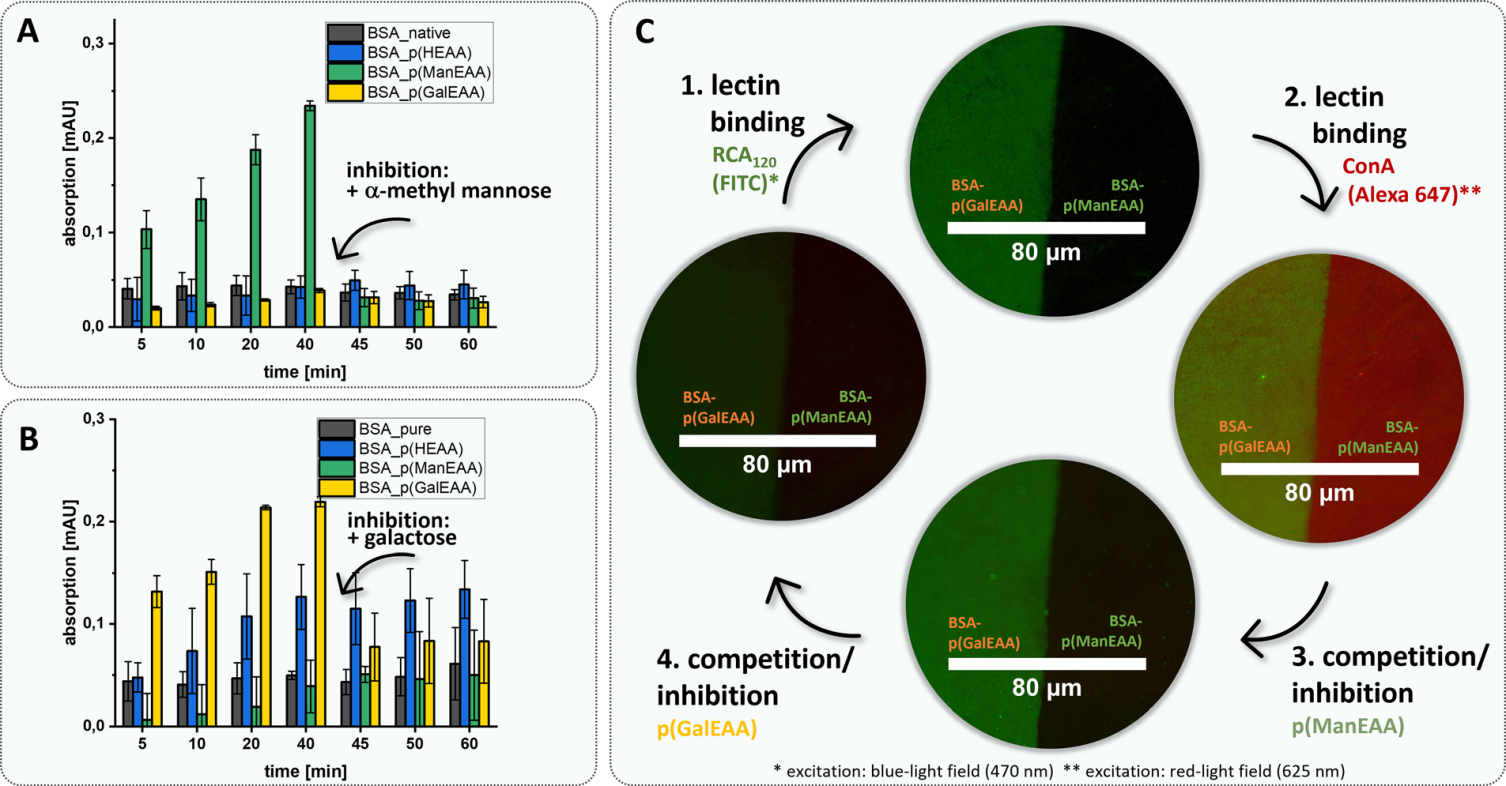
Presentation of ligand–receptor competition inhibition assay in solution and on surface. (A) Turbidity measurements were performed at 420 nm at room temperature (20 °C). 120 µL of all structures S5, 2, 3 und 4 (5 µM) were mixed with ConA (50 µM) and measured after 5, 10, 20 and 40 min. Inhibitor p(ManEAA) S7 (30 mM) was then added and measured for another 5, 10, and 20 min. (B) Turbidity measurements were performed at 420 nm at room temperature (20 °C). 120 µL of all structures S5, 2, 3 und 4 (5 µM) were mixed with RCA_120_ (50 µM) and measured after 5, 10, 20, and 40 min. Inhibitor p(GalEAA) S8 (30 mM) was then added and measured for another 5, 10, and 20 min. (C) Microscopic images of the surface ligand–receptor interaction using the half-side dip approach with conjugate 4*vs.*3 (1 mg mL^−1^ in LBB buffer). The microscope images are a stack of blue (470 nm)– and red (625 nm)-light excitation. (1) The half–half carbohydrate coated surface was first incubated with the Gal-recognized lectin RCA_120_-FITC (5 µM) for 30 min. (2) After washing the surface with LBB it was incubated with Man-recognized lectin ConA-Alexa 647 (5 µM) for another 30 min. The following inhibition step (3) with p(ManEAA) S7 (15 mM) and (4) with p(GalEAA) S8 (15 mM) can also be comprehended microscopically.

To further demonstrate this, we used the two different BSA–glycopolymer conjugates BSA–p(ManEAA) (3) and BSA–p(GalEAA) (4) to study the interactions of the protein–polymer conjugate with two lectins – Gal-recognizing RCA_120_ and Man-recognizing *Concanavalin A* (ConA). Selective binding and protein–protein complex formation was studied by turbidity measurements^88,89^ demonstrating cluster formation between BSA–p(ManEAA) (3) and ConA, and between BSA–p(GalEAA) (4) and RCA_120_, respectively. To confirm that cluster formation is driven by the glycopolymer–lectin interactions, α-methylmannose and galactose were used as competitive inhibitors enabling full dissolution of the previously formed clusters (Fig. 5A and B). The strength of carbohydrate–lectin interaction can be estimated by determining the concentration (5.61 ± 0.38 µM) at which the half-maximal turbidity is reached for BSA–p(ManEAA) conjugate (3) (Fig. S44†). Considering that approximately 84% of the molecular weight of the conjugate is the protein, it is feasible to determine a value for the half-maximal turbidity that solely pertains to the carbohydrate units, amounting to 0.94 ± 0.06 µM (Fig. S45†). This value of the BSA–p(ManEAA) conjugate is similar to the concentration of pure p(ManEAA) polymer in solution (1.05 ± 0.06 µM) (Fig. S46†), suggesting that an equivalent binding affinity was achieved with the same amount of Man units in solution as in the conjugate. Hence, the conjugation did not affect the binding affinity of the carbohydrate ligands.

BSA is often used in the passivation of surfaces based on its high adherence *e.g.*, to glass. Here we made use of this property to demonstrate an easy and effective immobilization of our protein–glycopolymer conjugates on glass surfaces and derived glycofunctionalized surfaces. Glycofunctionalized surfaces are commonly used *e.g.*, in glycan arrays^90^ or for the detection and isolation of pathogens and tumor cells^91–93^ but usually require more complex synthetic procedures. Using a simple half-coating dip approach (schematic representation in the ESI, S60†), either native BSA and a BSA–glycopolymer conjugate or combinations of both BSA–glycopolymer conjugates were adsorbed next to each other on a glass surface. Fluorophore-labeled lectins ConA (Alexa 647) and RCA_120_ (FITC) were used to determine the specific glycopolymer–lectin interaction *via* fluorescence microscopy. Inhibition competition assays using soluble glycopolymers p(ManEAA) (*S7*) and p(GalEAA) (*S8*) were performed to confirm selectivity of the protein–glycopolymer interactions. While in solution, the addition of the monosaccharide-based inhibitors (α-methylmannose and galactose) resulted in dissociation of the conjugate-lectin clusters, the addition of these inhibitors to the surfaces did not result in the release of the respective lectins from the BSA–polymer conjugates. Presumably, the dense presentation of multivalent glycopolymer ligands on the surface requires stronger or multivalent competitors to disrupt the lectin binding.^94^ Furthermore, steric shielding of the glycopolymer ligands, as well as favoured statistical rebinding events, are likely factors to contribute to the strong lectin binding on glycopolymer-presenting surfaces. Therefore, as inhibitors, glycopolymers p(ManEAA) (*S7*) and p(GalEAA) (*S8*) were employed to enable competitive lectin release from the glycopolymer-coated surfaces (Fig. 5).

Fluorescence microscopy confirmed homogeneous surface functionalization and simultaneous yet specific binding of RCA_120_-FITC and ConA-Alexa 647 to the BSA–p(GalEAA) (4) and BSA–p(ManEAA) (3) coated parts of the surface, respectively. Through inhibition with soluble glycopolymers, either of the lectins can be released from the surface in presence of the other lectin staying bound. Thus, this demonstrates the straightforward use of BSA–glycopolymer conjugates to derive glycofunctionalized surfaces for specific ligand-mediated receptor interactions.

## Conclusions

In summary, protein–polymer conjugates are an important class of biomolecules with a great variety of applications in the biomedical or the material sciences. Yet, deriving protein–polymer conjugates with currently available *grafting-to* and *grafting-from* polymerization methods is limited by low yields, poor conjugation site control and the need for artificial initiators. Here, we now present a mild *grafting-from* polymerization method using the native protein for the generation of various protein–polymer conjugates. We show that the native structure and function of proteins is retained and is suitable for the conjugation of various polymers including high molecular weight glycopolymers giving straightforward access to introducing artificial glycosylation. We already demonstrate a variety of potential applications of protein–polymer conjugates and protein–glycopolymer conjugates which can be further extended into areas such as catalysis, material sciences, biotechnology and biomedicine which can now benefit from the mild, simple, and rapid yet controlled and potentially also scalable methodology of TIRP with native proteins.

## Experimental

### Standard protocol for preparation of BSA polymer conjugates *via* TIRP

500 mg (7.69 µM) *Bovine Serum Albumin* was dissolved in 5 mL phosphate buffer (PBS 10 mM, 2.7 mM potassium chloride and 137 mM sodium chloride at pH 7.4) and deoxygenated with nitrogen for 20 min. Then, 1.3 mg (3.75 µmol) of diphenyl(2,4,6-trimethylbenzoyl) phosphine oxide (TPO) was dissolved in 0.1 mL dimethyl sulfoxide (DMSO) and slowly added to the protein solution, which was subsequently deoxygenated for another 20 min. 50 equivalents (375 µM) of the corresponding vinyl monomer was separately dissolved in PBS (50 mg mL^−1^) and 0.05 mol% (relative to the monomer, 0.18 µmol) of tris(2-phenylpyridine) iridium catalyst (Ir-Cat.) was dissolved in 0.1 mL DMSO and added before the whole solution was deoxygenated for 20 min. The protein/TPO solution was then irradiated for 3 min under the UV lamp (*λ* = 405 nm) at an intensity of 114 mW cm^−2^. After this, the monomer/Ir-Cat. mixture was added and irradiated for another 60 min at 5700 mW cm^−2^. The reaction mixture was then purified by dialysis (exclusion volume 50 kDa) and lyophilized.

### Standard protocol for preparation of CRL polymer conjugates *via* TIRP

200 mg (3.47 µM) *Candida rugosa lipase* was dissolved in 4 mL PBS buffer and deoxygenated with nitrogen for 20 min. Then, 0.6 mg (1.73 µmol) of TPO was dissolved in 0.2 mL DMSO and slowly added to the protein solution, which was subsequently deoxygenated for another 20 min. 50 or 200 equivalents, respectively (174 µM resp. 694.6 µM) of the corresponding vinyl monomer were separately dissolved in PBS (50 mg mL^−1^) and 0.05 mol% (relative to the monomer, 0.09 µmol resp. 0.35 µmol) of tris(2-phenylpyridine) iridium catalyst (Ir-Cat.) were dissolved in 0.2 mL DMSO and added before the whole solution was deoxygenated for 20 min. The protein/TPO solution was then irradiated for 10 min under the UV lamp (*λ* = 405 nm) at an intensity of 5700 mW cm^−2^. After this, the monomer/Ir-Cat. mixture was added and irradiated for another 60 min at 5700 mW cm^−2^. The reaction mixture was then purified by dialysis (exclusion volume 50 kDa) and lyophilized.

### Pierce-protein-assay

Protein concentrations were determined using a Pierce 660 nm Protein assay. Therefore, 1 mg mL^−1^ stock solutions (PBS) of all protein polymer conjugates were prepared. Native BSA served as a standard to determine the calibration curve (2000 µg mL^−1^, 1000 µg mL^−1^, 500 µg mL^−1^, 250 µg mL^−1^, 125 µg mL^−1^, 62.5 µg mL^−1^, and 31.25 µg mL^−1^). All measurements were performed in 96-well plates (Greiner®, polystyrene, F-bottom, transparent, 382 µL total volume per well) as triplicates. In each case, 10 µL of the corresponding sample was presented and mixed with 150 µL of the dye solution. The plate was shaken at 20 °C for 1 min and then incubated for 5 min at the same temperature. Absorbance is measured at 660 nm using the microplate reader (CLARIOstar®) and based on the values the corresponding protein concentrations are adjusted.

### Ellman's assay

The Ellman's assay was performed according to the manufacturer's instructions. All thiol detections were performed in reaction buffer: 0.1 M sodium phosphate, pH 8.0, containing 1 mM EDTA. In addition, Ellman's stock solution of 4 mg mL^−1^ Ellman's reagent and a 0.75 mM protein stock solution were prepared in reactions buffer. The assay was performed in triplicate determination using the Microplate Reader. For this procedure, 196 µL of the buffer was mixed with 20 µL of the protein stock solution followed by 4 µL of the Ellman's stock solution and incubated for 15 min. The absorbance was measured at 412 nm and the amount of accessible thiols was determined based on a previously established calibration curve. This resulted in 54% free thiols for the native BSA, which was in agreement with previously reported data.^42,95^ For CRL, a value of 95% free thiol was detected, which corresponds to one thiol per protein.

### Turbidity assay

For the measurement of protein–protein clustering by specific ligand–receptor interactions in solution, absorbance spectra were measured as triplicates on a microplate reader. Specific ligand–receptor interactions in the protein–protein clusters were determined based on quantifying the turbidity of the solution. Absorbance spectra (*λ* = 420 nm) were measured as triplicates in lectin binding buffer (LBB, 50 mM sodium chloride, 1 mM manganese(ii) chloride tetrahydrate, 1 mM calcium chloride and 10 mM HEPES dissolved in ultrapure water at a pH of 7.4). Initially, 120 µL of the native BSA and each BSA–polymer conjugate (50 µM) were provided at the beginning. Then, 40 µL of the respective lectin (5 µM, ConA and RCA_120_) was added and the absorbance was measured after 5, 10, 20 and 40 min, respectively. Subsequently, 40 µL of the inhibitor (30 mM, αMeMan for ConA and galactose for RCA_120_) was added and absorbance was measured after 5, 10 and 20 min, respectively. For the purposes of reference, 40 µL of LBB were added to 120 µL of native BSA (50 µM) for the competition experiment, and an additional 40 µL of LBB were added for the inhibition experiment.

### Determination of the half-maximum turbidity

The half-maximal turbidity was determined from a concentration series measurement of the turbidity assay (performed in triplicates). A 5 µM solution of ConA (in LBB buffer) was prepared and the corresponding amount of ligand solution (in LBB) was added, resulting in the final concentrations to be measured: for BSA–p(ManEAA) (*3*): 0 µM, 0.125 µM, 0.25 µM, 0.5 µM, 0.75 µM, 1 µM, 1.5 µM, 2 µM, 2.5 µM, 5 µM, 10 µM, 15 µM, 20 µM and for p(ManEAA) (*S7*): 0.75 µM, 1 µM, 1.5 µM, 2 µM, 2.25 µM, 2.5 µM, 2.75 µM, 3 µM, 3.25 µM, 3.5 µM, 4 µM, 4.5 µM, 5 µM. After adding the ligand and incubating for 20 minutes, the absorbance was measured at 420 nm. The absolute values of the absorbance were corrected according to the reference (only ConA in LBB) and normalized between zero and one. Each of the triplicate measurements was individually plotted logarithmically against the concentration and fitted *via* the Hil1. The mean value and the standard deviation of the corresponding concentration at 50% absorption were determined as the value of the half-maximum turbidity.

### Competition-inhibition assay on glass surface of protein–polymer conjugates

For the fluorescence images, a half-sided dip coating approach was used, in which first one half of the glass surface is dipped in the solution containing BSA or a BSA–polymer conjugate followed by coating of the entire surface with a different BSA polymer conjugate (1 mg mL^−1^ in LBB buffer). For this approach, ibidi® µ-Slide 18-Well Glass Bottom plates were used. Initially, the glass surface was pre-cleaned and activated in the Ozone Cleaner for 30 min. Then, 20 µL of the first sample was incubated on the slanted glass surface (ESI Fig. S61A†) for 30 min at 100 rpm. Afterwards the coated side was washed five times with 20 µL LBB and subsequently the entire chamber was incubated with 80 µL of a different sample for 30 min at 100 rpm in a balanced state. Afterwards, the entire chamber was washed five times with LBB and covered with 90 µL of LBB. To this solution, 10 µL of the appropriate lectin (5 µM) was added and incubated for 30 min at 100 rpm before the surface was washed five times with LBB to remove the excess lectin. For the fluorescence spectroscopy measurements, the chambers were filled with 80 µL of LBB. To perform the lectin release studies, 45 µL of LBB were mixed with 45 µL of the corresponding inhibitor polymer *S7*, *S8* (15 mM) and added to the chamber. After incubation for one hour at 100 rpm the surface was washed five times with LBB.

### Activity measurements of CRL–polymer conjugates

Activity measurements of CRL-based samples were performed in NaH_2_PO_4_ buffer (100 mM, pH 7.3) in an adapted protocol from Margesin *et al.*^83^ Additionally, a 100 mM 4-nitrophenyl acetate (pNPA) stock solution was prepared in isopropanol. The different CRL–polymer conjugates as well as the native CRL were freshly prepared as 17 µM solution in NaH_2_PO_4_ buffer and vortexed to give a suspension.

#### Assay I

1 mL of conjugates *5*, *6*, and *7* were gently heated in a water bath to 30 °C for 10 min before adding 1 µL of the pNPA stock solution and incubating at 30 °C for additional 10 min. Samples were then cooled to 0 °C in an ice bath for 10 min and centrifuged at, 2000 rpm (2 °C) for 5 min before triplicate absorbance measurements were performed for each sample.

#### Assay II

133 µL of a 5 µM RCA_120_ solution in LBB was assed to 400 µL of the 17 µM conjugate solutions (*6* and *7*) and incubated for 20 min. Native CRL sample was supplemented with 133 µL of NaH_2_PO_4_ buffer instead of a lectin to obtain equal dilutions. Subsequently, all samples were heated in a water bath to 30 °C for 10 min before adding 1 µL of the pNPA stock solution and incubating for additional 10 min at 30 °C. Samples were then cooled to 0 °C in an ice bath for 10 min and centrifuged at 2000 rpm (2 °C) for 5 min before triplicate measurements were performed for each sample. Activity values represent the measured absorption maxima of the hydrolase cleavage product, *p*-nitrophenol at 400 nm. All absorption values were normalized between zero and one.

### Thermal amino acid degradation

50 mg of the corresponding conjugate was dissolved in 6 M HCl, boiled for six hours at 110 °C under reflux, neutralized with NaOH and the polymer was isolated *via* dialysis (exclusion volume: 1 kDa) and freeze-dried.

## Author contributions

M. F.: investigation (lead), methodology (lead), validation, writing – original draft preparation. S. S.: investigation (supporting). L. B.: investigation (supporting). S. B.: investigation (supporting), methodology (supporting). A. B.: conceptualization (supporting). U. G.-D.: investigation, methodology (supporting), validation, supervision (equal), writing – review & editing (equal). L. H.: conceptualization (lead), funding acquisition, methodology (supporting), project administration, supervision (equal), writing – review & editing (equal).

## Conflicts of interest

There are no conflicts to declare.

## Supplementary Material

SC-015-D4SC04818K-s001

## Data Availability

The data supporting this article have been included as part of the ESI.†

## References

[cit1] Lele B. S., Murata H., Matyjaszewski K., Russell A. J. (2005). Biomacromolecules.

[cit2] Fu C. Y., Wang Z. G., Gao Y. T., Zhao J., Liu Y. C., Zhou X. Y., Qin R. R., Pang Y. Y., Hu B. W., Zhang Y. Y., Nan S. P., Zhang J. R., Zhang X., Yang P. (2023). Nat. Sustain..

[cit3] Wright T. A., Page R. C., Konkolewicz D. (2019). Polym. Chem..

[cit4] Wang Y. J., Wu C. (2018). Biomacromolecules.

[cit5] Mancini R. J., Lee J., Maynard H. D. (2012). J. Am. Chem. Soc..

[cit6] Cummings C., Murata H., Koepsel R., Russell A. J. (2014). Biomacromolecules.

[cit7] DeBenedictis E. P., Hamed E., Keten S. (2016). ACS Nano.

[cit8] Baker S. L., Munasinghe A., Kaupbayeva B., Kang N. R., Certiat M., Murata H., Matyjaszewski K., Lin P., Colina C. M., Russell A. J. (2019). Nat. Commun..

[cit9] Abuchowski A., van Es T., Palczuk N. C., Davis F. F. (1977). J. Biol. Chem..

[cit10] Hou Y. Q., Lu H. (2019). Bioconjugate Chem..

[cit11] Duncan R. (2003). Nat. Rev. Drug Discovery.

[cit12] Welch R. P., Lee H., Luzuriaga M. A., Brohlin O. R., Gassensmith J. J. (2018). Bioconjugate Chem..

[cit13] Theodorou A., Liarou E., Haddleton D. M., Stavrakaki I. G., Skordalidis P., Whitfield R., Anastasaki A., Velonia K. (2020). Nat. Commun..

[cit14] Pelegri-O'Day E. M., Lin E. W., Maynard H. D. (2014). J. Am. Chem. Soc..

[cit15] Liu X., Gao W. (2021). Angew Chem. Int. Ed. Engl..

[cit16] Ko J. H., Maynard H. D. (2018). Chem. Soc. Rev..

[cit17] Zhang P., Sun F., Liu S. J., Jiang S. Y. (2016). J. Controlled Release.

[cit18] Liang S., Liu Y., Jin X., Liu G., Wen J., Zhang L. L., Li J., Yuan X. B., Chen I. S. Y., Chen W., Wang H., Shi L. Q., Zhu X. Y., Lu Y. F. (2016). Nano Res..

[cit19] Heredia K. L., Maynard H. D. (2007). Org. Biomol. Chem..

[cit20] Wu Y., Ng D. Y., Kuan S. L., Weil T. (2015). Biomater. Sci..

[cit21] Lawrence P. B., Price J. L. (2016). Curr. Opin. Chem. Biol..

[cit22] Rondon A., Mahri S., Morales‐Yanez F., Dumoulin M., Vanbever R. (2021). Adv. Funct. Mater..

[cit23] Harris J. M., Chess R. B. (2003). Nat. Rev. Drug Discovery.

[cit24] Li C., Li T., Tian X., An W., Wang Z., Han B., Tao H., Wang J., Wang X. (2024). Front. Pharmacol.

[cit25] Ahmed M., Wattanaarsakit P., Narain R. (2013). Eur. Polym. J..

[cit26] Sun J., Guo J., Zhang L., Gong L., Sun Y., Deng X., Gao W. (2023). J. Controlled Release.

[cit27] Cui Y., Li Z., Wang L., Liu F., Yuan Y., Wang H., Xue L., Pan J., Chen G., Chen H. (2016). J. Mater. Chem. B.

[cit28] Lipinski T., Kitov P. I., Szpacenko A., Paszkiewicz E., Bundle D. R. (2011). Bioconjugate Chem..

[cit29] Geng J., Mantovani G., Tao L., Nicolas J., Chen G., Wallis R., Mitchell D. A., Johnson B. R., Evans S. D., Haddleton D. M. (2007). J. Am. Chem. Soc..

[cit30] Zhang H., Weingart J., Gruzdys V., Sun X.-L. (2016). ACS Macro Lett..

[cit31] Miura Y. , in Comprehensive Glycoscience, Elsevier, 2nd edn, 2021, pp. 250–262

[cit32] Zhao W. G., Liu F., Chen Y., Bai J., Gao W. P. (2015). Polymer.

[cit33] Messina M. S., Messina K. M. M., Bhattacharya A., Montgomery H. R., Maynard H. D. (2020). Prog. Polym. Sci..

[cit34] Miura Y. (2020). J. Mater. Chem. B.

[cit35] Sumerlin B. S. (2012). ACS Macro Lett..

[cit36] Mansfield K. M., Maynard H. D. (2018). ACS Macro Lett..

[cit37] Zhang Q., Li M., Zhu C., Nurumbetov G., Li Z., Wilson P., Kempe K., Haddleton D. M. (2015). J. Am. Chem. Soc..

[cit38] Averick S., Simakova A., Park S., Konkolewicz D., Magenau A. J., Mehl R. A., Matyjaszewski K. (2012). ACS Macro Lett..

[cit39] Szczepaniak G., Łagodzińska M., Dadashi-Silab S., Gorczyński A., Matyjaszewski K. (2020). Chem. Sci..

[cit40] Olson R. A., Levi J. S., Scheutz G. M., Lessard J. J., Figg C. A., Kamat M. N., Basso K. B., Sumerlin B. S. (2021). Macromolecules.

[cit41] Tucker B. S., Coughlin M. L., Figg C. A., Sumerlin B. S. (2017). ACS Macro Lett..

[cit42] Heredia K. L., Bontempo D., Ly T., Byers J. T., Halstenberg S., Maynard H. D. (2005). J. Am. Chem. Soc..

[cit43] Braunecker W. A., Matyjaszewski K. (2008). Prog. Polym. Sci..

[cit44] Wei H., Pahang J. A., Pun S. H. (2013). Biomacromolecules.

[cit45] Gerland B., Goudot A., Pourceau G., Meyer A., Vidal S., Souteyrand E., Vasseur J. J., Chevolot Y., Morvan F. (2012). J. Org. Chem..

[cit46] Matyjaszewski K. (2012). Macromolecules.

[cit47] Nicolas J., San Miguel V., Mantovani G., Haddleton D. M. (2006). Chem. Commun..

[cit48] Theodorou A., Gounaris D., Voutyritsa E., Andrikopoulos N., Baltzaki C. I. M., Anastasaki A., Velonia K. (2022). Biomacromolecules.

[cit49] Barner L., Barner-Kowollik C., Davis T. P., Stenzel M. H. (2004). Aust. J. Chem..

[cit50] De P., Li M., Gondi S. R., Sumerlin B. S. (2008). J. Am. Chem. Soc..

[cit51] Boyer C., Bulmus V., Liu J. Q., Davis T. P., Stenzel M. H., Barner-Kowollik C. (2007). J. Am. Chem. Soc..

[cit52] Huang Y., Li X., Zhang Y. C., Shi Z. W., Zeng L., Xie J. B., Du Y. C., Lu D., Hu Z. G., Cai T., Luo Z. T. (2021). ACS Appl. Mater. Interfaces.

[cit53] Spears B. R., Waksal J., McQuade C., Lanier L., Harth E. (2013). Chem. Commun..

[cit54] Ciepluch K., Radulescu A., Hoffmann I., Raba A., Allgaier J., Richter D., Biehl R. (2018). Bioconjugate Chem..

[cit55] Pang Y., Liu J. Y., Qi Y. Z., Li X. H., Chilkoti A. (2016). Angew. Chem., Int. Ed..

[cit56] Kaupbayeva B., Boye S., Munasinghe A., Murata H., Matyjaszewski K., Lederer A., Colina C. M., Russell A. J. (2021). Bioconjugate Chem..

[cit57] Kaupbayeva B., Murata H., Rule G. S., Matyjaszewski K., Russell A. J. (2022). Biomacromolecules.

[cit58] Bonda L., Valles D. J., Wigger T. L., Meisner J., Braunschweig A. B., Hartmann L. (2023). Macromolecules.

[cit59] Peters T. (1985). Adv. Protein Chem..

[cit60] Zhang J. J., Xiao S. W., Shen M. X., Sun L., Chen F., Fan P., Zhong M. Q., Yang J. T. (2016). RSC Adv..

[cit61] Zhao C., Chen Q., Patel K., Li L. Y., Li X. S., Wang Q. M., Zhang G., Zheng J. (2012). Soft Matter.

[cit62] Wu H. X., Tan L., Tang Z. W., Yang M. Y., Xiao J. Y., Liu C. J., Zhuo R. X. (2015). ACS Appl. Mater. Interfaces.

[cit63] Trzebicka B., Szweda R., Kosowski D., Szweda D., Otulakowski L., Haladjova E., Dworak A. (2017). Prog. Polym. Sci..

[cit64] Stayton P. S., Shimoboji T., Long C., Chilkoti A., Chen G., Harris J. M., Hoffman A. S. (1995). Nature.

[cit65] Jalababu R., Veni S. S., Reddy K. V. N. S. (2018). J. Drug Delivery Sci. Technol..

[cit66] Galaev I. Y., Mattiasson B. (1999). Trends Biotechnol..

[cit67] Miura Y., Hoshino Y., Seto H. (2016). Chem. Rev..

[cit68] Grochulski P., Li Y., Schrag J. D., Bouthillier F., Smith P., Harrison D., Rubin B., Cygler M. (1993). J. Biol. Chem..

[cit69] Hoyt E. A., Cal P. M., Oliveira B. L., Bernardes G. J. (2019). Nat. Rev. Chem.

[cit70] Hannig G., Makrides S. C. (1998). Trends Biotechnol..

[cit71] Schütz A., Bernhard F., Berrow N., Buyel J. F., Ferreira-da-Silva F., Haustraete J., Van den Heuvel J., Hoffmann J.-E., De Marco A., Peleg Y. (2023). STAR Protoc..

[cit72] Pabbathi A., Patra S., Samanta A. (2013). ChemPhysChem.

[cit73] Kaupbayeva B., Murata H., Matyjaszewski K., Russell A. J., Boye S., Lederer A. (2021). Chem. Sci..

[cit74] Michael W., Stephan H., Anja T., Alfred F. (2014). Anal. Chem..

[cit75] Bontempo D., Heredia K. L., Fish B. A., Maynard H. D. (2004). J. Am. Chem. Soc..

[cit76] Hermanson G. T. , Bioconjugate Techniques, Academic Press, 2013

[cit77] Greenfield N. J. (2006). Nat. Protoc..

[cit78] Riccardi C. M., Cole K. S., Benson K. R., Ward J. R., Bassett K. M., Zhang Y., Zore O. V., Stromer B., Kasi R. M., Kumar C. V. (2014). Bioconjugate Chem..

[cit79] Zhang T., An W., Sun J., Duan F., Shao Z., Zhang F., Jiang T., Deng X., Boyer C., Gao W. (2022). Nano Lett..

[cit80] Benjamin S., Pandey A. (1998). Yeast.

[cit81] Mala J. G., Takeuchi S. (2008). Anal. Chem. Insights.

[cit82] Vanleeuw E., Winderickx S., Thevissen K., Lagrain B., Dusselier M., Cammue B. P. A., Sels B. F. (2019). ACS Sustainable Chem. Eng..

[cit83] Margesin R., Feller G., Hammerle M., Stegner U., Schinner F. (2002). Biotechnol. Lett..

[cit84] Hou X. Y., Shi Q. H. (2023). Chin. J. Chem. Eng..

[cit85] Lisowska E. (2002). Cell. Mol. Life Sci..

[cit86] Voit B., Appelhans D. (2010). Macromol. Chem. Phys..

[cit87] Gerling-Driessen U. I. M., Hoffmann M., Schmidt S., Snyder N. L., Hartmann L. (2023). Chem. Soc. Rev..

[cit88] Gerke C., Ebbesen M. F., Jansen D., Boden S., Freichel T., Hartmann L. (2017). Biomacromolecules.

[cit89] Gestwicki J. E., Cairo C. W., Strong L. E., Oetjen K. A., Kiessling L. L. (2002). J. Am. Chem. Soc..

[cit90] Valles D. J., Zholdassov Y. S., Korpanty J., Uddin S., Naeem Y., Mootoo D. R., Gianneschi N. C., Braunschweig A. B. (2021). Angew. Chem..

[cit91] Li W., Wang H., Zhao Z., Gao H., Liu C., Zhu L., Wang C., Yang Y. (2019). Adv. Mater..

[cit92] Schneider C., Smith D. F., Cummings R. D., Boligan K. F., Hamilton R. G., Bochner B. S., Miescher S., Simon H.-U., Pashov A., Vassilev T. (2015). Sci. Transl. Med..

[cit93] Rillahan C. D., Paulson J. C. (2011). Annu. Rev. Biochem..

[cit94] Jacobi F., de la Calle A. C., Boden S., Grafmüller A., Hartmann L., Schmidt S. (2018). Biomacromolecules.

[cit95] Janatova J., Fuller J. K., Hunter M. J. (1968). J. Biol. Chem..

