# Supplementary material for: Thiol-selective native grafting from polymerization for the generation of protein–polymer conjugates
Source: Chem Sci. 2024 Sep 18;15(40):16768–77. doi: 10.1039/d4sc04818k (PMC11418805; doi:10.1039/d4sc04818k)
Supplement: SC-015-D4SC04818K-s001 [file SC-015-D4SC04818K-s001.pdf]

Supplementary Information (SI) for Chemical Science.
This journal is © The Royal Society of Chemistry 2024

Supporting Information

Thiol-selective *native grafting-from* polymerization for the generation of protein-polymer conjugates

Melina I. Feldhof,[a] Sandro Sperzel,[a] Lorand Bonda,[a] Susanne Boye,[b] Adam B. Braunschweig,[c] Ulla I.M. Gerling-Driessen,*[d] and Laura Hartmann*[d]

[a] Department of Organic and Macromolecular Chemistry, Heinrich-Heine-University Düsseldorf, Universitätsstraße 1, 40225 Düsseldorf, Germany
[b] Zentrum Makromolekulare Strukturanalyse, Leibniz-Institut für Polymerforschung Dresden, Hohe Str. 6, 01069 Dresden, Germany
[c] Advanced Science Research Center, Graduate Center, City University of New York, 85 St. Nicholas Terrace, New York, NY 10031, USA; PhD Programs in Chemistry and Biochemistry, Graduate Center, City University of New York, 365 5th Avenue, New York, NY 10016, USA; Department of Chemistry, Hunter College, 695 Park Avenue, New York, NY 10065, USA
[d] Institute for Macromolecular Chemistry, University of Freiburg, Stefan-Meier-Str. 31, D-79104 Freiburg i.Br., Germany, E-mail: laura.hartmann@hhu.de, ulla.gerling-driessen@makro.uni-freiburg.de

*Corresponding author

Table of Contents

1. Experimental Parts 2
1.1 Materials 2
1.2 Instrumentation 2
1.3 General Methods 4
2. Synthesis and analytical data 6
2.1 Synthesis of carbohydrate monomers ***S1*-*S4*** 6
2.2 Synthesis and measurements of BSA-polymer conjugates **1-4** 16
2.3 Synthesis and measurements of CRL-polymer conjugates **5-7** 40
2.4 Synthesis of inhibitor polymers ***S7*** and ***S8*** 46
2.5 Carbohydrate-lectin binding studies on glass surfaces 49

1. Experimental Parts

1.1 Materials

All chemicals and solvents were used without further purification and were purchased from commercial sources. Type I water, purified by Barnstead™ MicroPure™ ThermoFisher SCIENTIFIC ultrapure water system, was used unless otherwise mentioned. *Bio-Rad:* 10xtris-glycin/SDS-running buffer, pH 8.6, coomassie brilliant blue, precision plus protein™ kaleidoscope™; *Acros Organics:* 4-nitrophenyl acetate ≥ 97%, N-isopropylacrylamide ≥ 99%, para- toluene sulfonic acid ≥ 99%; *Alfa Aesar*: boron trifluoride dietherate ≥ 98%, sodium methanolate ≥ 98%; *BIOZOL*: RCA_{120}, RCA_{120} (FITC); *BLDpharm*: β-D-galactose pentaacetate ≥ 98%; *Carl Roth*: guanidine hydrochloride ≥ 99.5%; *Eurisotope*: chloroform-d ≥ 99.8%; *Fisher BioReagents™*: 2-(4-(2-hydroxyethyl)-1-piperazinyl)-ethane sulfonic acid (HEPES) ≥ 99%; *Fisher scientific*: Concanavalin A Alexa 647; *Fluka*: α-D-mannose ≥ 99; *Grüssing*: sodium dihydrogen phosphates ≥ 99%, sodium bicarbonate ≥ 99.5%; *Honeywell*: sodium sulphate ≥ 99%, acetonitrile ≥ 99.9%; *J&K*: tris(2-phenylpyridine)iridium(III) ≥ 99%; *Merck*: calcium chloride ≥ 90%, deuterated water ≥ 99.9%, dichloromethane ≥ 99.8%, acetic anhydride ≥ 99.5%, methanol ≥ 99.8%, sodium carbonate ≥ 99.7%, sodium chloride 100%; *PanReac AppliChem*: phosphate buffered saline tablet (PBS), dimethyl sulfoxide (DMSO) ≥ 99.9%; *Sigma Aldrich*: Concanavalin A (ConA), N-hydroxyethyl acrylamide ≥ 97.4%, 5,5′-Dithiobis(2-nitrobenzoic acid) ≥ 98%, Amberlite®IR120, *bovine serum albumin* (BSA) ≥ 96%, *candida rugosa lipase* ≥ 2U/mg, DMSO-d_6 ≥ 99.9%, ethylenediaminetetraacetic acid 99%, manganese(II) chloride tetrahydrate ≥ 99%; *TCI*: diphenyl(2,4,6-trimethylbenzoyl)phosphine oxide (TPO) ≥ 98%, tris(2-carboxyethyl)phosphine (TCEP) ≥ 98%; *Thermo-Fisher*: pierce BCA protein assay kit; *VWRChemicals*: acetone 100%.

1.2 Instrumentation

Nuclear magnetic Resonance Spectroscopy (NMR):

^{1}H-NMR spectra were measured with a Bruker Avance III 600 (600 MHz) at room temperature. Chemical shifts were reported in delta (δ) expressed in parts per million (ppm) for all ^{1}H-NMR spectrum. D_2O serves as the deuterated solvents for all ^{1}H-NMR spectrum. Parts of non-deuterated solvent were used as an internal standard with δ = 4.79 for HDO. Multiplicities were abbreviated as the following: singlet (s), doublet (d), triplet (t), quartet (q), multiplet (m). The assignment of carbohydrate signals on NMR followed the chronological enumeration of protons/carbon atoms within a monosaccharide starting from the reducing end.

Reversed Phase High Pressure Liquid Chromatography Mass Spectrometry (RP-HPLC-MS): RP-HPLC-MS measurements were performed on Agilent Technologies 1260 Infinity series coupled with Agilent quadrupole mass spectrometer with an Electrospray Ionization (ESI) source operating in a m/z range of 200 to 2000. All spectra were measured with A: 95% H_2O, 5% ACN, 0.1% formic acid and B: 5% H_2O, 95% ACN, 0.1% formic acid. Separation was performed at 25 °C using an Agilent MZ-Aqua Perfect C_{18} 3 µm (50 x 3.0 mm) column with a linear solvent gradient starting at 100% A, ending at 50% B in 17 min at a flow rate of 0.4 mL/min. Indicated purities were determined by integration of the UV-signal detected by a wavelength detector set to 214 nm with the OpenLab ChemStation software for LC/MS from Agilent Technologies.

UV/Vis spectroscopy: UV/Vis spectroscopy was performed with a dual-trace spectrometer Specord® 210 Plus from Analytik Jena AG. The measurements were conducted in quartz glass cuvettes (Starna GmbH d = 1 cm) at 20 °C. The instrument was operated with Win ASPECT PLUS software.

UV-Irradiation: UV irradiation of the polymerization was conducted with a UV-LED spot with a wavelength of 405 nm and a maximal irradiance of 114-5700 mW/cm^2 operated by a LEDControl both purchased from Opsytec Dr. Gröbel.

Microplate Reader: Absorption measurements were conducted on CLARIOstar® from BMG LABTECH. Measurements were performed at room temperature and analyzed by using BMG Mars software. 96 F-bottom corrugated plates from Greiner BIO-ONE were used for all measurements.

Sodium Dodecyl Sulfate – PolyAcrylamid Gel Electrophoresis (SDS-PAGE): Separations were performed with a 10% acrylamide gel at a voltage of 200 mV and current of 200 mA for about 36 min. 10xTris glycine/SDS buffer, which was diluted down to 1x, serves as the running buffer. Coomassie Brilliant Blue was used to stain the protein bands.

Asymmetrical flow field-flow fractionation with light scattering detection (AF4-LS): AF4-LS measurements were conducted on Eclipse Neon (Wyatt Technologies Europe) equipped with a Dilution Control Module™ (DCM) using an Agilent pump system (1260, Infinity Series) at 25 °C in PBS buffer (1 mM, pH 7.4). A short channel with fixed height (350 µm) and regenerated cellulose (cut-off: 10 kDa) as ultrafiltration membrane for all measurements. Detection was performed using the LS detector (DAWN Neon, Wyatt λ = 660 nm) with QELS option, RI detector (Optilab T-rEX, Wyatt) and UV detectors of various wavelengths (λ = 280, 300, 310, 330 nm).

Circular dichroism (CD): CD measurements were conducted on a Jasco J-710 CD spectrometer from Jasco Deutschland GmbH with quartz glass cuvettes (Starna GmbH d = 1 cm) at 20 °C in PBS buffer (pH 7.4).

Infrared spectroscopy (IR): IR spectroscopic measurements were conducted on a Nicolet iS50 FT-IR from Thermo Scientific with. The analysis was carried out with the OMNIC spectroscopy software from thermoscientific.

Fluorescence microscopy: All fluorescence microscopy images were conducted on Olympus IX73 with 60x oil-lenses. For the appropriate excitation we used Thorlabs LED-lamps (M625L4 – 625 nm, 700 mW, 1000 mA and M470L5 - 470 nm, 809 mW, 1000 mA).

Aqueous SEC-MALS-RI: SEC-MALS-RI measurements of the polymeric inhibitors ***S7*** and ***S8*** were conducted using Wyatt systems, isocratic pump, degasser, AS of 1260 Infinity series, LS detector: DAWN Heleos-II (Wyatt), λ = 660 nm RI detector: Optilab T-rEX (Wyatt), λ = 660 nm UV detector: UV detector SPD-M20 (Shimadzu): With variable wavelengths Channel (1) λ = 220 nm, Channel (2) λ = 230 nm Channel (3) λ = 250 nm, Channel (4) λ = 290 nm, Flow rate: 1 mL/min, temperature: 25 °C, Concentration: 3-4 mg/mL, Injection volume: 100 µL. Precolumn (50 mm, 2 x 160 Å of 300 mm and 1000 Å of 300 mm), two main columns (GE Healthcare GPC column Superdex 75 10/300, 8 mm diameter and 5 µm particle size), eluent: MilliQ water:acetonitrile 7:3 (v/v), 50 mM, NaH_2PO_4, 150 mM NaCl and 250 ppm NaN_3, pH = 7.0.

Differential Scanning Calorimetry (DSC): DSC measurements were performed using a Mettler Toledo DSC3 instrument calibrated with indium and zinc. Samples were weighed in 40 µL aluminum crucibles with perforated lids and experiments were performed under nitrogen atmosphere. Three measurements (heating, cooling, heating) were measured in the range of 20 °C - 200 °C with a heating or cooling rate of 10 K/min. DSC measurements were evaluated using STARe software.

1.3 General Methods

SDS-PAGE: Molecular weights of the protein polymer conjugates were determined using 10% acrylamide gels and 1X-Tris/glycine/SDS running buffer. 1 mg/mL stock solutions (in 1x PBS, pH 7.4) were prepared for each sample and 15 µL of each was mixed with 5 µL of Laemmli buffer followed by denaturation in a thermal shaker at 95 °C for 15 min. After cooling to room temperature, 8 µL of each sample mixture was applied to the gel. Precision Plus Protein™ Dual

Xtra Prestained Protein Standard (3µL) was used as reference for molecular weights. Separation was performed over a period of 36 min at 200 mV and 200 mA. Coomassie Brilliant Blue was used to stain the proteins. Stained gels were imaged using a ChemiDoc™ MP Imaging System gel from BioRad.

CD Measurements: CD measurements were conducted in quartz cuvettes (d = 1 cm). The measurement volume was 200 µL and the wavelength range was 190 nm – 260 nm. Measurements were performed as triplicates at a scanning speed of 100 nm/min. For thermal denaturation experiments temperature-dependent measurements at 222 nm were performed in a temperature range from 20 °C to 95 °C with an increase of 2 °C/min.

AF4-LS measurements: The samples were dissolved in a 1 – 2 mg/mL stock solution. 10 mM PBS buffer at pH 7.4, containing 200 mg/L NaN_3 to prevent bacteria or algae contamination was used as eluent and solvent. Triplicate measurements were performed for each sample and 50 µL were injected for each measurement. The optimized flow profile for the separation of all samples is shown in Figure 1 of the manuscript. The following protocol was applied in order to separate the different populations of BSA: channel flow was set to 1.0 mL/min with a detector of 0.4 mL/min, focusing was performed with a focus flow of 3.0 mL/min for 6 min followed by an isocratic elution step with a cross flow (F_x) of 3 mL/min for 15 min. The last step proceeds without F_x (0 mL/min) for 20 min. Molar masses were calculated from the LS data applying a Zimm fit. Refractive index increments (dn/dc) were determined by manual injection of varied sample concentrations into above mentioned RI detector.

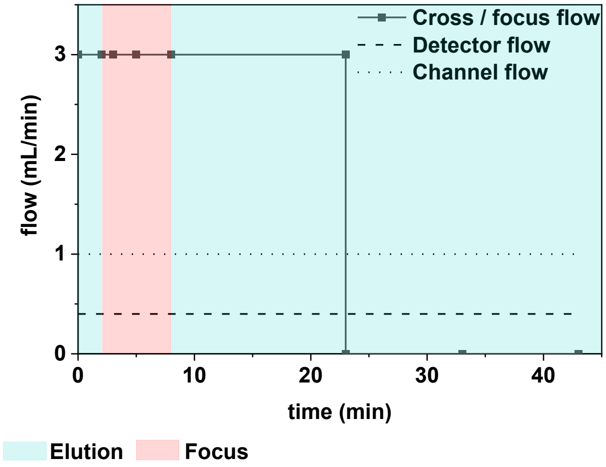

Fig. S1: Optimized AF4-LS separation flow profile.

2. Synthesis and analytical data

2.1 Synthesis of carbohydrate monomers *S1-S4*

The syntheses of the carbohydrate monomer are adapted from F. Schröer et al.[4]

S1 - 2,3,4,6-tetra-O-acetyl-α-D-mannopyranosyl-ethylacrylamide (AcManEAA)

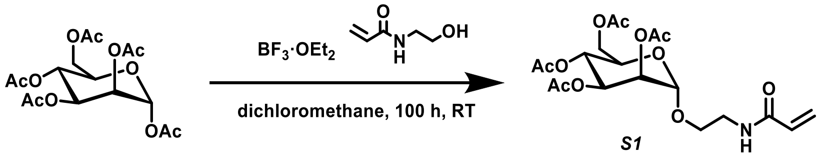

67.3 g (172.4 mmol) of α-D-mannosepentaacetate, was dissolved with 500 mL dichloromethane, 27.6 mL (266 mmol) of *N*-hydroxyethyl acrylamide was added, and the reaction solution was cooled to 0 °C for 10 min using an ice bath. Then 105 mL (828.6 mmol) of boron trifluoride diethyl etherate was added over a period of 30 min, and the reaction was stirred for 100 h at room temperature (20 °C) in the absence of light. The reaction progress was checked by DC (ethyl acetate/hexane, 1:1, *v/v*). The reaction solution was added to 1 L of ice water and stirred until the ice was completely melted. Then, the organic phase was washed four times with 400 mL of saturated sodium hydrogen carbonate solution and three times with 400 mL of water and then dried using sodium sulfate. The solvent was removed under reduced pressure. An amber viscous product was obtained with a yield of 64.55 g (84%).

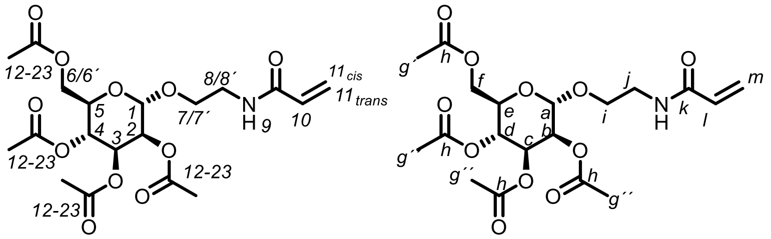

^{1}H-NMR (600 MHz, $CDCl_3$, 297.9 K) δ (ppm) 6.33 – 6.28 (dd, ^3J = 17.0, 1.4 Hz, 1H, *H10*), 6.18 – 6.13 (dd, ^3J = 17.0, 10.3 Hz, 1H, *$H11_{cis}$*), 6.11 (s, 1H, *H9*), 5.70 – 5.66 (dd, ^3J = 10.3, 1.4 Hz, 1H, *$H11_{trans}$*), 5.35 – 5.31 (m, 1H, *H4*), 5.27 – 5.23 (m, 2H, *H2 – H3*), 4.83 – 4.8 (d, ^3J = 1.7 Hz, 1H, *H1*), 4.26 – 4.22 (dd, ^3J = 12.2, 5.8 Hz, 1H, *H6a – H6b*), 4.13 – 4.10 (dd, ^3J = 12.2, 2.5 Hz, 1H, *H6a – H6b*), 3.99 – 3.95 (ddd, ^3J = 10.1, 5.7, 2.5 Hz, 1H, *H5*), 3.84 – 3.79 (m, 1H, *H7a - H7b*), 3.65 – 3.58 (m, 2H, *H7a, H7b, H8a, H8b*), 3.53 – 3.49 (m, 1H, *H8a - H8b*), 2.15 (s, 3H, *H12 - H23*), 2.09 (s, 3H, *H12 - H23*), 2.04 (s, 3H, *H12 - H23*), 2.00 (s, 3H, *H12 - H23*).

^{13}C-NMR (600 MHz, $CDCl_3$, 297.9 K) δ (ppm) 170.78 (4C, *Ch*), 165.73 (1C, *Ck*), 130.66 (1C, *Cl*), 127.11 (1C, *Cm*), 97.91 (1C, *Ca*), 69.49 (1C, *Ce*), 69.10, 68.91, 67.72 (3C, *Cb – Cd*), 66.29 (1C, *Ci*), 62.64 (1C, *Cf*), 39.27 (1C, *Cj*), 21.00 (3C, *C7g´´*), 20.83 (1C, *Cg´*).

LC-MS: m/z calculated for $C_{19}H_{27}NO_{11}$ $[M+H]^+$ 446.16, found 446.20 und $[M+Na]^+$ 468.16, found 468.20 determined relative purity: > 95%.

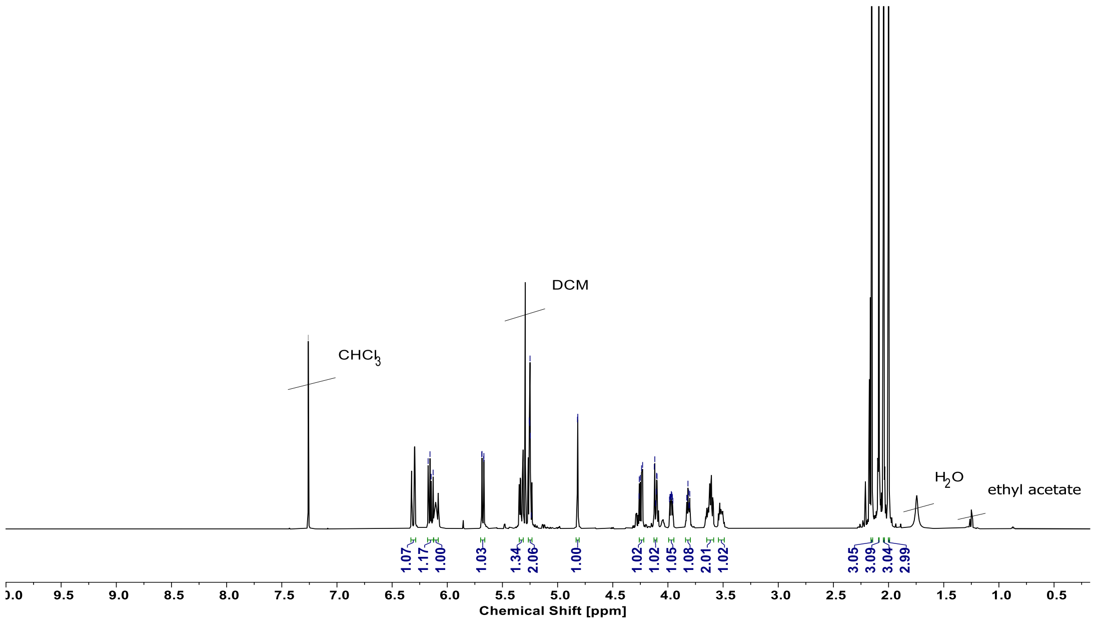

*Fig. S2: ^{1}H-NMR spectrum (600 MHz, CHCl3, 297.9 K) of α-D-ManAcEAA (**S1**).*

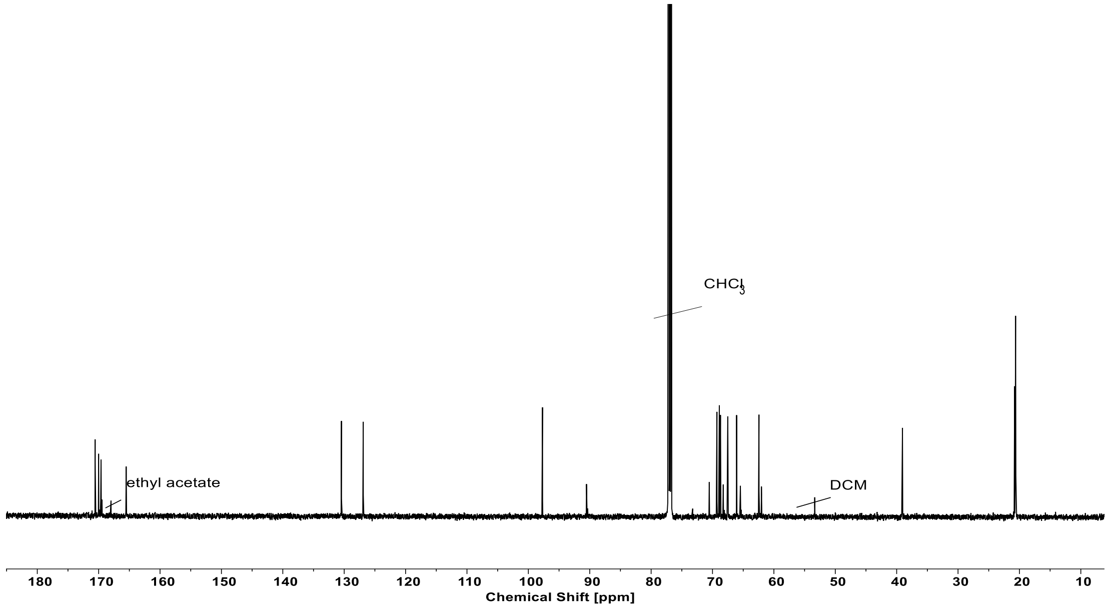

*Fig. S3: 13C-NMR spectrum (600 MHz, CHCl3, 297.9 K) of α-D-ManAcEAA (**S1**).*

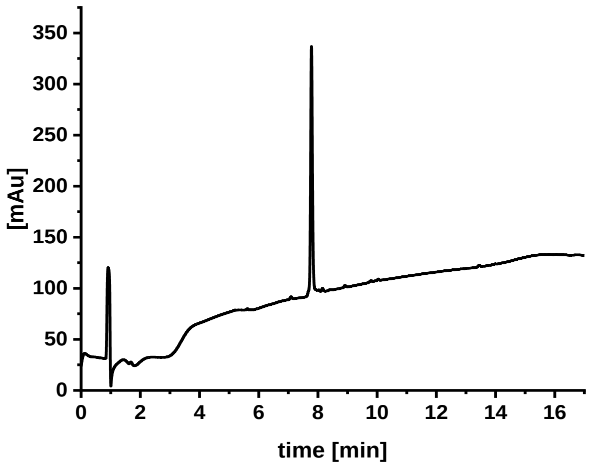

*Fig. S4: RP-HPCL chromatogram of α-D-ManAcEAA (**S1**) at t = 8.46 min (gradient of 5 to 95 vol% acetonitrile/water with 0.1 vol% formic acid, run time: 17 min).*

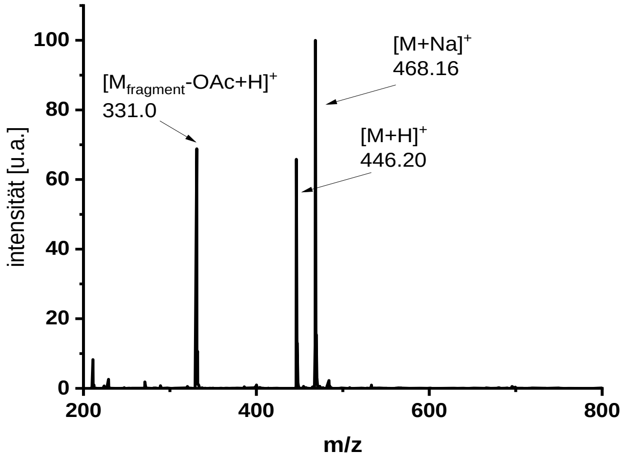

*Fig. S5: Mass spectrum of α-D-ManAcEAA (**S1**) at t = 8.46 min (gradient of 5 to 95 vol.% acetonitrile/water with 0.1 vol.% formic acid, run time: 17 min) by ESI-MS. The fragment at 331.0 m/z occurs during the ESI-MS measurement and corresponds to the product, where the acetyl group at the anomeric center was cleaved off.*

S2 - 2,3,4,6-Tetra-O-acetyl-β-D-Galactopyranosyl-ethylacrylamid (AcGalEAA)

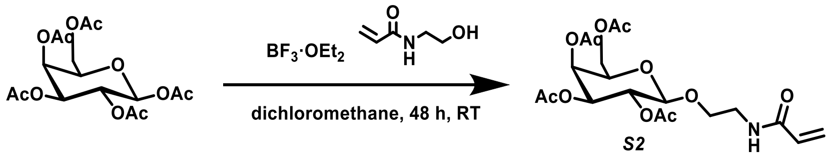

20 g (51.2 mmol) of β-D-galactose pentaacetate was dissolved with 200 mL of dichloromethane to which 8 mL (76.4 mmol) of *N*-hydroxyethyl acrylamide was added, and the reaction solution was cooled to 0°°C in an ice bath for 10 min. Subsequently, 31 mL (244.6 mmol) of boron trifluoride diethyl etherate was added over a period of 30 min and the reaction was stirred for 48 h at room temperature (20°°C) in the absence of light. The reaction progress was checked by DC (ethyl acetate/hexane 1:1, *v/v*). The reaction solution was added to 500 mL of ice water and stirred until the ice was completely melted. The organic phase was washed three times with 300 mL of each, saturated sodium bicarbonate solution and water and was subsequently dried using sodium sulfate. The solvent was removed under reduced pressure. A viscous semi-crystalline yellowish product was obtained with a yield of 27.49 g (> 95%).

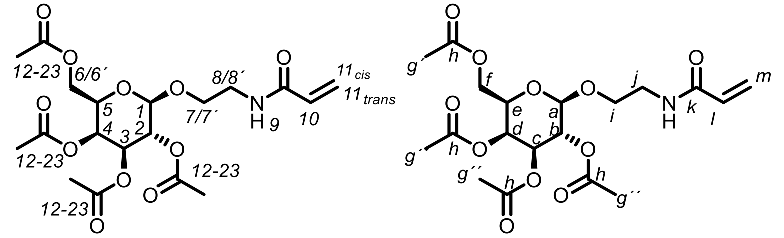

^{1}H-NMR (600 MHz, $CDCl_3$, 297.9 K) δ (ppm) 6.32 – 6.27 (dd, ^3J = 17.0, ^2J = 1.4 Hz, 1H, *$H11_{cis}$*), 6.13 – 6.08 (dd, ^3J = 17.0, 10.3 Hz, 1H, *H10*), 6.06 (s_{br}, 1H, *H9*), 5.67 – 5.64 (dd, ^3J = 10.3, ^2J = 1.4 Hz, 1H, *$H11_{trans}$*), 5.40 – 5.38 (dd, ^3J = 3.5, 1.1 Hz, 1H, *H4*), 5.20 – 5.16 (dd, ^3J = 10.5, 7.9 Hz, 1H, *H2 – H3*), 5.03 – 5.00 (dd, ^3J = 10.5, 3.4 Hz, 1H, *H2 – H3*), 4.48 – 4.45 (d, ^3J = 7.9 Hz, 1H, *H1*), 4.16 – 4.11 (m, 2H, *H6a – H6b*), 3.94 – 3.89 (m, 2H, *H7a – H7b*), 3.94 – 3.88 (ddd, ^3J = 10.3, 7.3, 3.4 Hz, 1H, *H5*), 3.64 – 3.58 (m, 1H, *H8a – H8b*), 3.52 – 3.46 (m, 1H, *H8a – H8b*), 2.16 (s, 3H, *H12 – H23*), 2.05 (s, 3H, *H12 – H23*), 2.04 (s, 3H, *H12 – H23*), 1.99 (s, 3H, *H12 – H23*).

^{13}C-NMR (600 MHz, $CDCl_3$, 297.9 K) δ (ppm) 170.53, 170.30, 170.20, 169.92 (4C*, Ch*), 165.59 (1C*, Ck*), 130.81 (1C, *Cl*), 126.86 (1C*, Cm*), 101.62 (1C, *Ca*), 71.02 (1C, *Ce*), 70.81*,* 69.27, 69.09 (3C, *Cb – Cd*), 67.10 (1C, *Ci*), 61.52 (1C, *Cf*)*,* 39.31 (1C, *Cj*)*,* 20.99*,* 20.81*,* 20.79 (3C, *Cg´´*)*,* 20.70 (1C, *Cg´*)*.*

LC-MS: m/z calculated for $C_{19}H_{27}NO_{11}$ $[M+H]^+$ 446.16, found 446.20, determined relative purity: 93%.

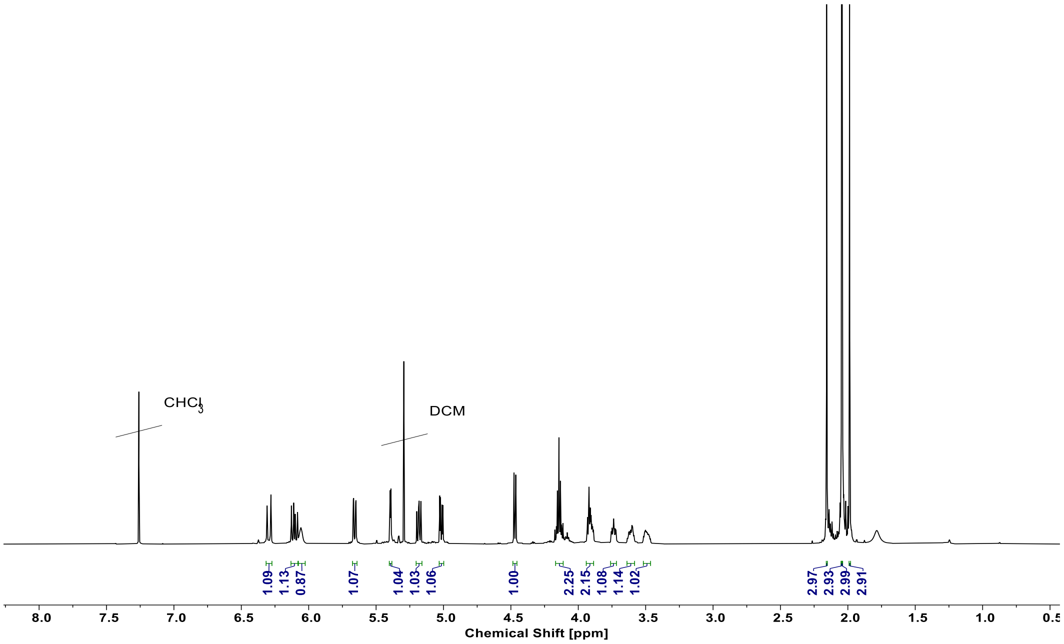

*Fig. S6: ^{1}H-NMR spectrum (600 MHz, CHCl3, 297.9 K) of β-D-GalAcEAA (**S2**).*

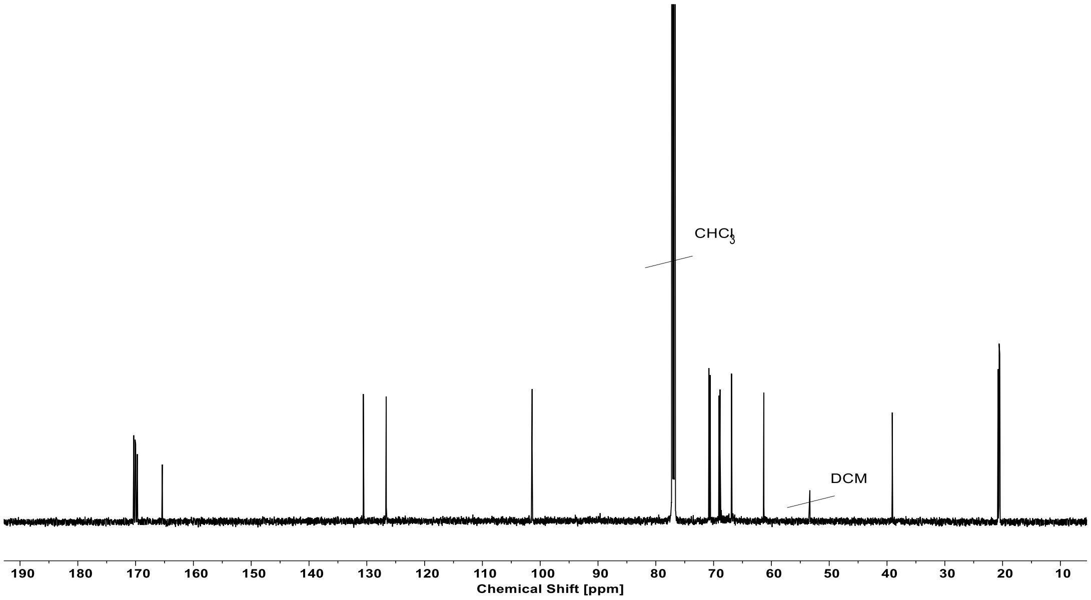

*Fig. S7: ^{13}C-NMR spectrum (600 MHz, $CHCl_3$, 297.9 K) of β-D-GalAcEAA (**S2**).*

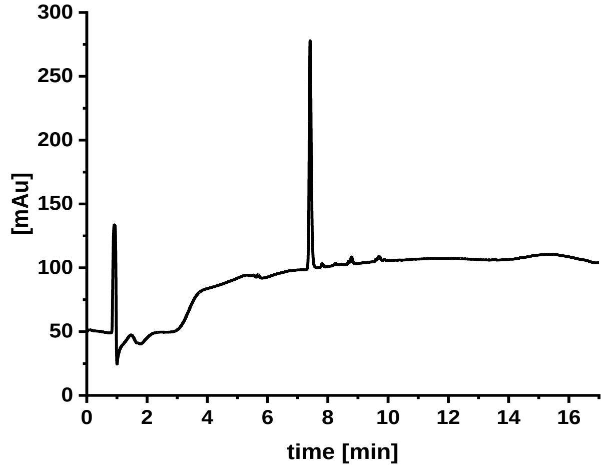

*Fig. S8: RP-HPCL chromatogram of β-D-GalAcEAA (**S2**) at t = 7.53 min (gradient of 5 to 95 vol% acetonitrile/water with 0.1 vol% formic acid, run time: 17 min).*

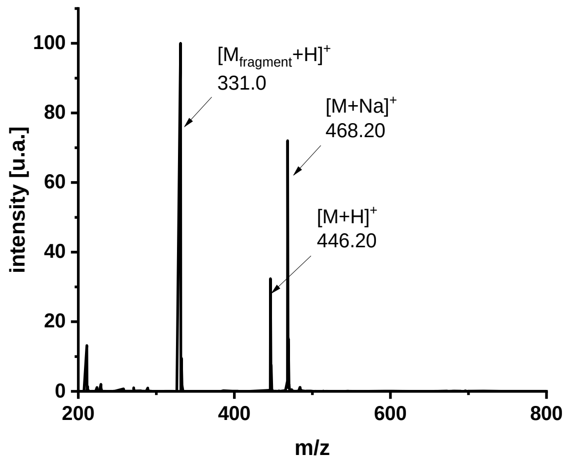

*Fig.S9: Mass spectrum of β-D-GalAcEAA (**S2**) at t = 7.53 min (gradient of 5 to 95 vol.% acetonitrile/water with 0.1 vol.% formic acid, run time: 17 min) by ESI-MS. The fragment at 331.0 m/z occurs during the ESI-MS measurement and corresponds to the product, where the acetyl group at the anomeric center was cleaved off.*

S3 – α-D-Mannopyranosyl-ethylacrylamid (ManEAA)

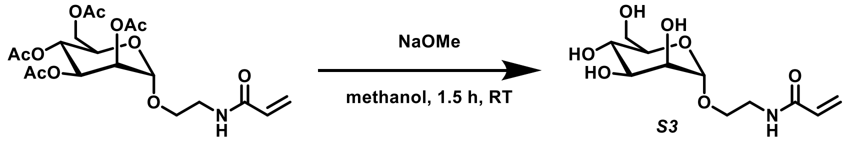

6.05 g (13.6 mmol) AcManEAA ***S1*** was dissolved in 50 mL methanol, mixed with 180 mL 0.2 M sodium methanolate/methanol solution and stirred for 90 min. The organic solvent was removed under reduced pressure and the residue dissolved in water. Amberlite®IR-120 was subsequently added as ion exchange resin until pH 7 was reached. The exchange resin was filtered off and the product lyophilized.

S3 was isolated as a yellowish oil with a yield of 4.62 g (> 95%).

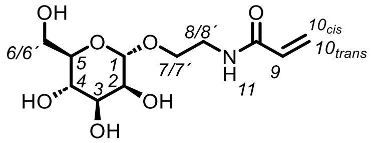

^{1}H-NMR (600 MHz, D_2O, 297.9 K) δ (ppm) 8.37 (s, 1H, *H11*), 6.24 – 6.15 (dd, ^3J = 17.1, 10.3 Hz, 1H, *H9*), 6.15 – 6.08 (dd, ^3J = 17.2, ^2J = 1.3 Hz, 1H, *H10*$_{cis}$), 5.71 – 5.66 (dd, ^3J = 10.3, ^2J = 1.3 Hz, 1H, *H10*$_{trans}$), 4.80 – 4.77 (s, 1H, *H1*), 4.92 – 3.27 (m, H11, *H2 – H5, H6a – H6b*, *H7a – H7b*, *H8a – H8b*).

LC-MS: m/z calculated for $C_{11}H_{19}NO_7$ $[M+Na]^+$ 300.12 found 300.00 in injection peak.

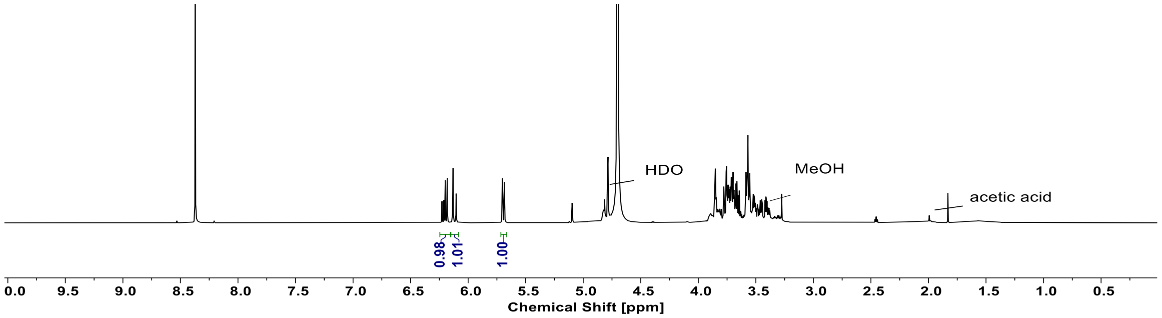

*Fig. S10: ^{1}H-NMR spectrum (600 MHz, D_2O, 297.9 K) of α-D-ManEAA (**S3**).*

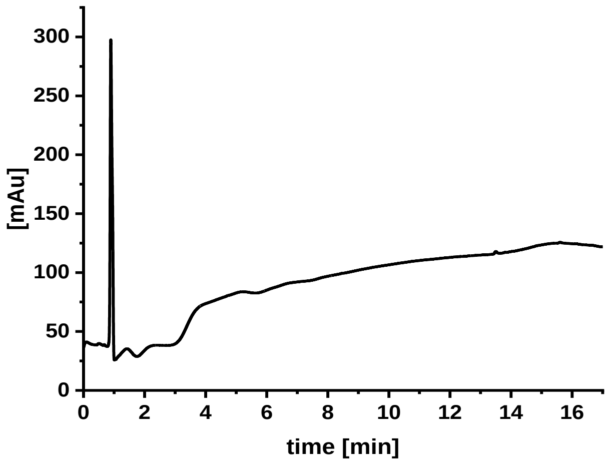

*Fig. S11: RP-HPCL chromatogram of α-D-ManEAA (**S3**) (gradient of 5 to 95 vol% acetonitrile/water with 0.1 vol% formic acid, run time: 17 min). The corresponding mass could be detected in the injection peak.*

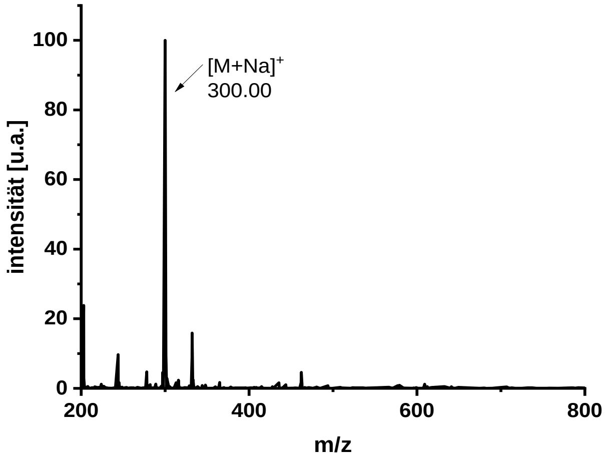

*Fig. S12: Mass spectrum of α-D-ManEAA (**S3**) at t = 1.00 min (gradient of 5 to 95 vol.% acetonitrile/water with 0.1 vol.% formic acid, run time: 17 min) by ESI-MS.*

S4 – β-D-Galactopyranosyl-ethylacrylamid (GalEAA)

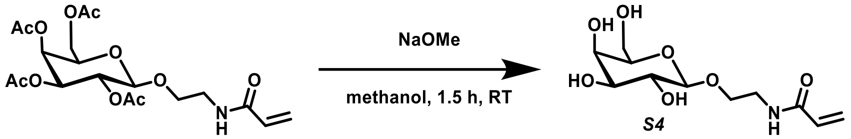

6.22 g (14.0 mmol) AcGalEAA ***S2*** was dissolved in 100 mL methanol, mixed with 190 mL 0.2 M sodium methanolate/methanol solution and stirred for 90 min. The organic solvent was removed under reduced pressure and the residue dissolved in water. Amberlite®IR-120 was subsequently added as ion exchange resin until pH 7 was reached. The exchange resin was filtered off and the product was lyophilized.

S4 was isolated as a whitish oil with a yield of 5.1 g (> 95%).

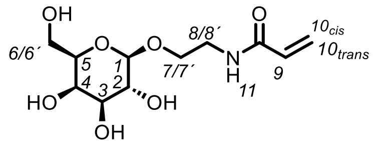

^{1}H-NMR (600 MHz, D_2O, 297.9 K) δ (ppm) 8.46 (s, 1H, *H11*), 6.35 – 6.26 (dd, ^3J = 17.2, 10.3 Hz, 1H, *H9*), 6.24 – 6.18 (dd, ^3J = 17.2, ^2J = 1.3 Hz, 1H, $H10_{cis}$), 5.80 – 5.75 (dd, ^3J = 10.3, ^2J = 1.3 Hz, 1H, $H10_{trans}$), 4.44 – 4.38 (d, ^3J = 7.9 Hz, 1H, *H1*), 4.05 – 3.46 (m, H11, *H2 – H5, H6a – H6b*, *H7a – H7b*, *H8a – H8b*).
LC-MS: m/z calculated for $C_{11}H_{19}NO_7$ $[M+Na]^+$ 300.12 found 300.00 in injection peak.

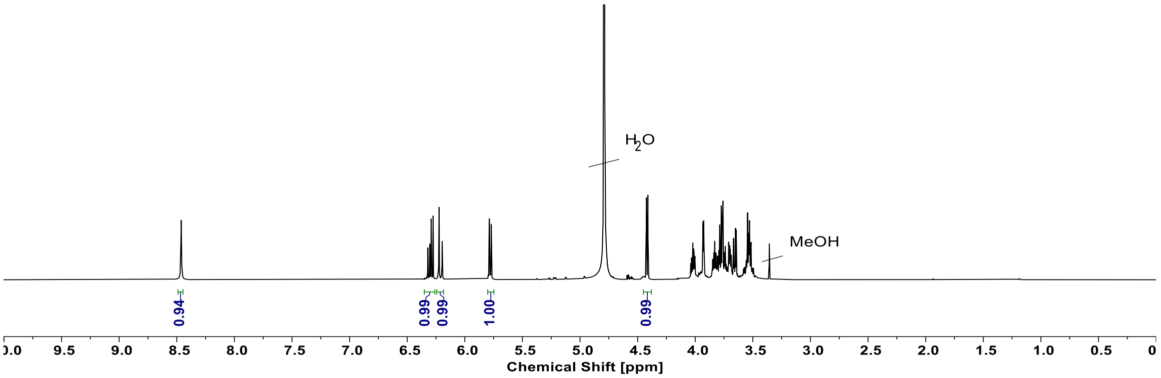

*Fig. S13: ^{1}H-NMR spectrum (600 MHz, D_2O, 297.9 K) of β-D-GalEAA (**S4**).*

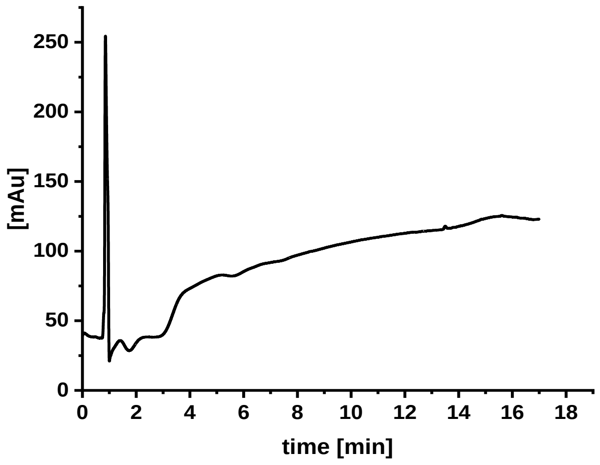

*Fig. S14: RP-HPCL chromatogram of β-D-GalEAA (**S4**) (gradient of 5 to 95 vol% acetonitrile/water with 0.1 vol% formic acid, run time: 17 min). The corresponding mass could be detected in the injection peak.*

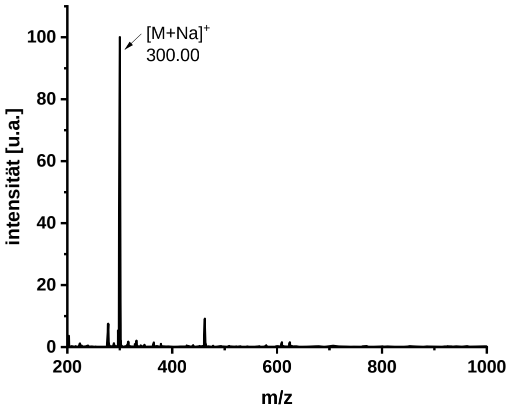

*Fig. S15: Mass spectrum of β-D-GalEAA (**S4**) at t = 1.00 min (gradient of 5 to 95 vol.% acetonitrile/water with 0.1 vol.% formic acid, run time: 17 min) by ESI-MS.*

2.2 Synthesis and measurements of BSA-polymer conjugates 1-4

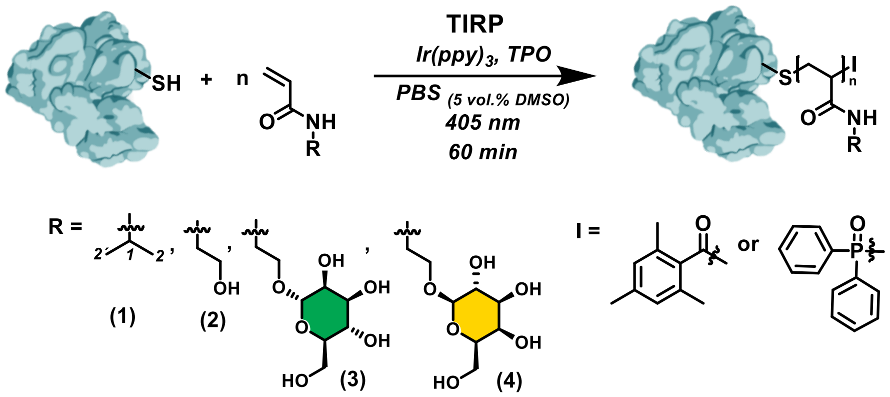

500 mg (7.5 µmol) of *bovine serum albumin* were dissolved in 5 mL of deoxygenated phosphate buffered saline (PBS buffer). Separate solutions *I-III* were prepared. *I*: **A** mg (375 µmol) of monomer in **B** µL of PBS buffer (50 mg/mL), *II*: 0.12 mg (0.18 µmol) of catalyst tris(2 phenylpyridine)iridium (Ir-Cat.) in 0.1 mL of dimethyl sulfoxide, *III*: 1.3 mg (3.75 µmol) of diphenyl(2,4,6-trimethylbenzoyl)phosphine oxide (TPO) in 0.1 mL of dimethyl sulfoxide. Ir-Cat. solution *II* was added to monomer solution *I* and deoxygenated with nitrogen for 10 min. In parallel, the TPO solution *III* was slowly drop-wise added to the protein solution and also deoxygenated with nitrogen for 10 min before it was irradiated in the focus of the UV lamp (λ = 405 nm) for 3 min at an intensity of 1.15 mW/cm^2. Subsequently, the mixture of monomer *I* and catalyst solution *II* was added to the protein and the entire mixture was irradiated for another 60 min at 5.22 mW/cm^2 intensity. The reaction mixture was purified by dialysis (exclusion volume 50 kDa) and then lyophilized. A white, fluffy powder was obtained.

Table 1: Formulation values of the monomers and buffer volumes of polymerizations.

**structure**	**monomer functionality**	**mass of monomer A [mg]**	**PBS buffer volume B [µL]**	**yields [mg]**
**1**	*N*-Hydroxyethyl acrylamide	43.2	864	518.9
**2**	*N*-Isopropyl acrylamide	42.4	849	523.6
**3**	ManEAA ***S3***	104.0	2080	539.2
**4**	GalEAA ***S4***	104.0	2080	547.8

S5 – BSA$_{native}$

^{1}H-NMR (600 MHz, D_2O, 297.9 K) δ (ppm) 9.17 – 6.29 (m, H_{BSA}), 4.56 – 0.00 (m, H_{BSA}).

AF4-LS (10 mM PBS, pH = 7.4, 298,15 K, whole fraction): Mn = 69.4 kDa, Mw = 74.2 kDa, Đ = 1.07, R_h = 3.5 nm.

IR (ATR) $\tilde{\nu}$ = 3281.68 cm^{-1} (broad O-H), 1644.11 cm^{-1} (stretch C=C), 1531.53 cm^{-1} (amide II), 1392.76 cm^{-1} (deformation C-H), 1242.77 cm^{-1} (deformation O-H).

CD: area 215 nm – 245 nm: 218.5 nm, -25.9 mdeg, area 198 nm – 215 nm: 209.0 nm, -27.9 mdeg

DSC: Enthalpy: 439 mJ, Enthalpy referenced to sample weight: 264 mJ/mg, Onset: 25 °C, Endset: 112 °C, Midpoint of thermal transition: 66 °C.

Ellman's Assay (amount of free thiols): 54%

SDS PAGE see Fig. S41.

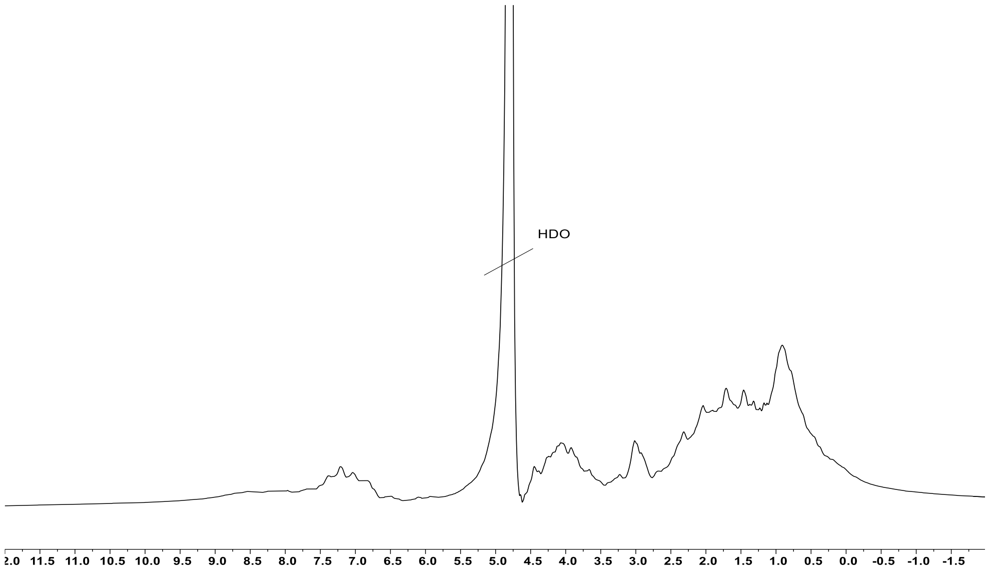

Fig. S16: ^{1}H-NMR spectrum (600 MHz, D_2O, 297.9 K) of BSA$_{native}$.

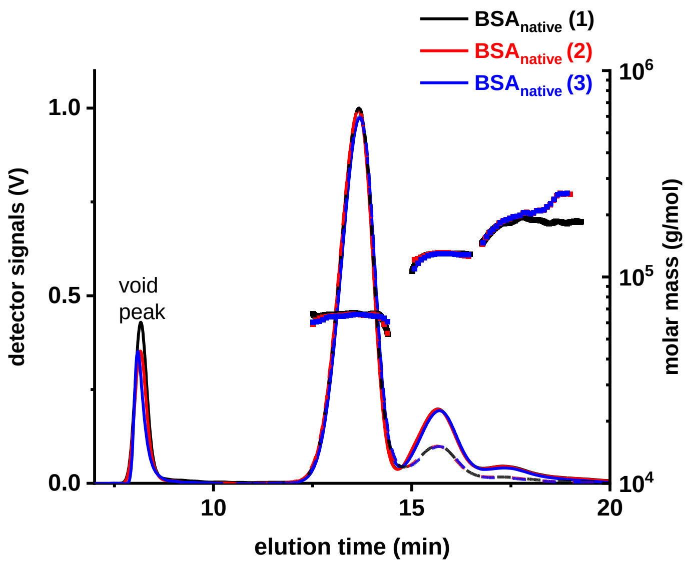

Fig. S17: Triplicate measurements and presentation of AF4-LS elugrams of BSA_{native} opt. separation, RI and LS signal, molar masses vs. elution time.

Table 2:Results of the triplicate AF4-LS measurements.

Measurement	M_n*1 (kg/mol)	M_w*1 (kg/mol)	Đ (M_w/M_n)	R_g (nm)	R_h (nm)
**1.** (50 µL)	69.4	74.5	1.07		3.5
**2.** (50 µL)	68.7	73.4	1.07		3.5
**3.** (50 µL)	70.1	74.6	1.06		3.5
**Average (whole Peak)**	**69.4**	**74.2**	**1.07**		**3.5**

*1 dn/dc = 0.185 mL/g

Table 3: Analysis of the individual fractions / populations of conjugates by AF4-LS. The values shown are average values from the triplicate measurements.

Fraction / population	M_n*1 (kg/mol)	M_w*1 (kg/mol)	Đ (M_w/M_n)	R_h (nm)	Mass fraction (%)	Mol fraction (%)
**1 - Monomer**	65.1	65.2	1.00	3.3	86.7	92.9
**2 – Dimer**	119	123	1.04	6.3	11.2	6.5
**3- Trimer / Multimer**	196	201	1.02	6.7	2.1	0.6

*1 dn/dc = 0.185 mL/g

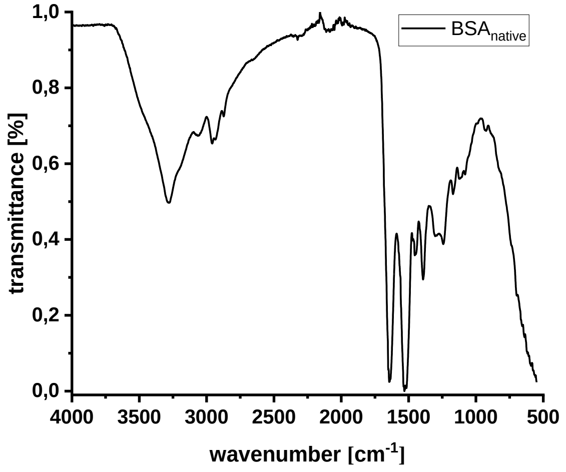

Fig. S18: IR spectrum of BSA$_{native}$.

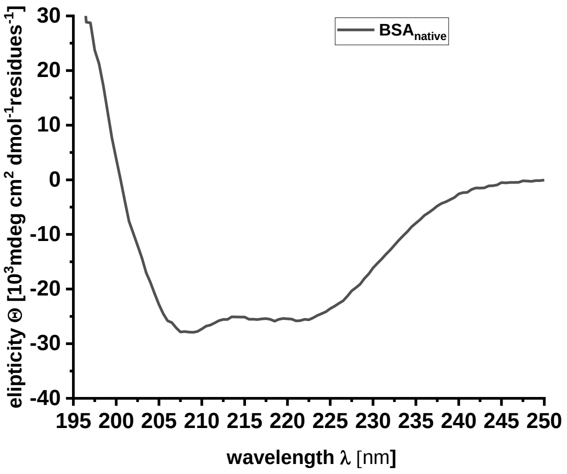

Fig. S19: CD spectrum of BSA$_{native}$.

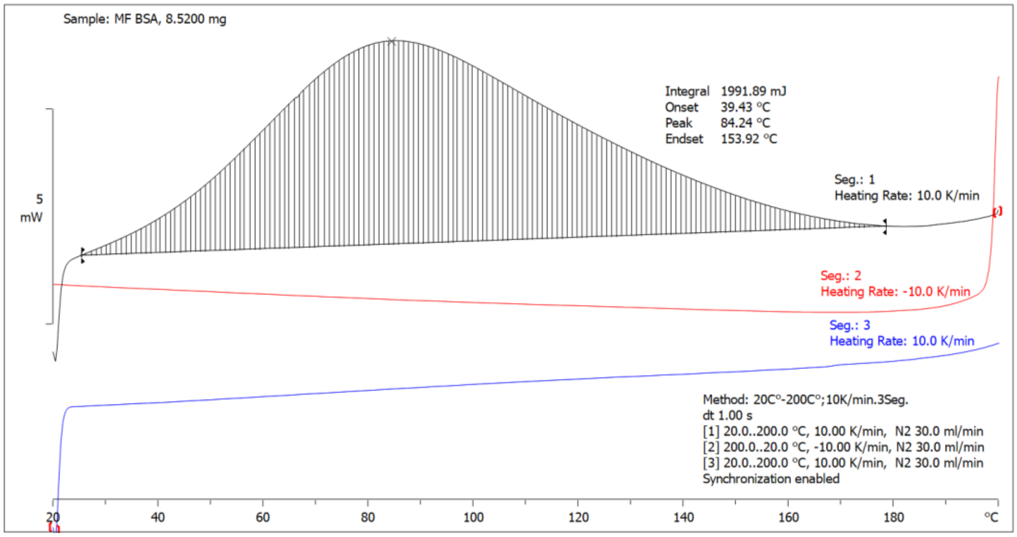

Fig. S20: DCS curves of BSA$_{native}$.

(1) – BSA-p(NIPAM) conjugate

^{1}H-NMR (600 MHz, D_2O, 297.9 K) δ (ppm) 9.17 – 6.40 (m, H_{BSA}), 4.57 – 0.00 (m, H_{BSA}, $H_{NIPAM\text{-}side\ chain}$, $H_{NIPAM\text{-}backbone}$ overlap with HDO peak), 3.91 (s_{br}, *H1*), 1.16 (s_{br}, *H2, H´2*).

AF4-LS (10 mM PBS, pH = 7.4, 298,15 K, whole fraction): Mn = 74.2 kDa, Mw = 80.2 kDa, Đ = 1.08, R_h = 3.2 nm.

IR (ATR) $\tilde{\nu}$ = 1077.24 cm^{-1} (deformation C-OH). All other wavelengths identical to BSA.

CD: area 215 nm – 245 nm: 220.0 nm, -28.6 mdeg, area 198 nm – 215 nm: 208.0 nm, -30.7 mdeg.

DSC: Enthalpy: 362 mJ, Enthalpy referenced to sample weight: 212 mJ/mg, Onset: 27 °C, Endset: 106 °C, Midpoint of thermal transition: 64 °C.

SDS PAGE see Fig. S41.

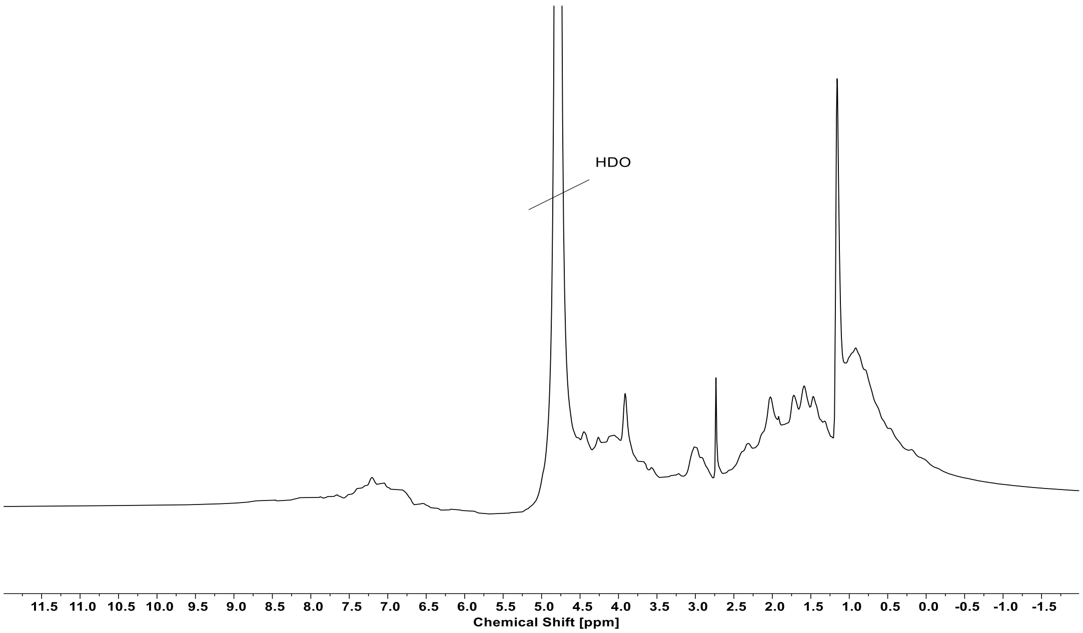

*Fig. S21: ^{1}H-NMR spectrum (600 MHz, D_2O, 297.9 K) of BSA-p(NIPAM) conjugate (**1**).*

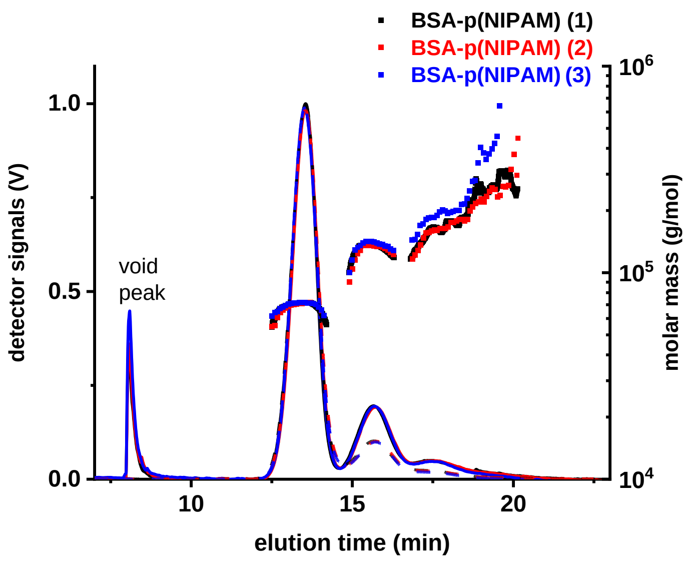

*Fig. S22: Triplicate measurements and presentation of AF4-LS elugrams of BSA-p(NIPAM) conjugate (**1**) opt. separation, RI and LS signal, molar masses vs. elution time.*

Table 4: Results of the triplicate AF4-LS measurements.

**Measurement**	M_n *1 (kg/mol)	M_w *1 (kg/mol)	**Đ** (M_w/M_n)	R_g (nm)	R_h (nm)
**1.** (50 µL)	74.2	79.9	1.08	*6.4*	3.2
**2.** (50 µL)	73.7	79.3	1.08		3.3
**3.** (50 µL)	74.9	81.3	1.09	*6.3*	3.1
**Average (whole Peak)**	**74.2**	**80.2**	**1.08**		**3.2**

*1 dn/dc = 0.172 mL/g

Table 5: Analysis of the individual fractions / populations of conjugates by AF4-LS. The values shown are average values from the triplicate measurements.

**Fraction / population**	M_n *1 (kg/mol)	M_w *1 (kg/mol)	**Đ** (M_w/M_n)	R_h (nm)	**Mass fraction** (%)	**Mol fraction** (%)
1 - Monomer	70.5	70.6	1.00	3.1	93.1	91.5
2 – Dimer	134	134	1.00		5.7	7.0
3- Trimer / Multimer	167	167	1.00	7.4	1.2	1.5

*1 dn/dc = 0.172 mL/g

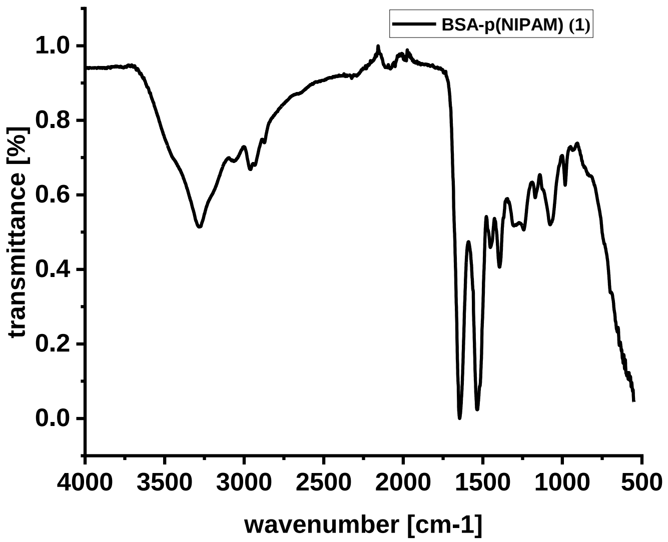

*Fig. S23: IR spectrum BSA-p(NIPAM) conjugate (**1**).*

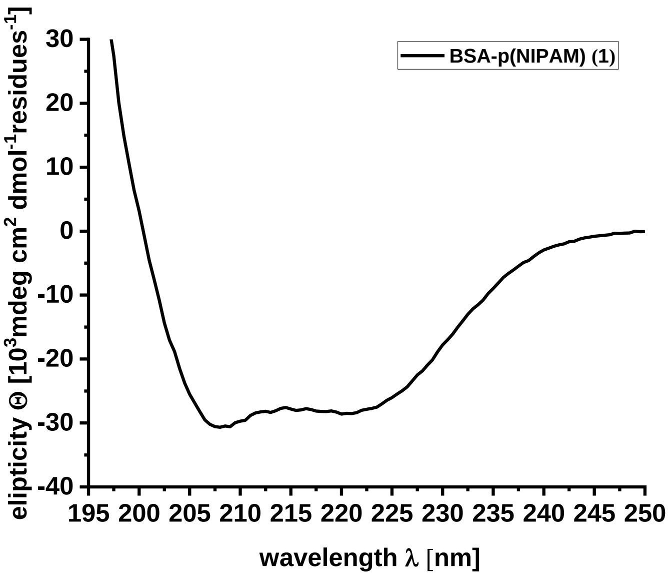

*Fig. S24: CD spectrum of BSA-p(NIPAM) conjugate (**1**).*

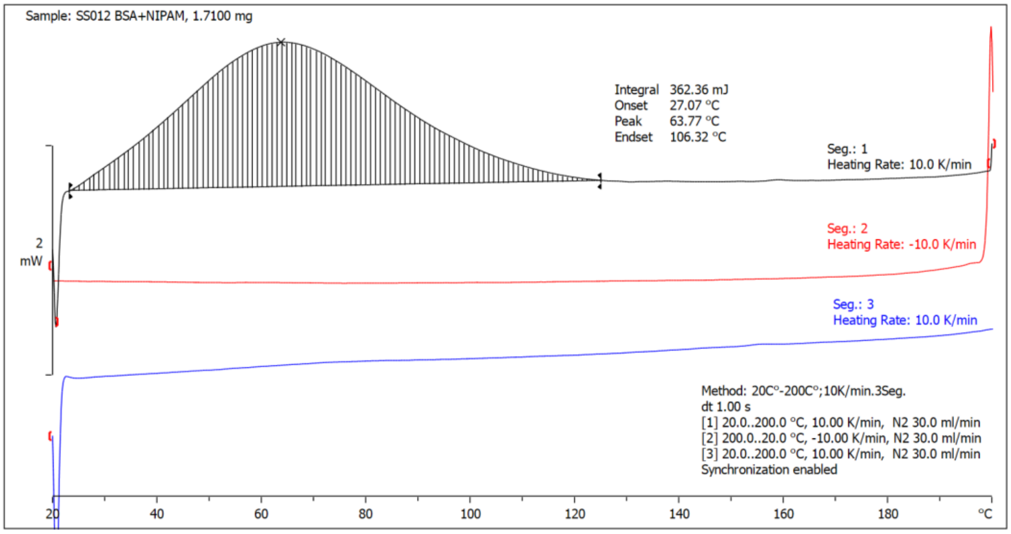

*Fig. S25: DSC curve of BSA-p(NIPAM) conjugate (**1**).*

(2) – BSA-p(HEAA) conjugate

^{1}H-NMR (600 MHz, D_2O, 297.9 K) δ (ppm) 9.17 – 6.46 (m,H_{BSA}), 4.57 – 0.00 (m, H_{BSA}, $H_{HEAA\text{-}side\ chain}$, $H_{HEAA\text{-}backbone}$ overlap with HDO peak), 4.35 – 3.13 (m, $H_{HEAA\text{-}side\ chain}$).

AF4-LS (10 mM PBS, pH = 7.4, 298,15 K, whole fraction): Mn = 71.5 kDa, Mw = 77.0 kDa, Đ = 1.08, R_h = 4.1 nm.

IR (ATR) $\tilde{\nu}$ = 1066.41 cm^{-1} (deformation C-OH). All other wavelengths identical to BSA.

CD: area 215 nm – 245 nm: 219.5 nm, -30.7 mdeg, area 198 nm – 215 nm: 209.0 nm, -32.7 mdeg.

DSC: Enthalpy: 522 mJ, Enthalpy referenced to sample weight: 211 mJ/mg, Onset: 30 °C, Endset: 116 °C, Midpoint of thermal transition: 70 °C.

SDS PAGE see Fig. S41.

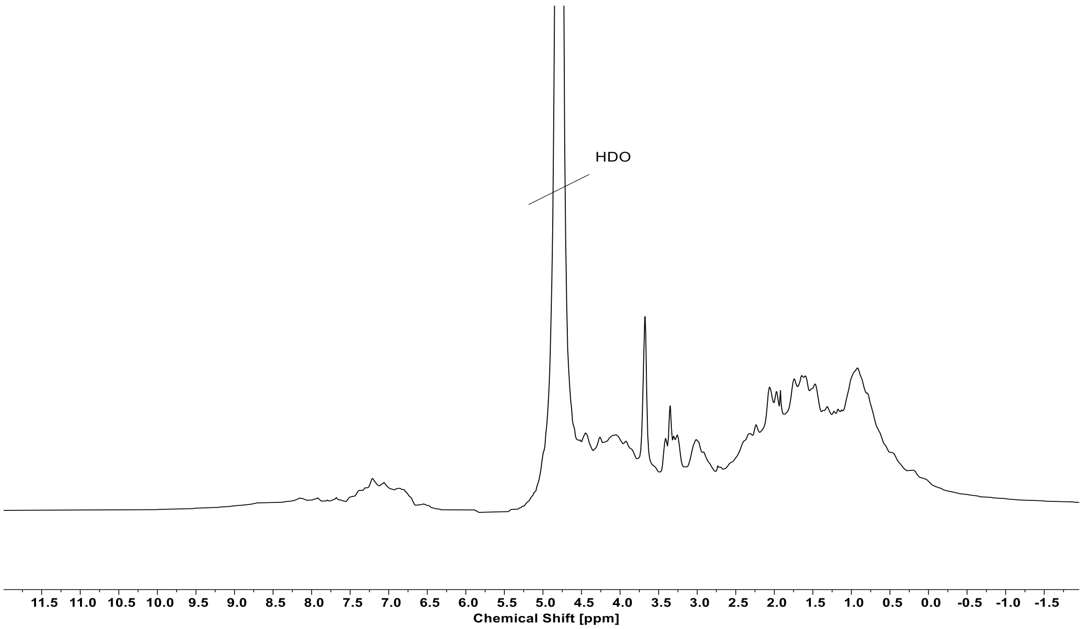

*Fig. S26: ^{1}H-NMR spectrum (600 MHz, D_2O, 297.9 K) of BSA-p(HEAA) conjugate (**2**).*

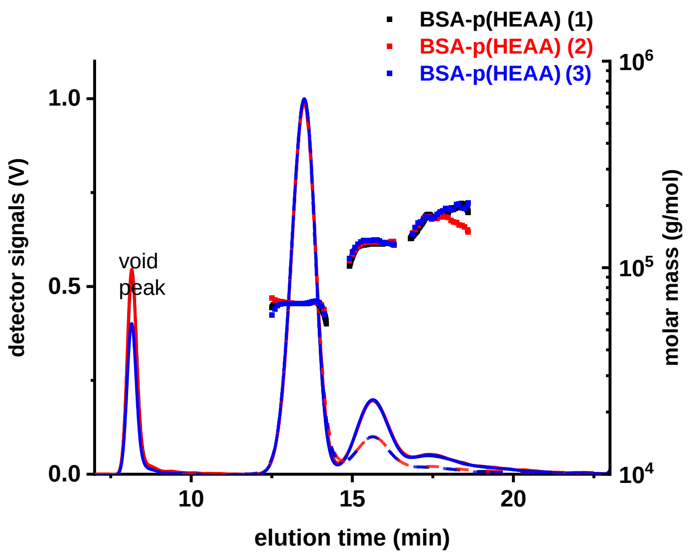

*Fig. S27: Triplicate measurements and presentation of AF4-LS elugrams of BSA-p(HEAA) conjugate (**2**) opt. separation, RI and LS signal, molar masses vs. elution time.*

Table 6: Results of the triplicate AF4-LS measurements.

**Measurement**	**M_n*1** (kg/mol)	**M_w*1** (kg/mol)	**Đ** (M_w/M_n)	**R_g** (nm)	**R_h** (nm)
**1.** (50 µL)	71.5	77.0	1.08	*6.8*	4.0
**2.** (50 µL)	71.4	76.7	1.07	*9.2*	5.0
**3.** (50 µL)	71.5	77.4	1.08	*5.8*	3.4
**Average (whole Peak)**	**71.5**	**77.0**	**1.08**	***7.3***	**4.1**

*1 dn/dc = 0.181 mL/g

Table 7: Analysis of the individual fractions / populations of conjugates by AF4-LS. The values shown are average values from the triplicate measurements.

**Fraction / population**	**M_n*1** (kg/mol)	**M_w*1** (kg/mol)	**Đ** (M_w/M_n)	**R_h** (nm)	**Mass fraction** (%)	**Mol fraction** (%)
1 - Monomer	66.1	66.3	1.00	3.0	84.9	92.2
2 – Dimer	126	128	1.01	6.1	11.8	6.6
3- Trimer / Multimer	171	174	1.01	8.8	3.3	1.2

*1 dn/dc = 0.181 mL/g

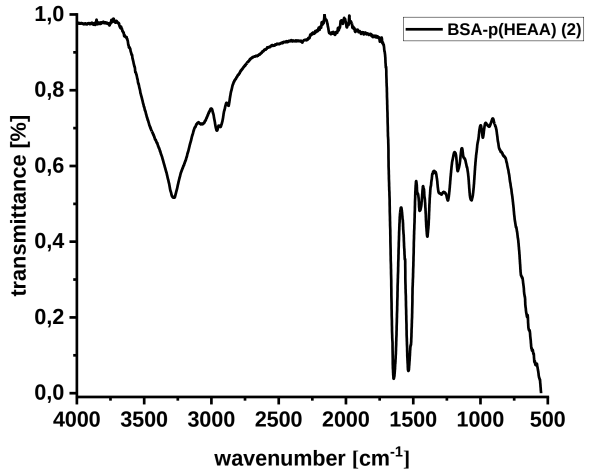

*Fig. S28: IR spectrum of BSA-p(HEAA) conjugate (**2**).*

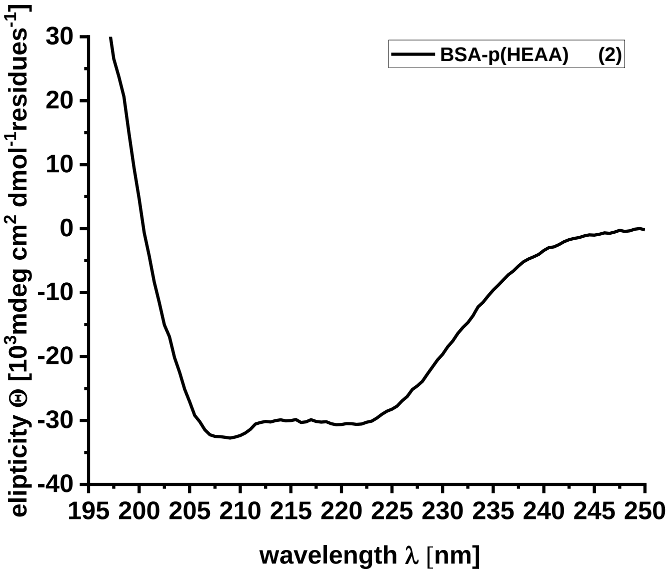

*Fig. S29: CD spectrum of BSA-p(HEAA) conjugate (**2**).*

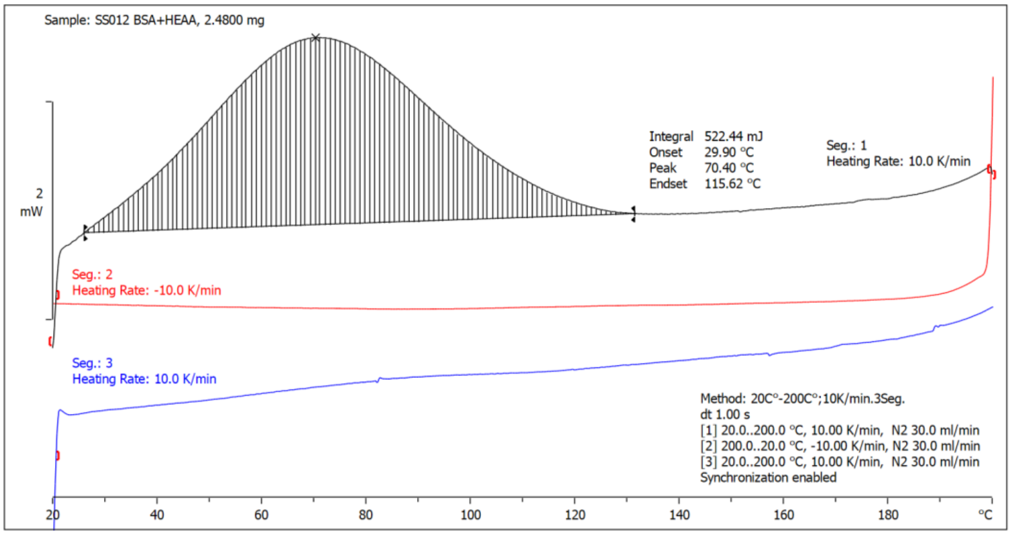

*Fig. S30: DSC curve of BSA-p(HEAA) conjugate (**2**).*

(3) – BSA-p(ManEAA) conjugate

^{1}H-NMR (600 MHz, D_2O, 297.9 K) δ (ppm) 9.17 – 6.46 (m, H_{BSA}), 4.57 – 0.00 (m, H_{BSA}, $H_{ManEAA\text{-}side\ chain}$, $H_{ManEAA\text{-}backbone}$ overlap with HDO peak), 4.50 – 3.40 (m, $H_{ManEAA\text{-}side\ chaine}$).

AF4-LS (10 mM PBS, pH = 7.4, 298,15 K, whole fraction): Mn = 81.9 kDa, Mw = 88.2 kDa, Đ = 1.08, R_h = 3.1 nm.

IR (ATR) $\tilde{\nu}$ = 1065.87 cm^{-1} (deformation C-OH). All other wavelengths identical to BSA.

CD: area 215 nm – 245 nm: 221.5 nm, -26.6 mdeg, area 198 nm – 215 nm: 208.5 nm, -28.9 mdeg.

DSC: Enthalpy: 534 mJ, Enthalpy referenced to sample weight: 243 mJ/mg, Onset: 28 °C, Endset: 117 °C, Midpoint of thermal transition: 67 °C.

SDS PAGE see Fig. S41.

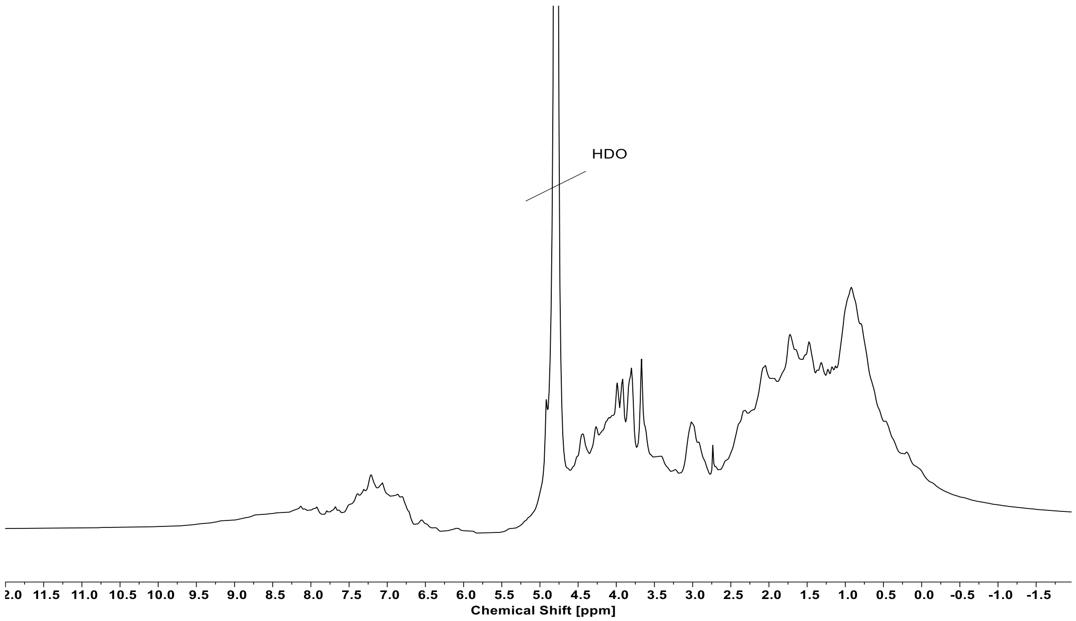

*Fig. S31: ^{1}H-NMR spectrum (600 MHz, D_2O, 297.9 K) of BSA-p(ManEAA) conjugate (**3**).*

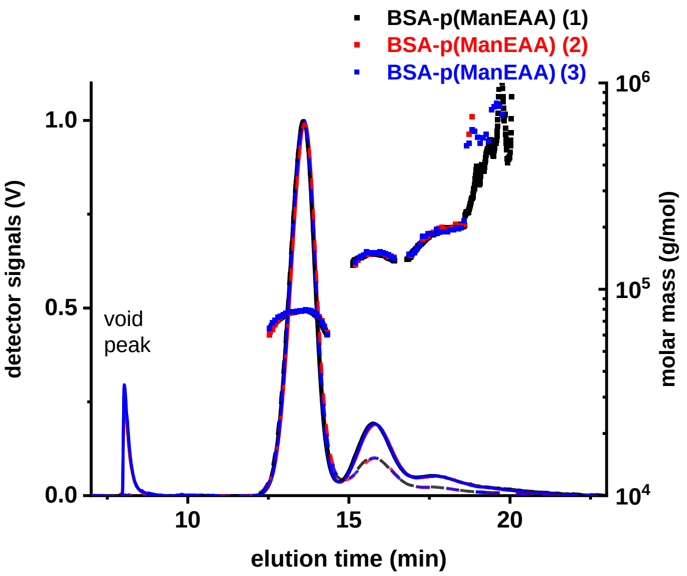

*Fig. S32: Triplicate measurements and presentation of AF4-LS elugrams of BSA-p(ManEAA) conjugate (**3**) opt. separation, RI and LS signal, molar masses vs. elution time.*

Table 8: Results of the triplicate AF4-LS measurements.

**Measurement**	**M_n**[*1] (kg/mol)	**M_w**[*1] (kg/mol)	**Đ** (M_w/M_n)	**R_g** (nm)	**R_h** (nm)
**1.** (50 µL)	81.7	88.2	1.08	*6.4*	3.1
**2.** (50 µL)	81.6	87.8	1.08	*3.3*	3.1
**3.** (50 µL)	82.4	88.8	1.08	*9.3*	3.0
**Average (whole Peak)**	**81.9**	**88.2**	**1.08**	***6.3***	**3.1**

*1 dn/dc = 0.158 mL/g

Table 9: Analysis of the individual fractions / populations of conjugates by AF4-LS. The values shown are average values from the triplicate measurements.

**Fraction / population**	**M_n**[*1] (kg/mol)	**M_w**[*1] (kg/mol)	**Đ** (M_w/M_n)	**R_h** (nm)	**Mass fraction** (%)	**Mol fraction** (%)
1 - Monomer	78.1	78.2	1.00	3.2	83.7	91.2
2 – Dimer	148	148	1.00	4.5	12.7	7.3
3- Trimer / Multimer	198	198	1.00		3.6	1.5

*1 dn/dc = 0.158 mL/g

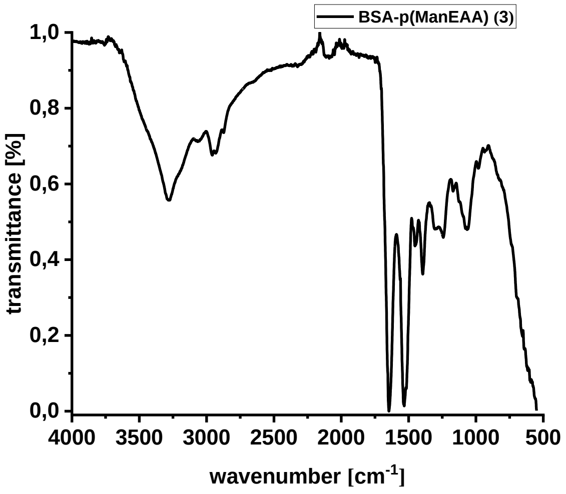

*Fig. S33: IR spectrum of BSA-p(ManEAA) conjugate (**3**).*

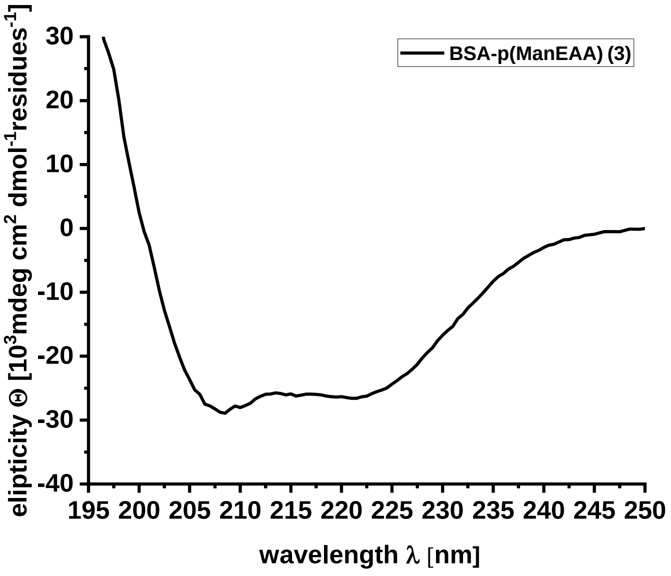

*Fig. S34: CD spectrum of BSA-p(ManEAA) conjugate (**3**).*

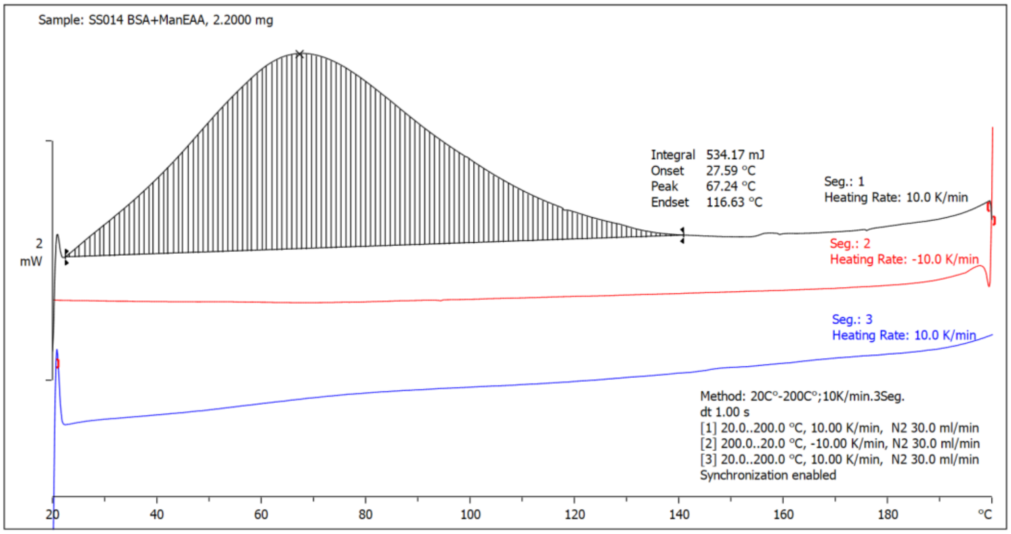

*Fig. S35: DSC curve of BSA-p(ManEAA) conjugate (**3**).*

(4) – BSA-p(GalEAA) conjugate

^{1}H-NMR (600 MHz, D_2O, 297.9 K) δ (ppm) 9.17 – 6.46 (m, H_{BSA}), 4.57 – 0.00 (m, H_{BSA}, $H_{GalEAA\text{-side chain}}$, $H_{GalEAA\text{-backbone}}$ overlap with HDO peak), 4.53 – 3.37 (m, $H_{GalEAA\text{-side chain}}$).

AF4-LS (10 mM PBS, pH = 7.4, 298,15 K, whole fraction): Mn = 81.9 kDa, Mw = 88.3 kDa, Đ = 1.08, R_h = 3.2 nm.

IR (ATR) $\tilde{\nu}$ = 1017.00 cm^{-1} (deformation C-OH). All other wavelengths identical to BSA.

CD: area 215 nm – 245 nm: 220.5 nm, -26.3 mdeg, area 198 nm – 215 nm: 208.0 nm, -28.5 mdeg.

DSC: Enthalpy: 460 mJ, Enthalpy referenced to sample weight: 171 mJ/mg, Onset: 30 °C, Endset: 104 °C, Midpoint of thermal transition: 69 °C.

SDS PAGE see Fig. S41.

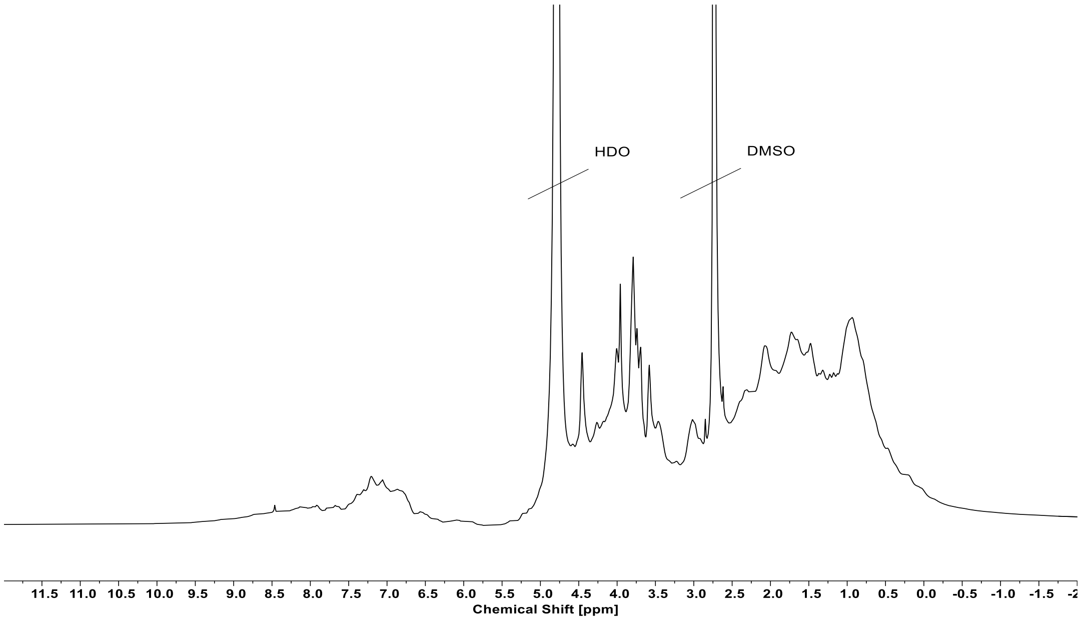

*Fig. S36: ^{1}H-NMR spectrum (600 MHz, D_2O, 297.9 K) of BSA-p(GalEAA) conjugate (**4**).*

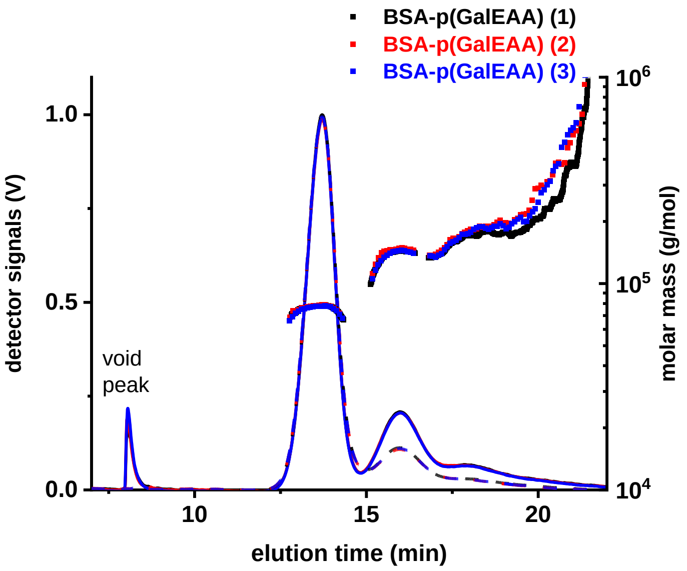

*Fig. S37: Triplicate measurements and presentation of AF4-LS elugrams of BSA-p(GalEAA) conjugate (**4**) opt. separation, RI and LS signal, molar masses vs. elution time.*

Table 10: Results of the triplicate AF4-LS measurements.

**Measurement**	**M_n*1** (kg/mol)	**M_w*1** (kg/mol)	**Đ** (M_w/M_n)	**R_g** (nm)	**R_h** (nm)
**1.** (50 µL)	82.6	88.1	1.07		3.2
**2.** (50 µL)	82.4	89.1	1.08		3.3
**3.** (50 µL)	81.1	87.6	1.08		3.2
**Average (whole Peak)**	**81.9**	**88.3**	**1.08**		**3.2**

*1 dn/dc = 0.158 mL/g

Table 11: Analysis of the individual fractions / populations of conjugates by AF4-LS. The values shown are average values from the triplicate measurements.

**Fraction / population**	**M_n*1** (kg/mol)	**M_w*1** (kg/mol)	**Đ** (M_w/M_n)	**R_h** (nm)	**Mass fraction** (%)	**Mol fraction** (%)
1 - Monomer	77.1	77.1	1.00	3.2	81.1	89.6
2 – Dimer	144	144	1.00	3.9	14.3	8.4
3- Trimer / Multimer	186	186	1.00	5.6	4.6	2.0

*1 dn/dc = 0.158 mL/g

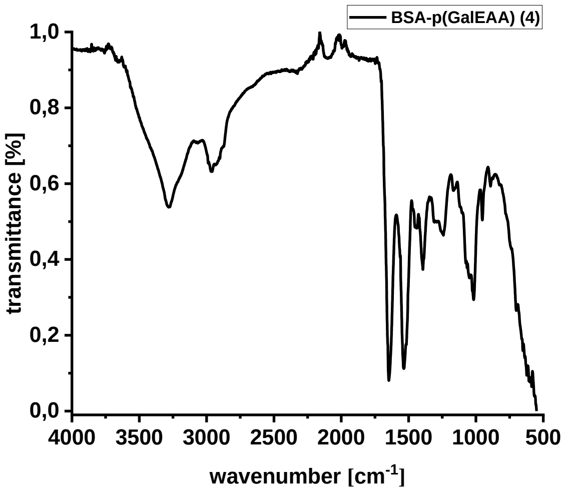

*Fig. S38: IR spectrum of BSA-p(GalEAA) conjugate (**4**).*

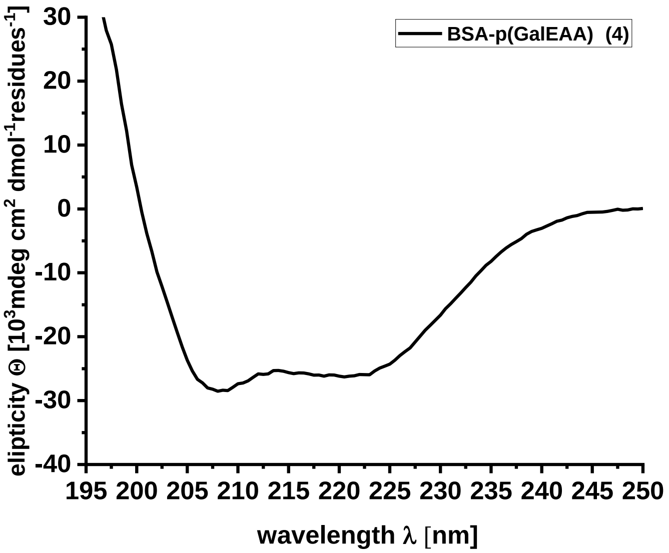

*Fig. S39: CD spectrum of BSA-p(GalEAA) conjugate (**4**).*

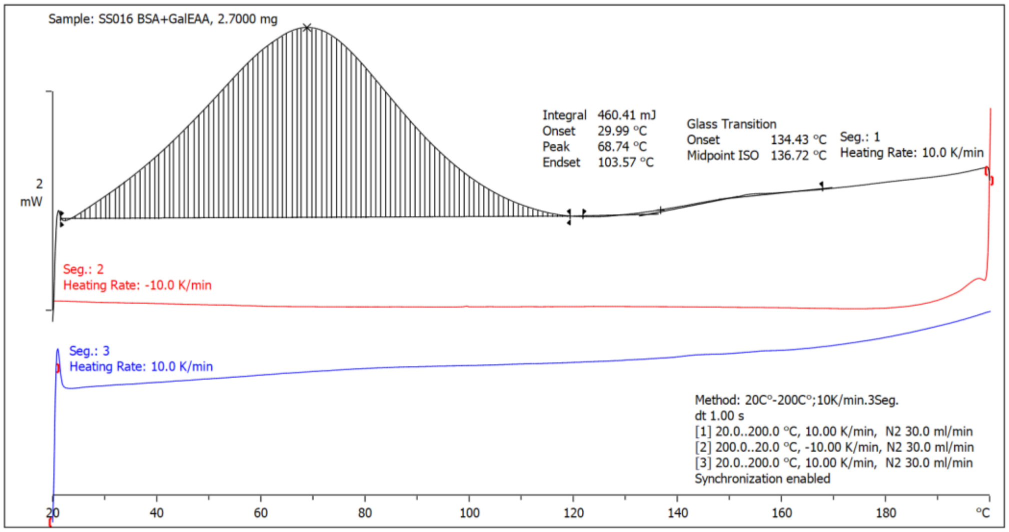

*Fig. S40: DSC curve of BSA-p(GalEAA) conjugate (**4**).*

SDS-PAGE

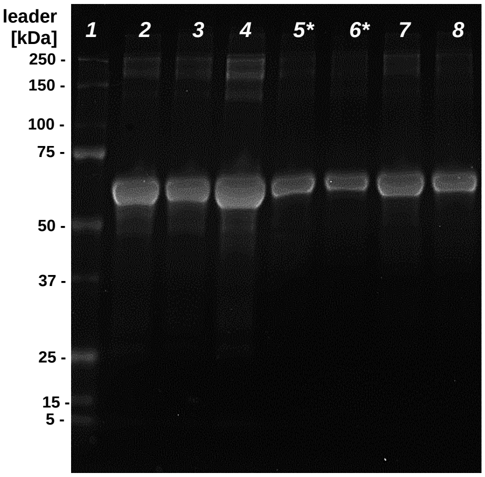

lane	*loading*
1	protein leader
2	BSA_{native}
3	BSA-p(HEAA)
4	BSA-p(NIPAM)
5	$BSA\text{-}p(NIPAM)_{100}$*
6	$BSA\text{-}p(NIPAM)_{200}$*
7	BSA-p(ManEAA)
8	BSA-p(GalEAA)

*Fig. S41: SDS PAGE of BSA_{native} (**S5**), BSA-p(NIPAM) (**1**), BSA-p(HEAA) (**2**), BSA-p(ManEAA) (**3**), BSA-p(GalEAA) (**4**). *These structures were loaded as references of higher MW conjugate samples but are not discussed in this paper.*

Summary of CD measurements

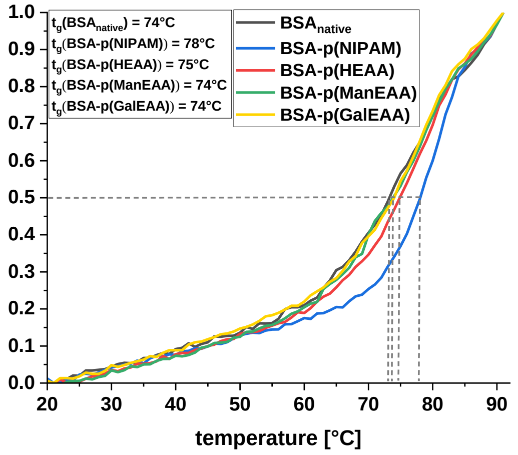

*Fig. S42: Denaturation studies measuring the temperature-dependent ellipticity at 222 nm of BSA_{native} (**S6**), BSA-p(NIPAM) (**1**), BSA-p(HEAA) (**2**), BSA-p(ManEAA) (**3**), BSA-p(GalEAA) (**4**). Temperature steps from 20 – 90 °C in PBS buffer at pH 7.4.*

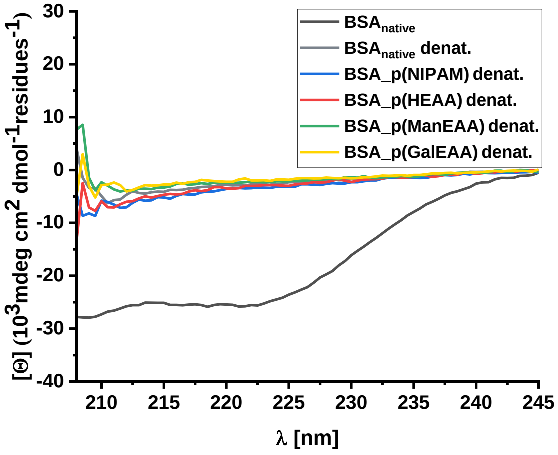

*Fig. S43: Denaturation studies in 6 M Guanidinium chloride and under influence of temperature (30 °C for 120 min) of BSA_{native} (**S6**), BSA-p(NIPAM) (**1**), BSA-p(HEAA) (**2**), BSA-p(ManEAA) (**3**), BSA-p(GalEAA) (**4**). CD spectra were recorded between 208 nm and 245 nm in PBS buffer at pH 7.4.*

Determination of the half-maximum turbidity

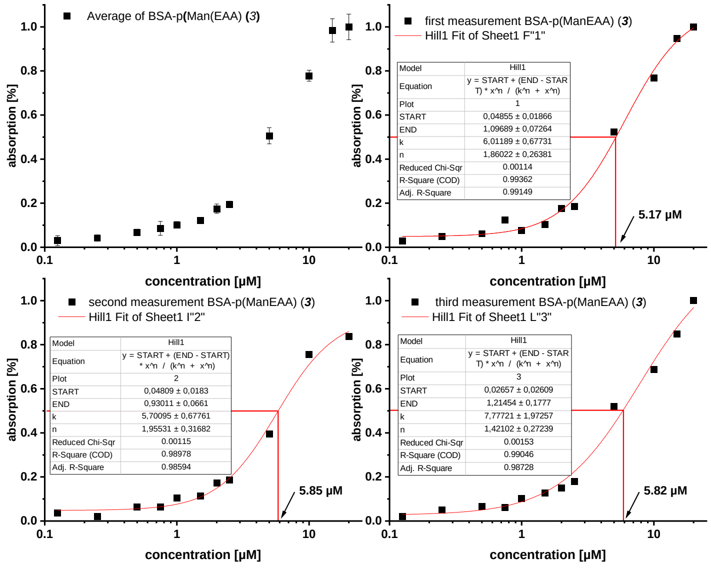

*Fig. S44: Compilation of the individual measurements of BSA-p(ManEAA) (**3**) with corresponding values of the half-maximum turbidity. The mean value of the individual measurements is 5.61 ± 0.38 µM.*

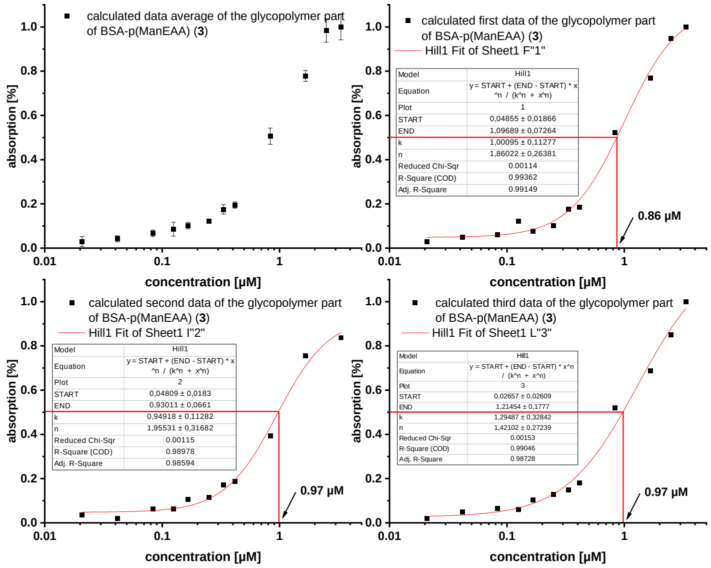

*Fig. S45: Calculated data of the glycopolymer part of BSA-p(ManEAA) (**3**) conjugate with corresponding values of the half-maximum turbidity. The purpose of the calculations was to determine the value of the half-maximum turbidity independent of the molecular weight of the protein, by solely considering the quantity of sugar present. The mean value of the individual measurements is 0.94 ± 0.06 µM.*

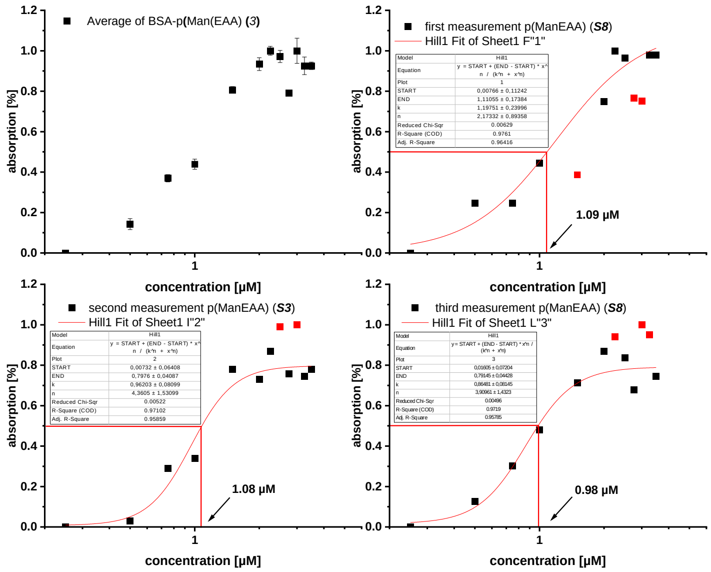

*Fig. S46: Compilation of the individual measurements of p(ManEAA) (**S7**) with corresponding values of the half-maximum turbidity. The mean value of the individual measurements is 1.05 ± 0.06 µM.*

Ellman's Assay

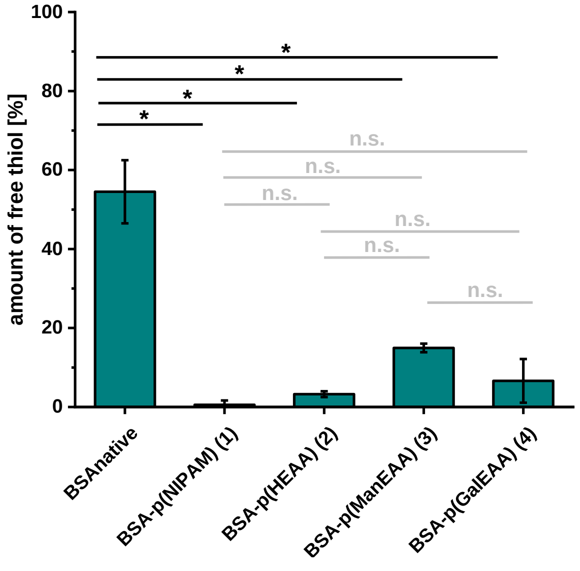

*Fig. S47: Results of the Ellman's assay of native BSA in comparison to conjugates **1**-**4**. 196 µL of Ellman's buffer was mixed with 20 µL of the protein stock solution (0.75 mM in Ellman's buffer) followed by 4 µL of the Ellman's stock solution (1 mg/mL in Ellman's buffer) and incubated for 15 min. The absorbance was measured at 412 nm and the amount of accessible thiols was determined based on a previously established calibration curve. Data were evaluated using one-way ANOVA analysis followed by Bonferroni correction (*<0.05, not significant (n.s.)). The reduced amount of free thiol is indicative of a polymer conjugation via the free thiol of the BSA.*

2.3 Synthesis and measurements of CRL-polymer conjugates 5-7

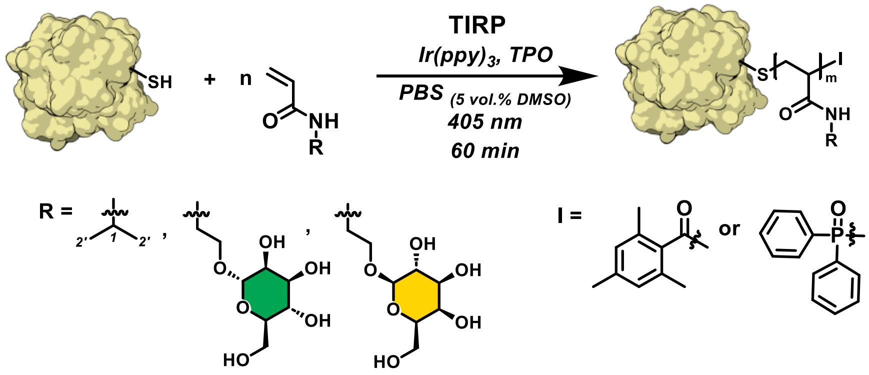

200 mg (3.47 µmol) of *Candida Rugose Lipase* was dissolved in 4 mL of deoxygenated phosphate buffered saline (PBS buffer). Separate solutions *I-III* were prepared. *I*: **A** mg (**B** µmol) of monomer in **C** µL of PBS buffer (50 mg/mL), *II*: **D** mg (**E** µmol) of catalyst tris(2 phenylpyridine)iridium (Ir-Cat.) in 0.1 mL of dimethyl sulfoxide, *III*: 0.6 mg (1.73 µmol) of diphenyl(2,4,6-trimethylbenzoyl)phosphine oxide (TPO) in 0.1 mL of dimethyl sulfoxide. Ir-Cat. solution *II* was added to monomer solution *I* and deoxygenated with nitrogen for 10 min. In parallel, the TPO solution *III* was slowly dropped to the protein solution and also deoxygenated with nitrogen for 10 min before it was irradiated in the focus of the UV lamp (λ = 405 nm) for 10 min at an intensity of 1.15 mW/cm^2. Subsequently, the mixture of monomer *I* and catalyst solution *II* was added to the protein and the entire mixture was irradiated for another 60 min at 5.22 mW/cm^2 intensity. The reaction mixture was purified by dialysis (exclusion volume 50 kDa) and then lyophilized. A white, fluffy powder was obtained.

Table 12: Formulation values of the monomers and buffer volumes of polymerizations.

structure	monomer functionality	mass of monomer A [mg]	amount of monomer A [µmol]	PBS buffer volume B [µL]	mass of Ir-Cat. D [mg]	amount of Ir-Cat. E [µmol]	yields [mg]
**5**	*N*-Isopropyl acrylamide	78.6	694.6	1572	0.22	0.35	254
**6**	ManEAA ***S4***	48.7	173.7	974	0.06	0.09	197
**7**	GalEAA ***S5***	48.7	173.7	974	0.06	0.09	216

S6 – CRL$_{native}$

^{1}H-NMR (600 MHz, D_2O, 297.9 K) δ (ppm) 8.56 – 6.62 (m, H_{CRL}), 4.43 – 0.43 (m, H_{BSA} overlaps with HDO peak).

DLS: 345.1 ± 80.4 nm

Ellman's Assay (amount of free thiols): 95%

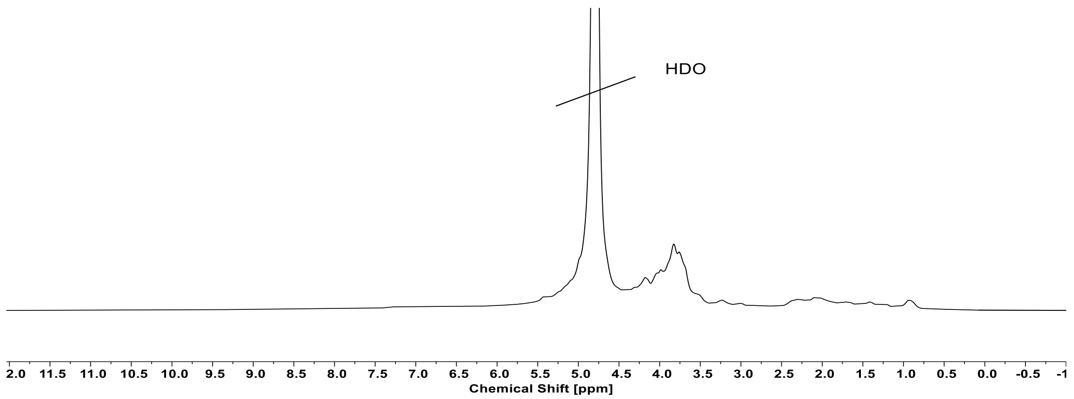

Fig. S48: ^{1}H-NMR spectrum (600 MHz, D2O, 297.9 K) of CRL$_{native}$.

(5) – CRL-p(NIPAM)

^{1}H-NMR (600 MHz, D_2O, 297.9 K) δ (ppm) 8.16 – 6.06 (m, H_{CRL}), 4.42 – 0.49 (m, H_{CRL}, $H_{NIPAM\text{-}side\ chain}$, $H_{NIPAM\text{-}backbone}$ overlap with HDO peak), 2.22 – 1.36 (m, $H_{NIPAM\text{-}backbone}$ overlaps with H_{CRL} peak), 3.92 (s_{br}, *H1* overlap with H_{CRL} peak), 1.18 (s_{br}, *H2, H´2*).

DLS: 121.2 ± 17.3 nm

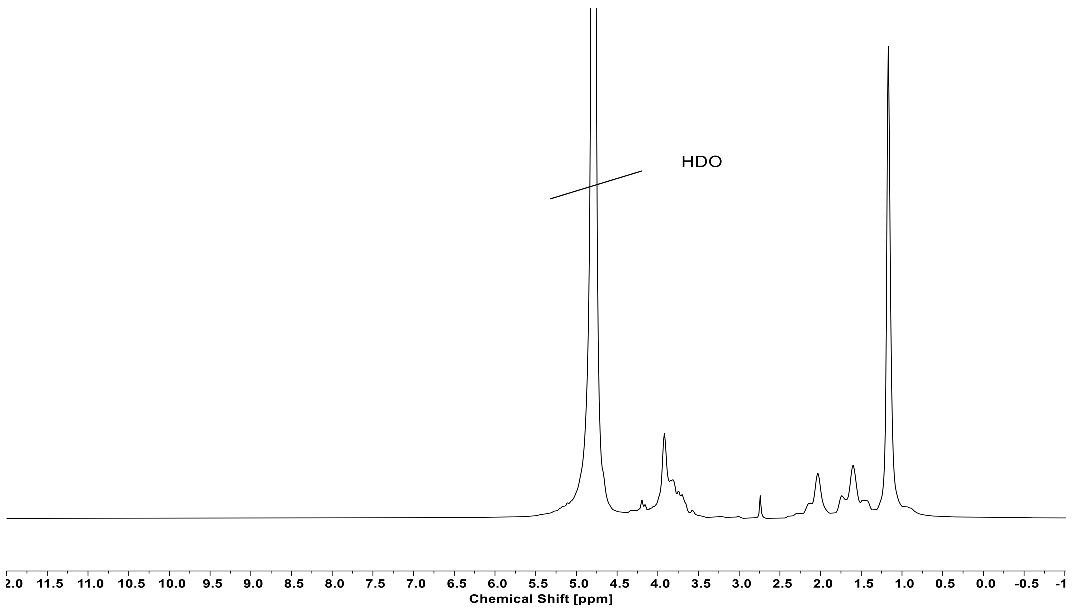

*Fig. S49: ^{1}H-NMR spectrum (600 MHz, D_2O, 297.9 K) of CRL-p(NIPAM) (**5**).*

(6) – CRL-p(ManEAA)

^{1}H-NMR (600 MHz, D_2O, 297.9 K) δ (ppm) 8.24 – 6.16 (m, H_{CRL}), 4.39 – 0.63 (m, H_{CRL}, $H_{ManEAA\text{-}side\ chain}$, $H_{ManEAA\text{-}backbone}$ overlap with HDO peak).

DLS: 129.7 ± 8.6 nm

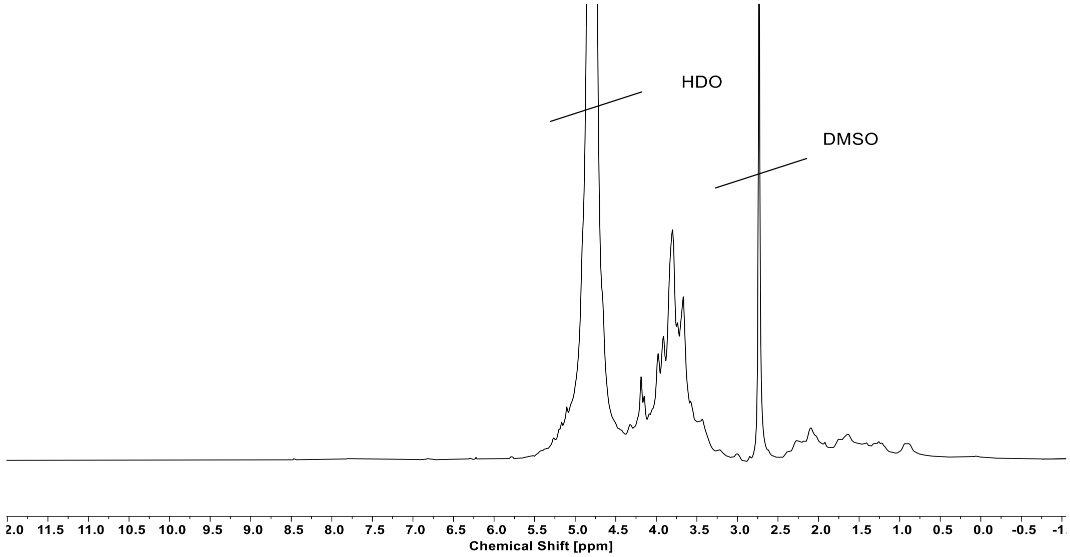

*Fig. S50: ^{1}H-NMR spectrum (600 MHz, D2O, 297.9 K) of CRL-p(ManEAA) (**6**).*

(7) – CRL-p(GalEAA)

^{1}H-NMR (600 MHz, D_2O, 297.9 K) δ (ppm) 8.47 – 6.17 (m, H_{CRL}), 4.46 (s, $H_{GalEAA\text{-}anomeric\ center}$) 4.36 – 0.47 (m, H_{CRL}, $H_{ManEAA\text{-}side\ chain}$, $H_{ManEAA\text{-}backbone}$).

DLS: 125.6 ± 13.2 nm

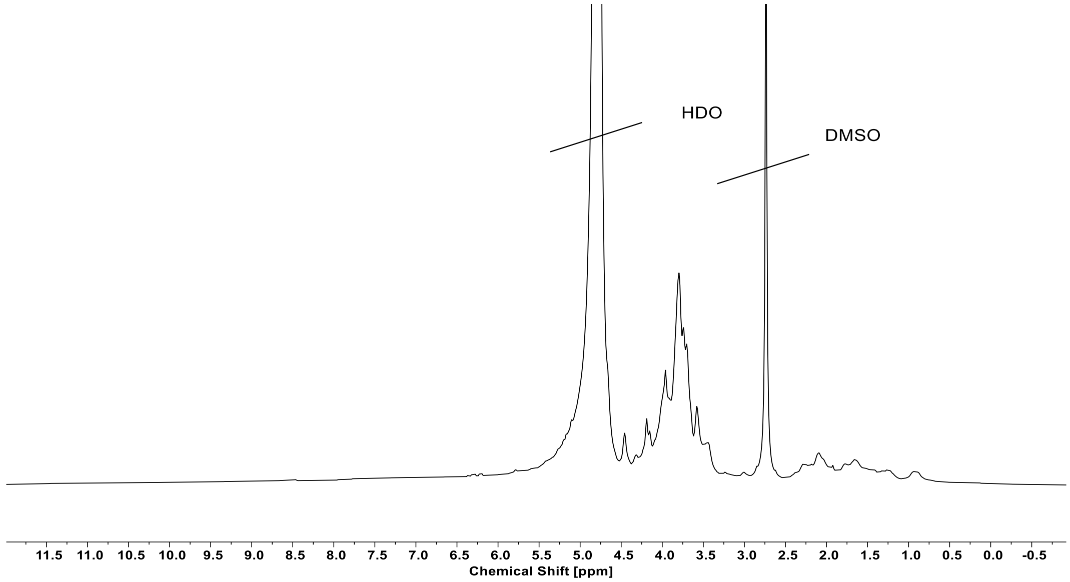

*Fig. S51: ^{1}H-NMR spectrum (600 MHz, D_2O, 297.9 K) of CRL-p(GalEAA) (**7**).*

SDS-PAGE

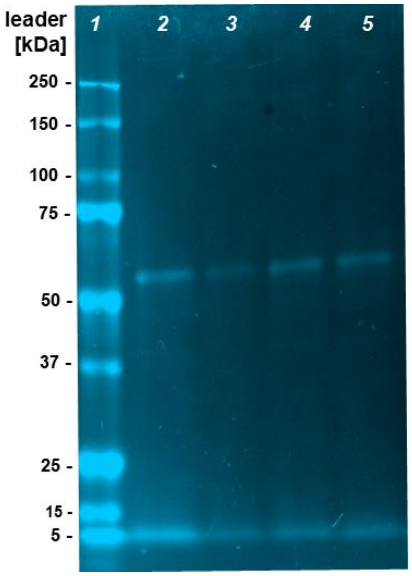

lane	*loading*
1	protein leader
2	CRL_{native}
3	CRL_p(NIPAM)
4	CRL_p(ManEAA)
5	CRL_p(GalEAA)

*Fig. S52: SDS PAGE with 2 mg/mL of CRLnative (**S6**), CRL-p(NIPAM) (**5**), CRL-p(ManEAA) (**6**), CRL-p(GalEAA) (**7**).*

Turbidity measurements

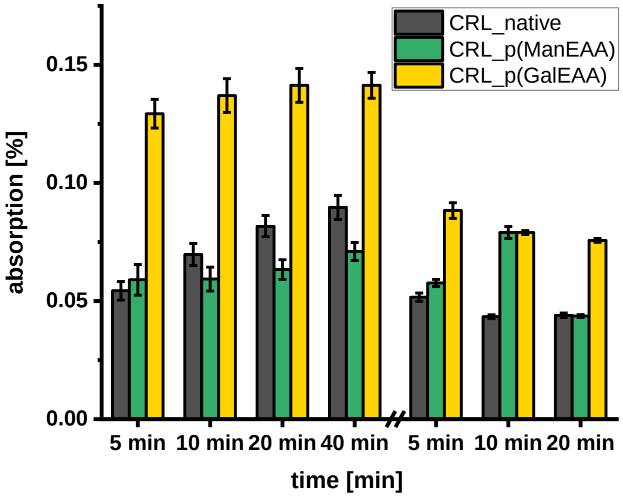

*Fig. S53: Turbidity measurements were performed at 420 nm at room temperature (20 °C). A. Competition-Inhibition of native CRL (**S6**) and CRL-conjugates **6** and **7** (5 µM). 120 µL of each structures were mixed with RCA_{120} (50 µM) and measured after 5, 10, 20, and 40 min, respectively. Inhibitor p(GalEAA) **S8** (30 mM) was then added and measured for another 5, 10, and 20 min.*

Activity measurements

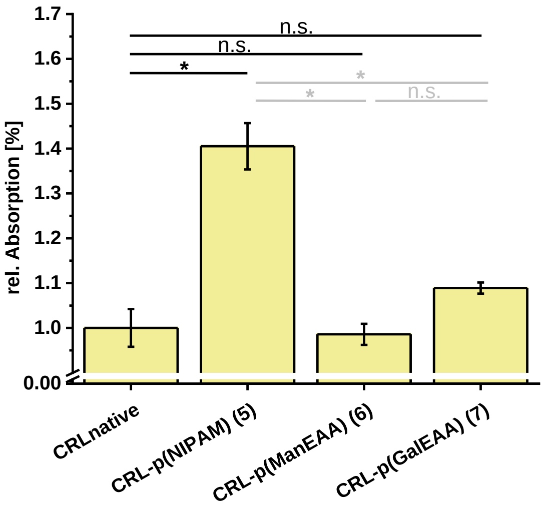

*Fig. S54: Illustration of the activity/ester cleavage of the enzyme CRL. All thiol detections were performed in reactions buffer: 0.1 M sodium phosphate, pH 8.0, containing 1 mM EDTA. Ellman's stock solution of 4 mg/mL Ellman's reagent and a 0.75 mM protein stock solution were prepared in reactions buffer. The assay was performed in triplicate measurements with an incubation time of 15 min and absorbance was measured at 412 nm. Comparison of the activity of the native enzyme CRL and the conjugates **5**-**7**. Data were evaluated using one-way ANOVA analysis followed by Bonferroni correction (*<0.05, not significant (n.s.)).*

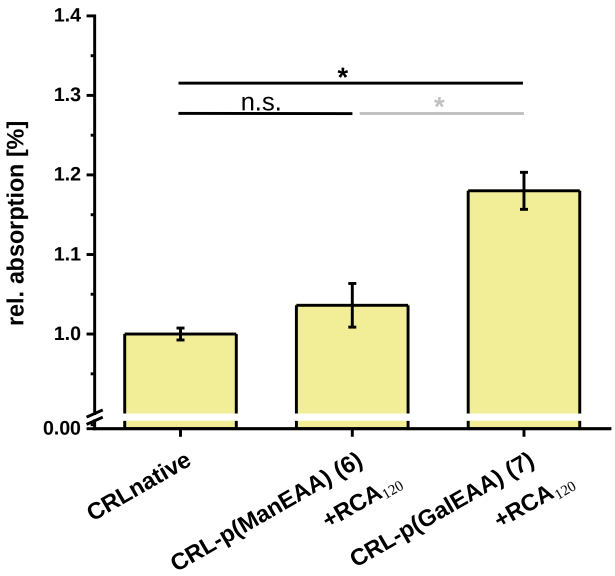

*Fig. S55: Illustration of the activity/ester cleavage of the enzyme CRL. All thiol detections were performed in reactions buffer: 0.1 M sodium phosphate, pH 8.0, containing 1 mM EDTA. Ellman's stock solution of 4 mg/mL Ellman's reagent and a 0.75 mM protein stock solution were prepared in reactions buffer. The assay was performed in triplicate measurements with an incubation time of 15 min and absorbance was measured at 412 nm. Comparison of native CRL with CRL-polymer conjugates **6** and **7** incubated with lectin RCA$_{120}$. Data were evaluated using one-way ANOVA analysis followed by Bonferroni correction (*<0.05 not significant (n.s.)).*

LCST measurement of CRL-p(NIPAM) conjugate 5

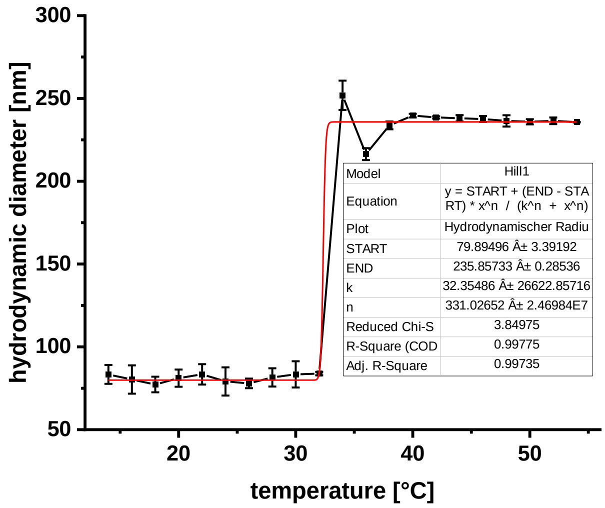

*Fig. S56: DLS measurement to analyze the LSCT of the CRL-p(NIPAM) conjugate **5**. The measurements were performed at a concentration of 0.5 mg/mL in PBS buffer (pH 7.4) in triplicates. The resulting LCST temperature is 32.4 °C.*

2.4 Synthesis of inhibitor polymers *S7* and *S8*

Synthesis of p(ManEAA) (S7) and p(GalEAA) (S8)

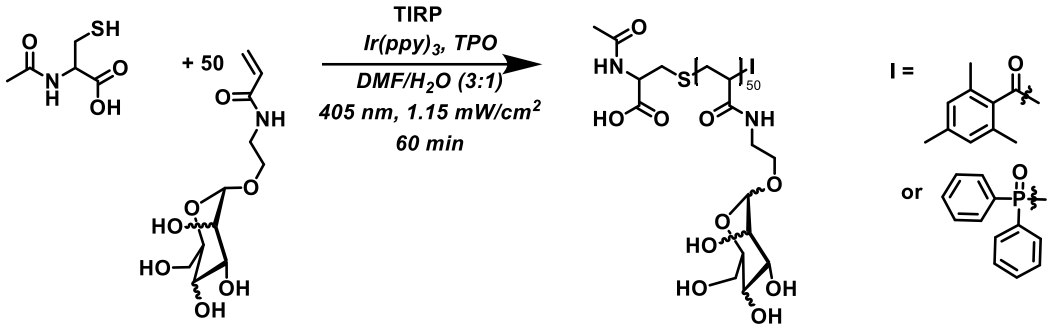

Solution I: 2.5 mg acetylcysteine (15.3 µmol) was dissolved in 2 mL DMF and both pre-dissolved TPO (5.43 mg, 15.3 µmol) in 100 µL DMF and 8.5 eq (35 mg, 130 µmol) TCEP in 0.5 mL DMF/H_2O (*v/v*) were added and deoxygenated for 20 min in a nitrogen stream. **Solution II:** Separately, 211.6 mg (766 µmol) of monomer ***S3*** and ***S4***, respectively, were dissolved in 2 mL of DMF and mixed with 0.25 mg Ir(ppy)$_3$ (0.38 µmol) in 100 µL of DMF and deoxygenated for 20 min under nitrogen flow. Solution I was irradiated at 1.15 mW/cm^2 for 2 min, then solution II was added to I and the entire mixture was irradiated at 1.15 mW/cm^2 for one hour. The resulting polymer was precipitated in cold acetone and purified via dialysis (cut-off: 1 kDa) and dried via freeze-drying. The products were isolated as white solids with yields of 83.4 mg (***S7***) and 101.8 mg (***S8***), respectively.

p(ManEAA) (S7):

^{1}H-NMR (600 MHz, D_2O, 297.9 K) δ (ppm) 9.99 – 7.58 (m, $H_{initiator}$), 4.97 – 4.84 (m, $H_{\alpha anomer}$), 4.05 – 3.22 (m, $H_{carbohydrates\ and\ side\ chain}$), 2.70 – 1.37 (m, $H_{backbone}$).

Aqueous SEC: (SEC buffer, pH = 7.0, 293,15 K, whole fraction, dn/dc = 0.153): Mn = 9.1 kDa, corresponds to 43 repeat units, Đ = 4.14, R_h = 7.5 nm.

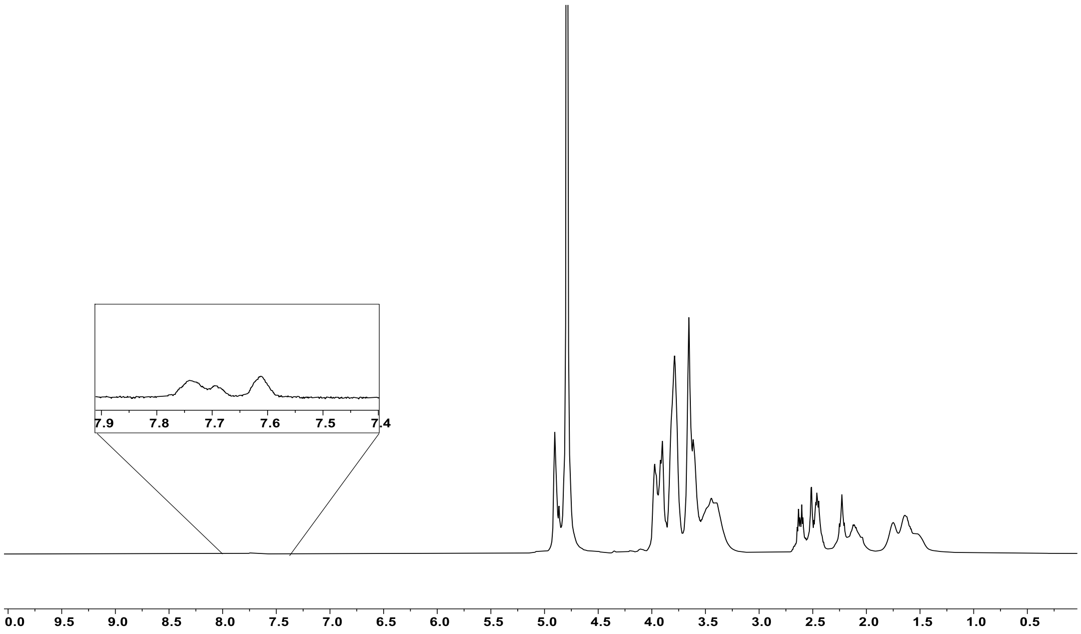

*Fig. S57: ^{1}H-NMR spectrum of p(ManEAA) (**S7**).*

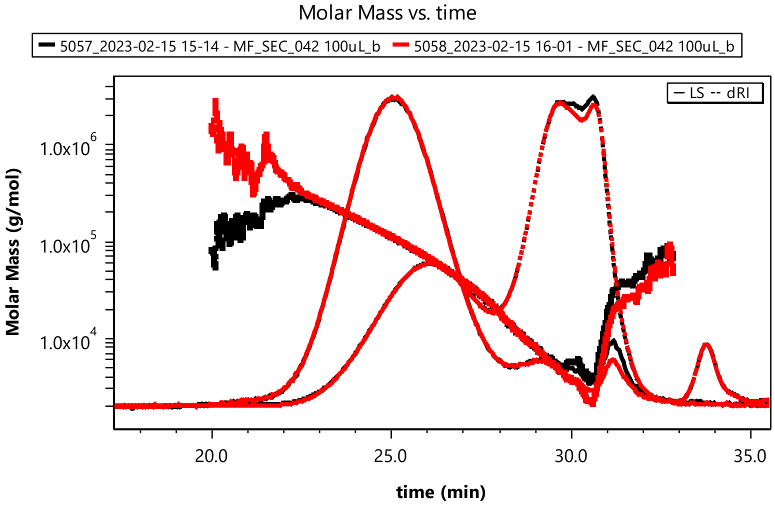

*Fig.S58: Aqueous SEC curve of p(ManEAA) (**S7**), LS and RI detector signal and molar masses, upswing of molar masses in late elution range is due to sample-column interaction.*

p(GalEAA) (S8):

^{1}H-NMR (600 MHz, D_2O, 297.9 K) δ (ppm) 8.14 – 7.55 (m, $H_{initiator}$), 4.51 – 4.35 (m, $H_{\beta anomer}$), 4.12 – 3.26 (m, $H_{carbohydrates\ and\ side\ chain}$), 2.72 – 1.39 (m, $H_{backbone}$).

Aqueous SEC: (SEC buffer, pH = 7.0, 293,15 K, whole fraction, dn/dc = 0.153): Mn = 7.8 kDa, corresponds to 37 repeat units, Đ = 5.08, R_h = 12.9 nm.

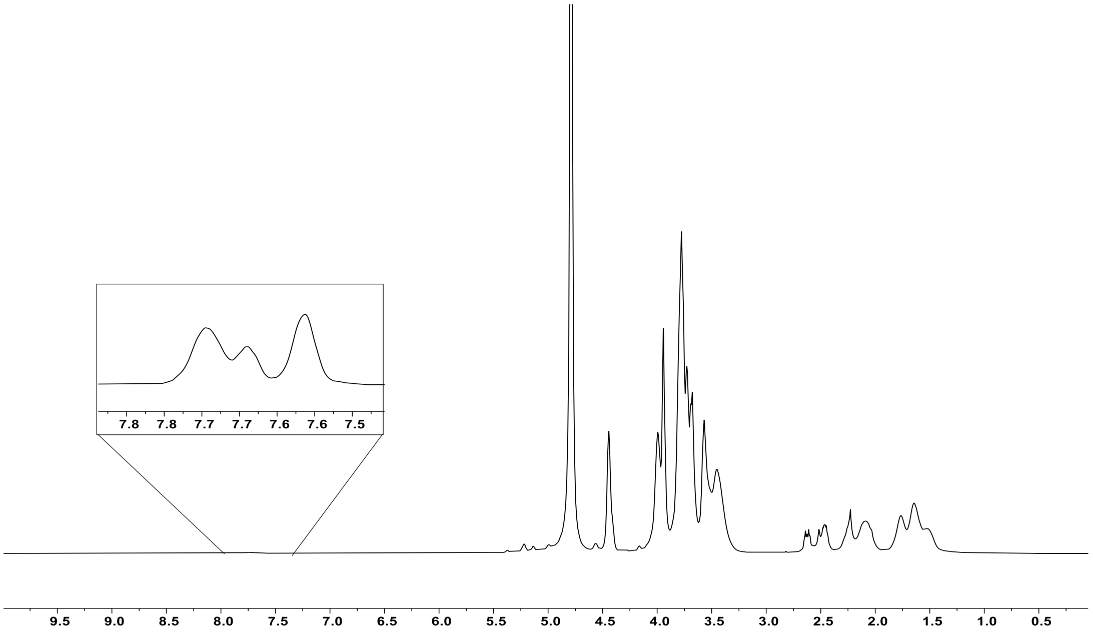

*Fig. S59: ^{1}H-NMR spectrum of p(GalEAA) (**S8**).*

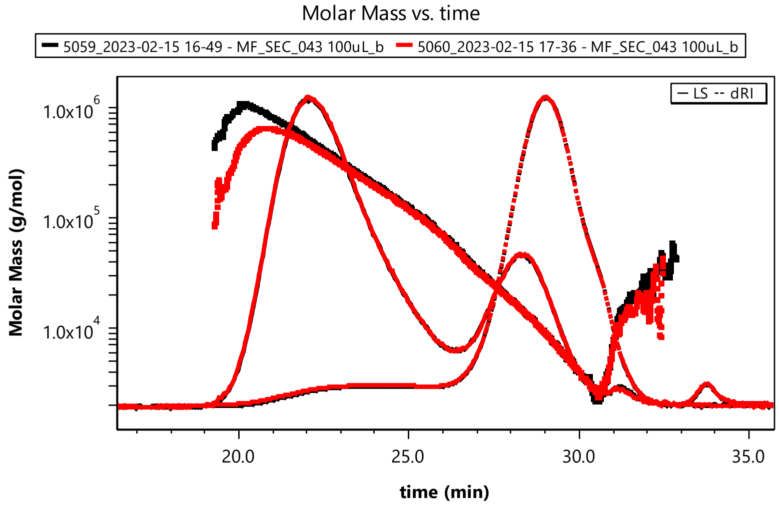

*Fig. S60: Aqueous SEC curves of p(GalEAA) (**S8**), LS and RI detector signal and molar masses, upswing of molar masses in late elution range is due to sample-column interaction.*

2.5 Carbohydrate-lectin binding studies on glass surfaces

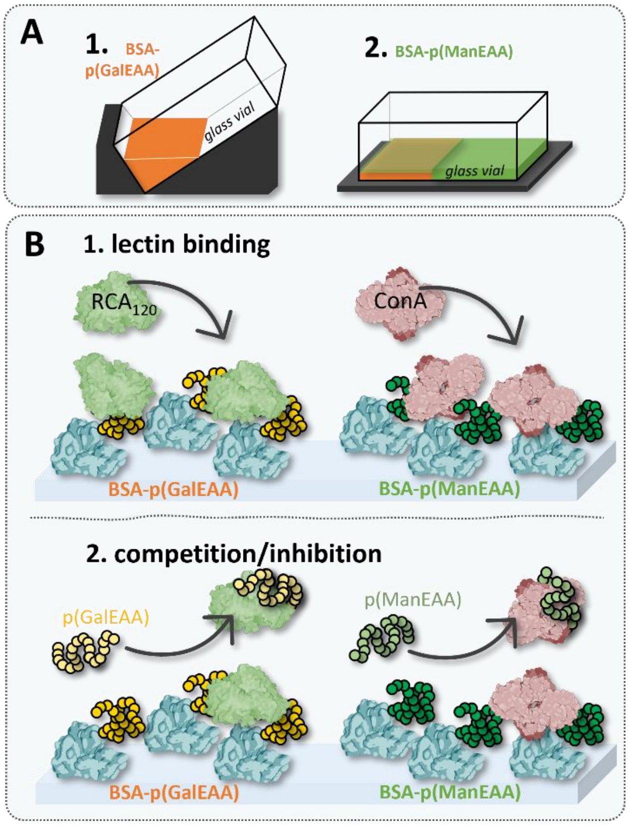

Fig. S61: ***A.*** *Schematic representation of the sample preparation.* ***B.*** *Schematic representation of lectin binding followed by competition/inhibition on the glass surface.*

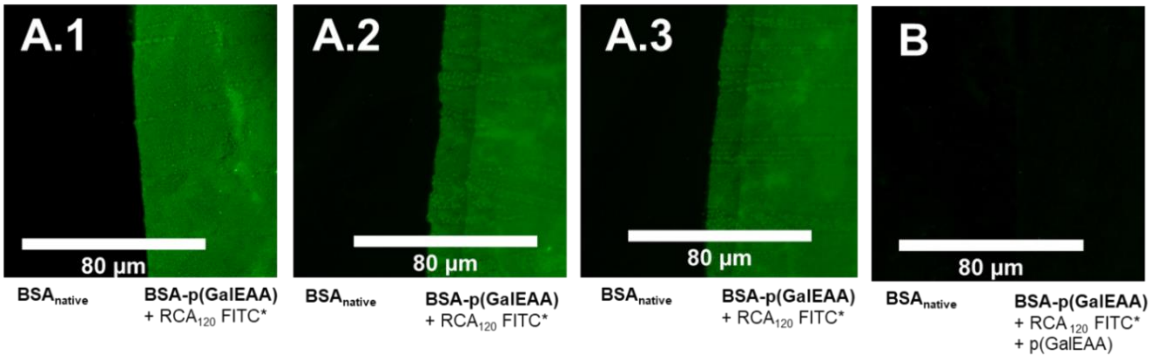

Fig. S62: Surface assay via half-side coating of glass. ***A.1-3*** *Presentation of native BSA (left) and galactose functionalized conjugate* ***4*** *(right) with bound fluorescently labeled (FITC) RCA_{120}.* ***B.*** *Imaging of inhibition of the sugar-lectin interaction on glass surface by using p(GalEAA)* ***S8****. (*Excitation: blue-light field 470 nm, contrast was adjusted for better visibility).*

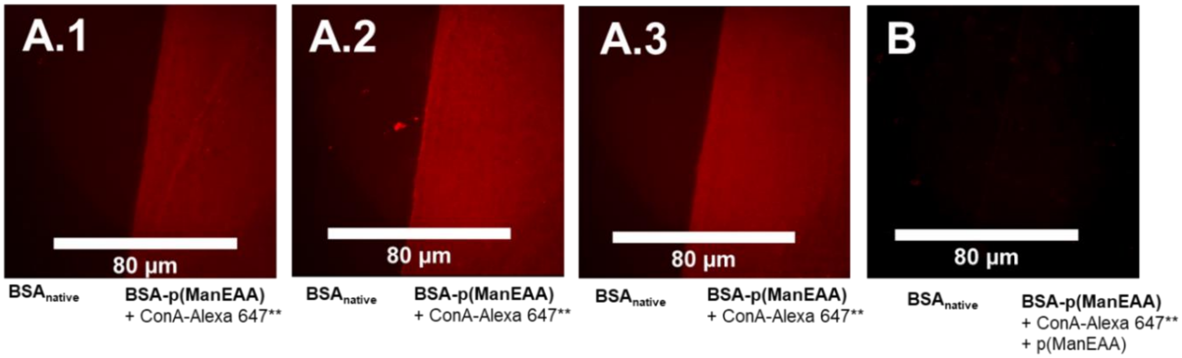

Fig. S63: Surface assay via half-side coating of glass. ***A.1-3*** *Presentation of native BSA (left) and mannose functionalized conjugate* ***3*** *(right) with bound fluorescently labeled (Alexa 647) ConA.* ***B.*** *Imaging of inhibition of the sugar-lectin interaction on glass surface by using p(ManEAA)* ***S7****. (**Excitation: red-light field 625 nm, contrast was adjusted for better visibility).*

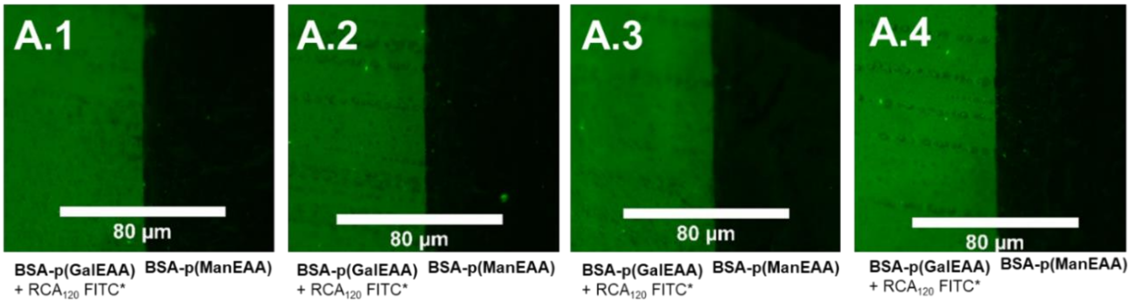

Fig. S64: Surface assay via half-side coating of glass. ***A.1-4*** *Presentation of galactose functionalized conjugate* ***4*** *(left) and mannose functionalized conjugate* ***3*** *(right) with bound fluorescently labeled (FITC) RCA_{120}.* ***B.*** *(*Excitation: blue-light field 470 nm, contrast was adjusted for better visibility).*

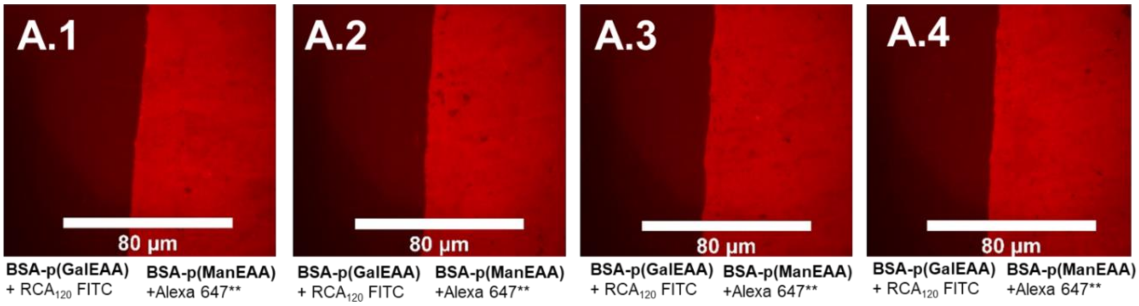

Fig. S65: Surface assay via half-side coating of glass. ***A.1-4*** *Presentation of galactose functionalized conjugate* ***4*** *(left) and mannose functionalized conjugate* ***3*** *(right) with bound fluorescently labeled (FITC) RCA_{120} and bound fluorescently labeled (Alexa 647) ConA. Both fluorescence-labeled lectins were detected on the surface, with only red light used for excitation, resulting in only the Alexa 647 dye of ConA emitting a fluorescent signal. (**Excitation: red-light field 625 nm, contrast was adjusted for better visibility).*

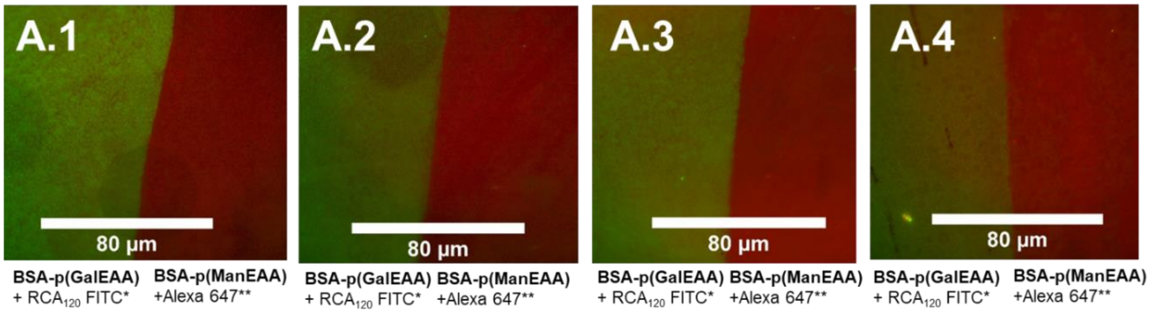

Fig. S66: Surface assay via half-side coating of glass. ***A.1-4*** *Presentation of galactose functionalized conjugate* ***4*** *(left) and mannose functionalized conjugate* ***3*** *(right) with bound fluorescently labeled (FITC) RCA_{120} and bound fluorescently labeled (Alexa 647) ConA. Both fluorescently labeled lectins were detected on the surface, and the images show an overlay of the fluorescence signals of both dyes. (*Excitation: blue-light field 470 nm, **Excitation: red-light field 625 nm, contrast was adjusted for better visibility).*

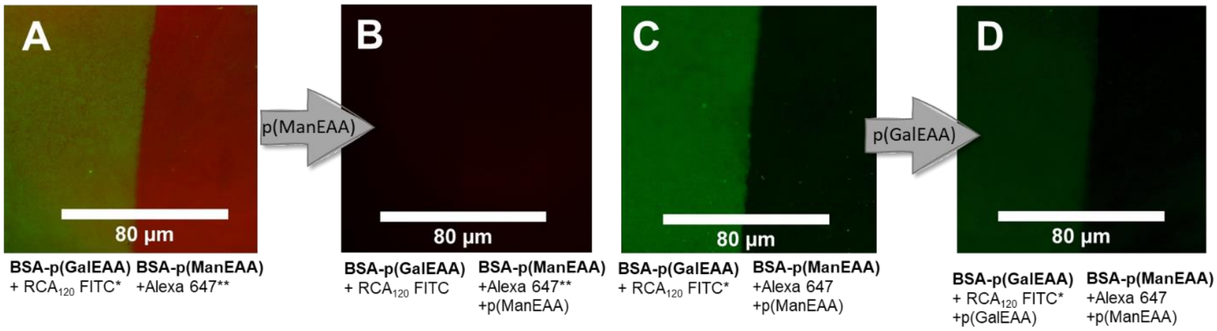

Fig. S67: ***A.*** *corresponds to figure* ***A.3*** *in Fig. S66. Due to the addition of the ConA-specific inhibitor* ***S7****, no significant fluorescence signal was detected on image* ***B*** *via the excitation in the red field. However, the fluorescence signal of the galactose-recognizing lectin RCA_{120}-FITC can still be detected in image* ***C*** *by excitation in the blue field. Image* ***D*** *shows the detachment of the galactose-recognizing lectin with the inhibitor* ***S8****, which was not detected by a significant fluorescence signal in the blue field. (*Excitation: blue-light field 470 nm, **Excitation: red-light field 625 nm, contrast was adjusted for better visibility).*

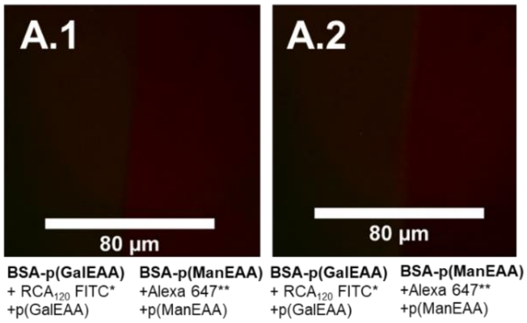

Fig. S68: Both images ***A.1*** *and* ***A.2*** *show the superimposed signals of the surfaces after inhibition with* ***S7*** *and* ***S8****. No significant fluorescence signal is visible on either side of the semi-coated surfaces. (*Excitation: blue-light field 470 nm, **Excitation: red-light field 625 nm, contrast was adjusted for better visibility).*

2.6 Thermal amino acid degradation

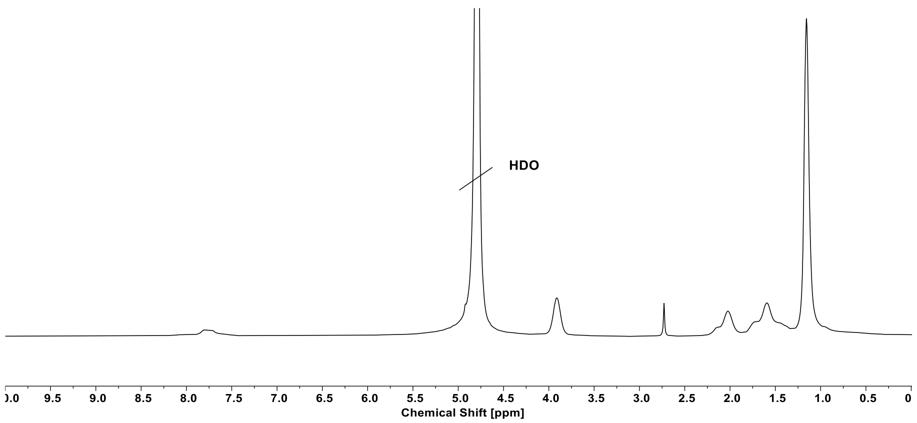

*Fig. S69: ^{1}H-NMR spectrum of thermal amino acid degradation of BSA-p(NIPAM) (**1**).*

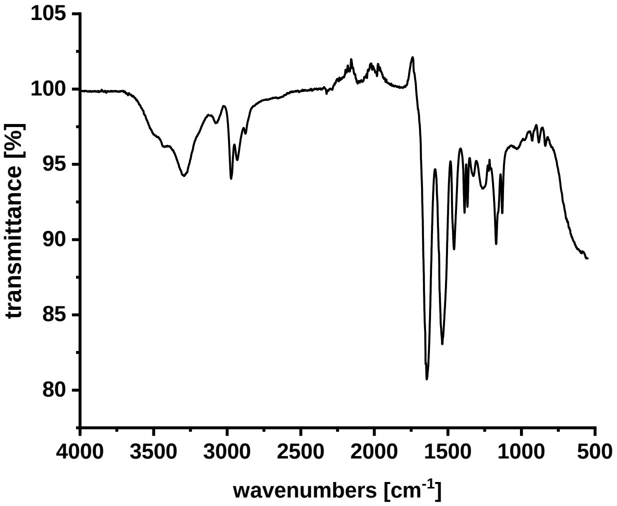

*Fig. S70: IR spectrum of thermal amino acid degradation of BSA-p(NIPAM) (**1**).*

References

[1] J. Janatova, J. K. Fuller, M. J. Hunter, *J Biol Chem* **1968**, *243*, 3612-3622.

[2] K. L. Heredia, D. Bontempo, T. Ly, J. T. Byers, S. Halstenberg, H. D. Maynard, *J Am Chem Soc* **2005**, *127*, 16955-16960.

[3] R. Margesin, G. Feller, M. Hammerle, U. Stegner, F. Schinner, *Biotechnol Lett* **2002**, *24*, 27-33.

[4] F. Schroer, T. J. Paul, D. Wilms, T. H. Saatkamp, N. Jack, J. Muller, A. K. Strzelczyk, S. Schmidt, *Molecules* **2021**, *26*.
